# Poly(imidazolyliden-yl)borato Complexes of Tungsten: Mapping Steric vs. Electronic Features of *Facially* Coordinating Ligands

**DOI:** 10.3390/molecules28237761

**Published:** 2023-11-24

**Authors:** Callum M. Inglis, Richard A. Manzano, Ryan M. Kirk, Manab Sharma, Madeleine D. Stewart, Lachlan J. Watson, Anthony F. Hill

**Affiliations:** Research School of Chemistry, Australian National University, Canberra, ACT 2601, Australia

**Keywords:** organometallic compounds, tungsten, carbyne, carbene

## Abstract

A convenient synthesis of [HB(HImMe)_3_](PF_6_)_2_ (ImMe = *N*-methylimidazolyl) is decribed. This salt serves in situ as a precursor to the tris(imidazolylidenyl)borate Li[HB(ImMe)_3_] pro-ligand upon deprotonation with *^n^*BuLi. Reaction with [W(≡CC_6_H_4_Me-4)(CO)_2_(pic)_2_(Br)] (pic = 4-picoline) affords the carbyne complex [W(≡CC_6_H_4_Me-4)(CO)_2_{HB(ImMe)_3_}]. Interrogation of experimental and computational data for this compound allow a ranking of familiar tripodal and facially coordinating ligands according to steric (percentage buried volume) and electronic (ν_CO_) properties. The reaction of [W(≡CC_6_H_4_Me-4)(CO)_2_{HB(ImMe)_3_}] with [AuCl(SMe_2_)] affords the heterobimetallic semi-bridging carbyne complex [WAu(μ-CC_6_H_4_Me-4)(CO)_2_(Cl){HB(ImMe)_3_}].

## 1. Introduction

**Scheme 1:**
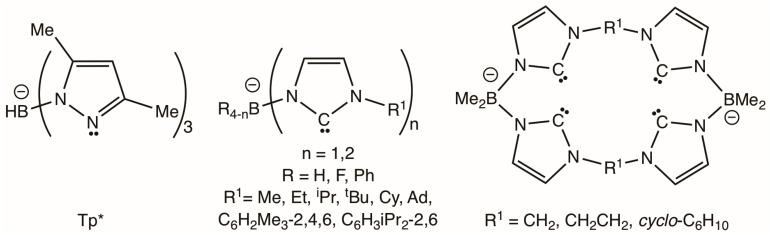
Selected pyrazolyl and imidazolylidenyl borates. Tp* = hydrotris(dimethylpyrazolyl)borate.

The poly(pyrazolyl)borate class of chelates developed by Trofimenko, colloquially known as ‘scorpionates’ [1,2,3], have found broad application in diverse of areas of coordination and bioinorganic and organometallic chemistry. Key features that have contributed to their widespread deployment include (i) ease of synthesis; (ii) functionalization at both the bridgehead boron and pyrazolyl rings to provide a range of steric and electronic properties; (iii) kinetic stability of the chelated cage once coordinated to a metal centre; (iv) their so-called ‘octahedral enforcer’ nature, whereby the topology of the cage especially favours octahedral coordination geometries; and (v) the extension of the principle to the replacement of the pyrazol-1-yl arms with a range of other heterocycles that bridge boron and the metal to which they coordinate. Amongst these, the hydrotris(3,5-dimethylpyrazol-1-yl)borate ligand (HB(pzMe_2_)_3_, Scheme 1) has proven to be especially useful in presenting a moderate degree of steric protection to the remaining three ligands in an octahedral metal complex.

**Scheme 2:**
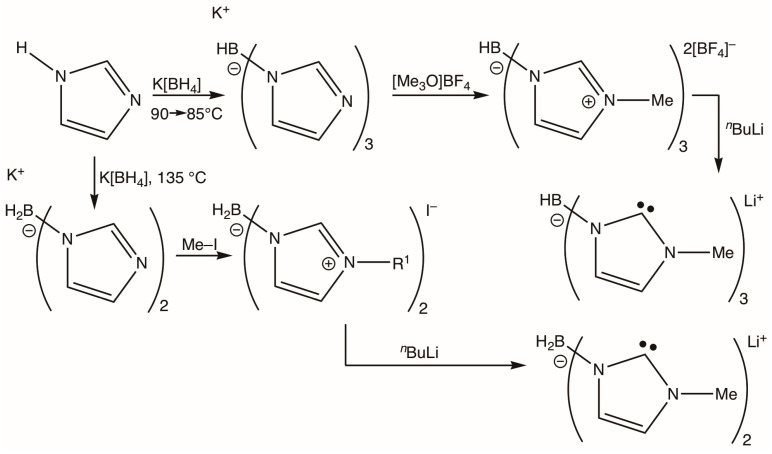
Fehlhammer’s syntheses of bis- and tris(*N*-methylimidazolylidenyl)borates [3].

*N*-heterocyclic carbenes (NHC) have emerged over the last three decades, from being rather niche ligands of fundamental interest, to highly effective supporting co-ligands for the development of robust materials and, in particular, catalysts [4,5,6]. Fehlhammer first demonstrated the confluence of poly(azolyl)borate and NHC chemistries with reports of the first tris(*N*-alkylimidazolylidenyl)borates (HB(ImR^1^)_3_, R^1^ = Me, Et, *^i^*Pr; Scheme 2) [7,8,9,10], and whilst the trimethyl derivative HB(ImMe)_3_^−^ most closely resembles the topology of the Tp* scorpionate, its chemistry has been scarcely developed beyond the original Fehlhammer work. Rather, the ligand class has been functionally elaborated to include (i) sterically imposing *N*-subtituents (R^1^ = *^t^*Bu, Cy, adamantly, mesityl and 2,6-diisopropylphenyl) [11,12,13,14], (ii) macrocyclic variants [15,16,17,18,19,20,21], (iii) extension to bidentate examples [5,22,23,24,25,26,27,28,29,30,31,32,33,34], (iv) replacement of the bridgehead borohydride with phenyl or fluoro groups [35,36,37] and (v) substitution of the imidazolylidene bridges by triazolylidenes or benzoimidazolylidenes [35,36,37,38].

Amongst the multitude of catalytic processes catalysed by NHC-supported mediators, the advent of Grubbs’s second generation alkene metathesis catalyst and related analogues [39,40,41,42] has led to a plethora of complexes that feature both NHC and conventional alkylidene ligands. These serve to demonstrate the vastly different nature and reactivity of the metal–carbon ‘multiple’ bonds, whereby productive metathesis involves the alkylidene ligand exclusively, while the NHC ligand remains innocent. That said, an early report by Lappert described the metathesis of electron-rich alkenes by an NHC complex devoid of alkylidene ligands [43]. In contrast, alkylidyne complexes with metal–carbon triple bonds that are supported by NHC ligands are somewhat scarcer [44,45,46,47,48,49,50,51,52,53,54,55,56,57] with most examples having emerged from the groups of Esteruelas and Buchmeiser. The intersection of poly(imidazolylidenyl)borates with the chemistry of metal–carbon multiple bonds would appear limited to a single macrocyclic complex [Fe(=CPh_2_)({Me_2_B(C_3_N_2_H_2_)_2_C_6_H_10_}_2_)] [21]. Given the important role that poly(pyrazolyl)borate ligands have played in the development of alkylidyne chemistry [58], herein we report the first carbyne complex ligated by a poly(imidazolylidenyl)borate, [W(≡CC_6_H_4_Me-4)(CO)_2_{HB(ImMe)_3_}] (HB(ImMe)_3_ = hydrotris(3-methylimidazoylyliden-1-yl)borate) which provides an opportunity to benchmark the donor properties of the HB(ImMe)_3_ ligand against more familiar tripodal tridentate ligands. The complex also serves as a precursor to the first heterometallic complex of a poly(imidazolylidenyl)borate viz. [WAu(μ-CC_6_H_4_Me-4)Cl(CO)_2_{HB(ImMe)_3_}].

## 2. Results

### 2.1. Pro-Ligand Synthesis

Fehlhammer’s original synthetic approach (Scheme 2) [7] involved threefold alkylation of potassium hydrotris(imidazol-1-yl)borate with Meerwein’s salt [Me_3_O]BF_4_, this latter reagent being the most expensive component. Apart from blazing the original trail, Fehlhammer’s approach allows for the installation of various carbene alkyl *N*-substituents at a late stage on a common late synthetic intermediate.

**Scheme 3:**
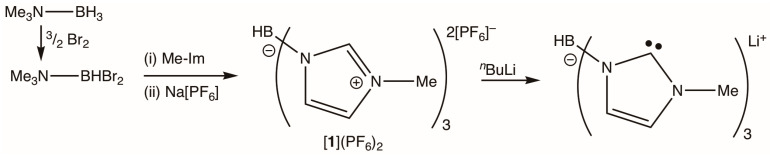
Alternative syntheses of tris(*N*-methylimidazolylidenyl)borate salts.

We have developed an alternative synthesis that borrows from protocols developed for more sterically encumbered examples described by Smith [11,12,13,14]. Whilst demonstrating no new principles here, our approach does offer both convenience and economy, employing cheap commercially available reagents (Scheme 3).

**Figure 1:**
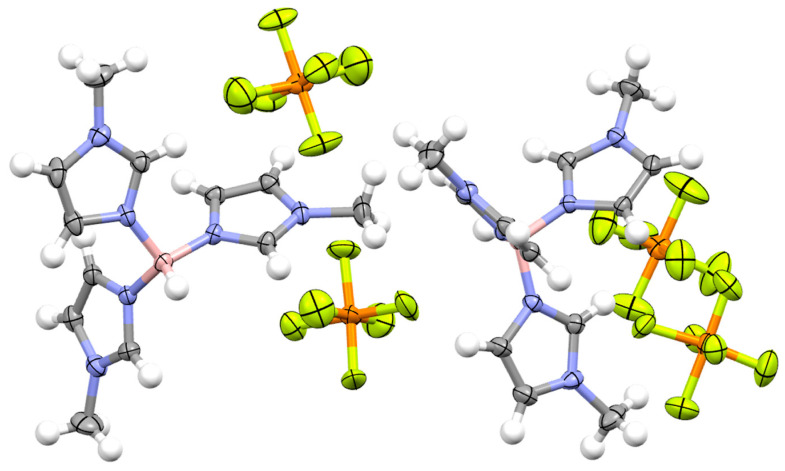
Structure of the hydrotris(*N*-methylimidazolyl)boronium salt [HB(ImMeH)_3_](PF_6_)_2_ ([1](PF_6_)_2_ (two crystallographically independent molecules shown, 50% displacement ellipsoids, major occupancies of positionally disordered PF_6_ anions shown).

The reaction of [Me_3_N^.^BH_3_] with bromine affords [Me_3_N^.^BHBr_2_] [59], which may be generated in situ without isolation. Subsequent treatment with *N*-methylimidazole affords the salt [HB(ImMeH)_3_]Br_2_ ([1]Br_2_). This salt, whilst forming in high yields, is difficult to manipulate as it is exceedingly deliquescent and upon filtration under ambient air rapidly forms a sticky syrup. This behaviour is potentially problematic since the subsequent step calls for deprotonation via strong, moisture-sensitive bases, e.g., *^n^*BuLi or KN(SiMe_3_)_2_. Metathesis with aqueous Na[PF_6_], however, results in ready recovery of the hexafluorophosphate salt [HB(ImMeH)_3_](PF_6_)_2_ ([1](PF_6_)_2_), which is not hygroscopic and crystallizes free of water as confirmed via a crystallographic analysis (Figure 1).

### 2.2. Ligand Installation

**Scheme 4:**
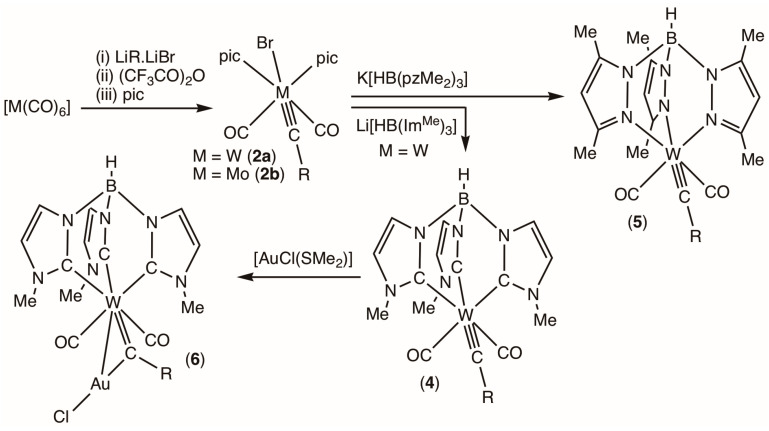
Synthesis of mono- and bi-metallic toluidyne complexes ligated by the HB(Im^Me^)_3_ ligand (R = C_6_H_4_Me-4, pic = 4-picoline = NC_6_H_4_Me-4).

For installation of the pro-ligand on a suitable alkylidyne precursor, the 4-toluidyne complex *trans*,*cis*,*cis*-[W(≡CC_6_H_4_Me-4)(CO)_2_(pic)_2_Br] (pic = 4-picoline) (**2a**) was chosen to exploit the lability of the bromide and 4-picoline ligands. Whilst this complex has not been previously reported on, its synthesis (Scheme 4) is unremarkable and mirrors that of the known xylyl or mesityl analogues [60,61,62]. Synthetic procedures are presented alongside those for the molybdenum analogue (**2b**) in the Experimental section in addition to a crystallographic analysis.

The pro-ligand salt [1](PF_6_)_2_ was dissolved in tetrahydrofuran and cooled (dry ice/propanone) before addition of 3 equivalents of *^n^*BuLi, followed by slow warming to room temperature to provide a yellow solution of Li[HB(ImMe)_3_] (Li [3]) which was re-cooled and treated with **2a**. Re-warming to room temperature resulted in a colour change to dark brown as the infrared absorptions for the starting material (**2a**: ν_CO_ = 1986, 1898) were replaced with those of the new product (**4**: ν_CO_ = 1958, 1873 cm^−1^). After stirring for 3 h, the product was isolated via column chromatography to yield a bright orange microcrystalline powder.

Spectroscopic data were consistent with the formulation of the desired product [W(≡CC_6_H_4_Me-4)(CO)_2_{HB(ImMe)_3_}] (**4**). Amongst these, the most conspicuous datum is that for the carbyne resonance in the ^13^C{^1^H} NMR spectrum (CD_2_Cl_2_: δ_C_ = 280.7, ^1^*J*_WC_ = 171.4 Hz). Consistent with the inferred *C*_s_ symmetry of the molecule, the carbonyls gave rise to a single resonance (δ_C_ = 223.3, ^1^*J*_WC_ = 132.0 Hz) while the tungsten-bound carbon nuclei of the NHC donors gave rise to two resonances at a ratio of 2:1 with markedly different chemical shifts and ^1^*J*_WC_ couplings (δ_C_/^1^*J*_WC_ = 192.0/95.2), 181.3/44.7). With the exception of the complexes [Pt{H_2_B(ImR^1^)_2_}_2_] (R^1^ = Me, Et) for which ^1^*J*_PtC_ values were not reported [8], and [Rh(CO)(L){X_2_B(ImR)_2_] (L = CO, PPh_3_, PCy_3_; X = H, F; R = Ph, Cy) [31], poly(imidazolylidenyl)borates have not previously been coordinated to metal nuclei with usefully spin-active (*I* = ½) isotopes.

**Figure 2:**
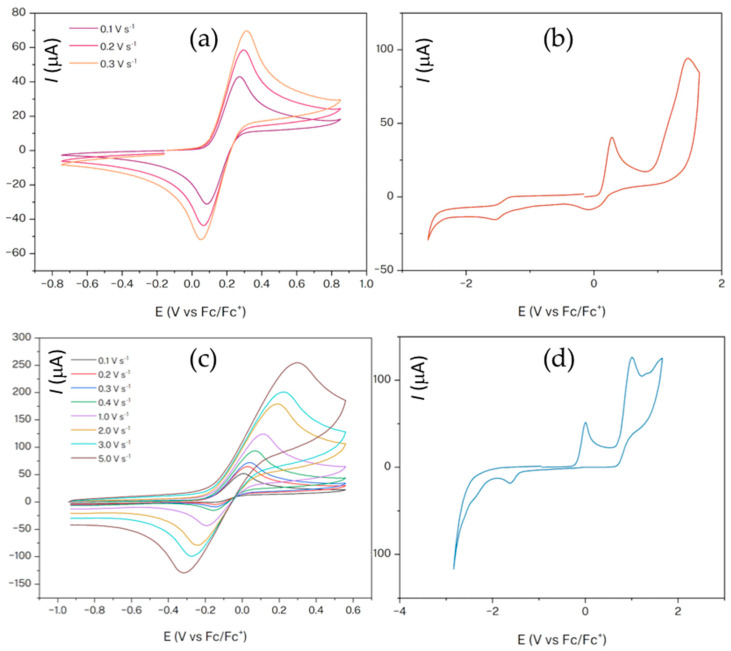
Cyclic voltammetry of [W(≡CC_6_H_4_Me-4)(CO)_2_(L)] (L = HB(pzMe_2_)_3_ **5**, HB(ImMe)_3_ **4**) (Silver wire *pseudo*-reference electrode, anaerobic 1 mM in CH_2_Cl_2_, 0.1 M [NBu_4_][PF_6_] supporting electrolyte; ferrocene reference *E*_1/2_ = 0.460 V cf. Ag/Ag^+^ = 0). (**a**) Reversibility CV at varied scan rates of **5** (0 V → +1.0 V → –0.6 V). (**b**) Full window CV of **5** (0 V → +1.8 V → –2.5 V, υ = 0.1 Vs^−1^). (**c**) Reversibility CV at varied scan rates of **4** (0 V → +0.9 V → –0.6 V). (**d**) Full window CV of **4** (0.6 V → +2.0 V → –2.5 V, υ = 0.1 Vs^−1^).

As **4** is the first tungsten complex of such a ligand, it provides an opportunity to demonstrate the special feature of HB(ImR^1^)_3_ chelates cf. poly(pyrazolyl)borates; scalar couplings observed in the ^13^C{^1^H} NMR spectra may serve as reporters to interrogate metal–carbon bonding. Thus, whilst the chemical shift and associated coupling for the carbon nuclei trans to the carbonyl ligands are unremarkable (e.g., cf. the conventional NHC complex [W{=C(ND*^i^*PP)_2_C_2_H_2_}(CO)_5_]: δ_C_ = 187.9, ^1^*J*_WC_ = 105.7 Hz, DiPP = C_6_H_3_*^i^*Pr_2_-2,6) [63], the resonance for the carbon trans to the carbyne is shifted some 11 ppm to higher field and displays a dramatically reduced coupling to tungsten-183 (44.7 Hz). These may be taken as indicating a weaker W–C interaction which in turn reflects the pronounced trans influence of the alkylidyne ligand, a feature well-documented in the structural chemistry of alkylidyne complexes ligated via poly(pyrazolyl)borate ligands [58]. As to the impact of the HB(ImMe)_3_ ligand on the remaining co-ligands, comparison with the known complex [W(≡CC_6_H_4_Me-4)(CO)_2_(Tp*)](**5**) [64] (Tp* = hydrotris(dimethylpyrazoyl)borate, prepared here from K[Tp*] and **2a**, see Experimental) is useful. The carbyne and carbonyl resonances for the Tp* derivative appeared at almost identical frequencies to those of the HB(ImMe)_3_ complex [δ_C_(^1^*J*_WC_/Hz) = 279.2 (186.6), 224.0 (166.2)]; however, in both cases, the magnitudes of ^1^*J*_WC_ values were significantly larger for **5** than for **4**. Insofar as these may be taken as being indicative of the strength of the metal–carbon interaction, it would appear that the NHC donors weaken both the carbyne and carbonyl binding. This is, however, difficult to reconcile with the ν_CO_-associated infrared data which comprise *A*_1_ and *B*_1_ modes observed at 1958 and 1873 cm^−1^ in dichloromethane (ATR: 1949, 1867 cm^−1^). These are amongst the lowest observed for neutral complexes of the form [W(≡CC_6_H_4_Me-4)(CO)_2_(L)] where L is one of a range of nominally tripodal facially capping ligands [58,64,65,66,67]. These values are even lower than for the π-donor ligand HB(mt)_3_ (1967, 1875 cm^−1^; mt = 2-mercapto-*N*-methyl-imidazol-1-yl) [67] and Kläui’s (η^5^-C_5_H_5_)Co(PO_3_Me_2_)_3_ ligand [68]. It would therefore appear that the HB(ImMe)_3_ ligand makes the tungsten centre especially electron rich and this may be verified using cyclic voltammetry (Figure 2). For both **4** and **5**, sweeping the voltage to *ca* +2 V reveals two oxidation processes, neither of which appear reversible. Limiting the sweep to *ca* 1.0 V indicates that the reversibility of first oxidation event increases with increasing sweep rate. For **5**, Δ*E*_p_ increases slightly with increased scan rate from 0.180 (0.1 Vs^−1^) to 0.250 V (0.3 Vs^−1^) suggesting the oxidation is essentially reversible with *E*_½_ = 0.34 V (*E*_p,c_ = 0.43 at 0.1 Vs^−1^). For **4** the dependence of Δ*E*_p_ on sweep rate is more significant, increasing from 0.170 V at 0.1 Vs^−1^ (*E*_p,c_ = 0.33 V) to 0.630 V at 5 Vs^−1^(*E*_p,c_ = 0.64 V) is observed. Thus, fast sweep rates are required to observe a reasonable degree of reversibility, with, however, an almost identical half-wave potential (*E*_½_ 0.345 V) to that of **5**. Chemical oxidation of tris(pyrazolyl)borate carbyne complexes of tungsten is typically accompanied by decarbonylation [65,69,70,71], which most likely accounts for the poor reversibility at slow sweep rates or higher voltages.

### 2.3. Quantification of Steric and Electronic Features

A popular and time-honoured method for assessing the donor properties of ligands involves their impact on infrared frequencies of carbonyl co-ligands. This is traditionally assayed, in the case of phosphines, using the Tolman electronic parameter ν^T^, viz. the frequency of the *A*_1_ mode of CO vibrations in a host of complexes of the form [Ni(L)(CO)_3_] [72]. Although similar scales may be developed for NHC ligands coordinated to the ‘Ni(CO)_3_’ fragment [73,74,75], the toxicity of nickel carbonyl has led to the advent of alternative scales based on the RhCl(CO)_2_ fragment (average of *A*_1_ and *B*_1_ modes) as the preferred platform, alongside metrics derived from NMR data for the NHC bound to selenium (=Se, δ_Se_), phenylphosphinidine (=PPh, δ_P_) or PdBr*_2_*{C(N*^i^*Pr)_2_C_6_H_4_} (δ_C_) fragments [76]. These methods are not directly applicable to H_n_B(ImR^1^)_4-n_ complexes due to their negative charge and chelation. While it would be reasonable to presume that, as with conventional neutral NHC ligands, these will be potent net donors, it would be useful to be able to benchmark both the electronic and steric features of poly(imidazolylidenyl)borate ligands against those of more familiar facially capping nominally tridentate (κ^3^, η^5^ or η^6^) ligands, of which there are many. Smith has already suggested such a ranking for a small number of facial/tripodal ligands based on the ν_NO_ stretching frequencies of complexes of the form [Ni(NO)(L)] [37]. Such ligands may be grouped according to their charge (neutral, mono- or di-anionic) which in turn impacts the charge of the derived complexes (cationic, neutral or anionic, respectively). In the case of complexes of the form [W(≡CC_6_H_4_Me-4)(CO)_2_(L)]^x+^, a number of these have been compared in terms of the experimentally determined infrared data for the cis-dicarbonyl oscillator [67,77,78,79,80,81,82,83,84,85,86,87,88,89]. In addition to the frequencies of the observed symmetric and antisymmetric modes (*A*_1_ ν_s(CO)_, *B*_1_ ν_as(CO)_), the two numbers may be condensed into a singular Cotton–Kraihanzel force constant [90]. This is reasonable in the case of [W(≡CR)(CO)_2_(L)]^x+^ because the two carbonyl ligands are chemically equivalent, i.e., both individual CO oscillators are identical. This is perhaps less appropriate in the ‘RhCl(CO)_2_’ system, where in any event the simple arithmetic mean is usually employed.

Our previous collation was based on experimentally determined ν_CO_ values with the caveat that some were acquired from solid-state mesurements (Nujol mulls, KBr discs, ATR, etc.) while others were obtained from a variety of solvents. Infrared data for metal carbonyls are prone to significant perturbation in the solid state due to different crystal modifications or crystallographically independent molecules within the same crystal which in each case place the CO ligand(s) in different environments. The solvent-dependent nature of IR data for metal carbonyls, due to which both the frequency and broadening are significantly impacted by the choice of solvent, has long been recognized [91]. Thus, gas phase data, when measurement is viable, typically produce higher frequencies than are found in aliphatic hydrocarbons, and while such solvents provide the sharpest and therefore best-resolved peaks, comparatively few carbonyl complexes are sufficiently soluble. Dichloromethane has therefore become the solvent of choice offering the most accommodating solubility characteristics and reasonably narrow peaks.

**Figure 3:**
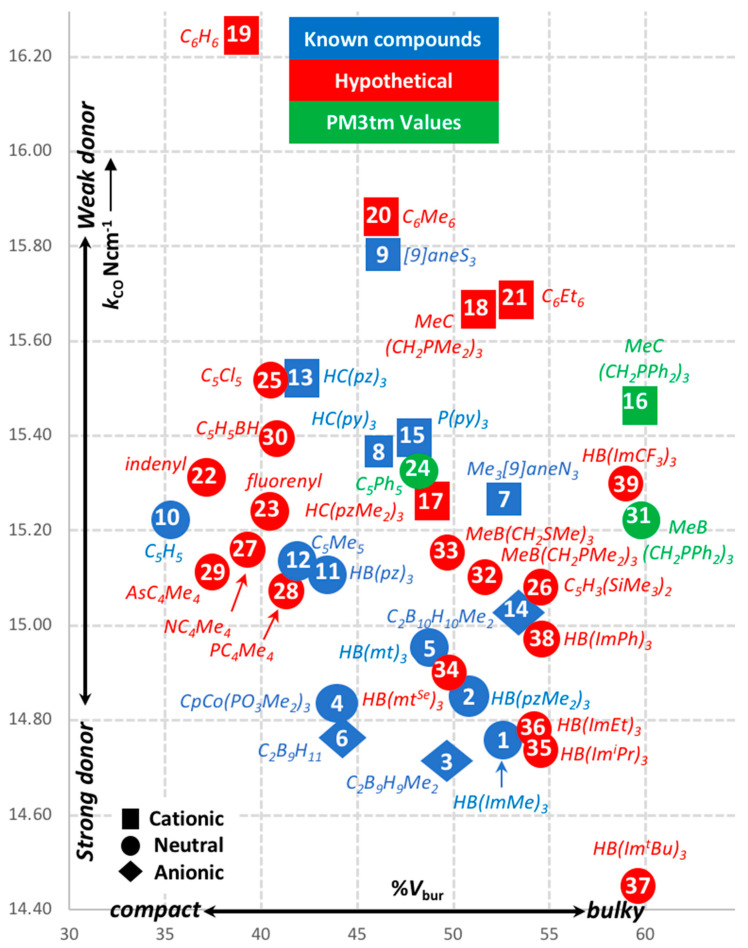
Electronic (*k*_CO_) vs. steric (%*V*_bur_) map for a range of facially coordinating ligands derived computationally (DFT:ωB97X-D/LANL2Dζ(W)). A small number (shown in green) were calculated at the semi-empirical PM3tm level of theory due to their large atom count, for which the ordinate positions should be treated with appropriate reservation.

To obviate these imponderables, we have collated infrared data for a range of complexes [W(≡CC_6_H_4_Me-4)(CO)_2_(L)]^x+^ derived from computational interrogation (Table 1). Our intention is *not* to provide the most precise current state-of-the-art investigation of the intimate bonding and thermodynamic properties of such complexes but rather to derive a readily accessible and computationally economic comparative scale. A useful corollary of this approach is that the optimised geometries used for frequency calculations may be employed to directly calculate the percentage buried volume (%*V*_bur_) [92,93] of each ligand L. The %*V*_bur_ approach to quantifying the steric impact of a ligand is especially suitable for ligands with irregular topologies, and for phosphines, such analysis reassuringly returns a correlation approximately linear with Tolman’s cone angle (θ^T^ = 3.95x%*V*_bur_ + 31.5) [94]. Accordingly, a scatter plot of the Cotton–Kraihanzel force constant *k***_CO_** vs. %*V*_bur_ (Figure 3) may be presented for ligands L that is reminiscent of the familiar ν^T^ vs. θ^T^ plot used to map phosphine electronic and steric space [72]. For this purpose, with this combination of density functional, basis set and anharmonic scaling factor the value of the Cotton–Kraihanzel force constant reduces to the following equation:

*k*_CO_ [Ncm^−1^] = 1.7426 × 10^−6^ Ncm × (ν_s_^2^ + ν_as_^2^)

**Figure 4:**
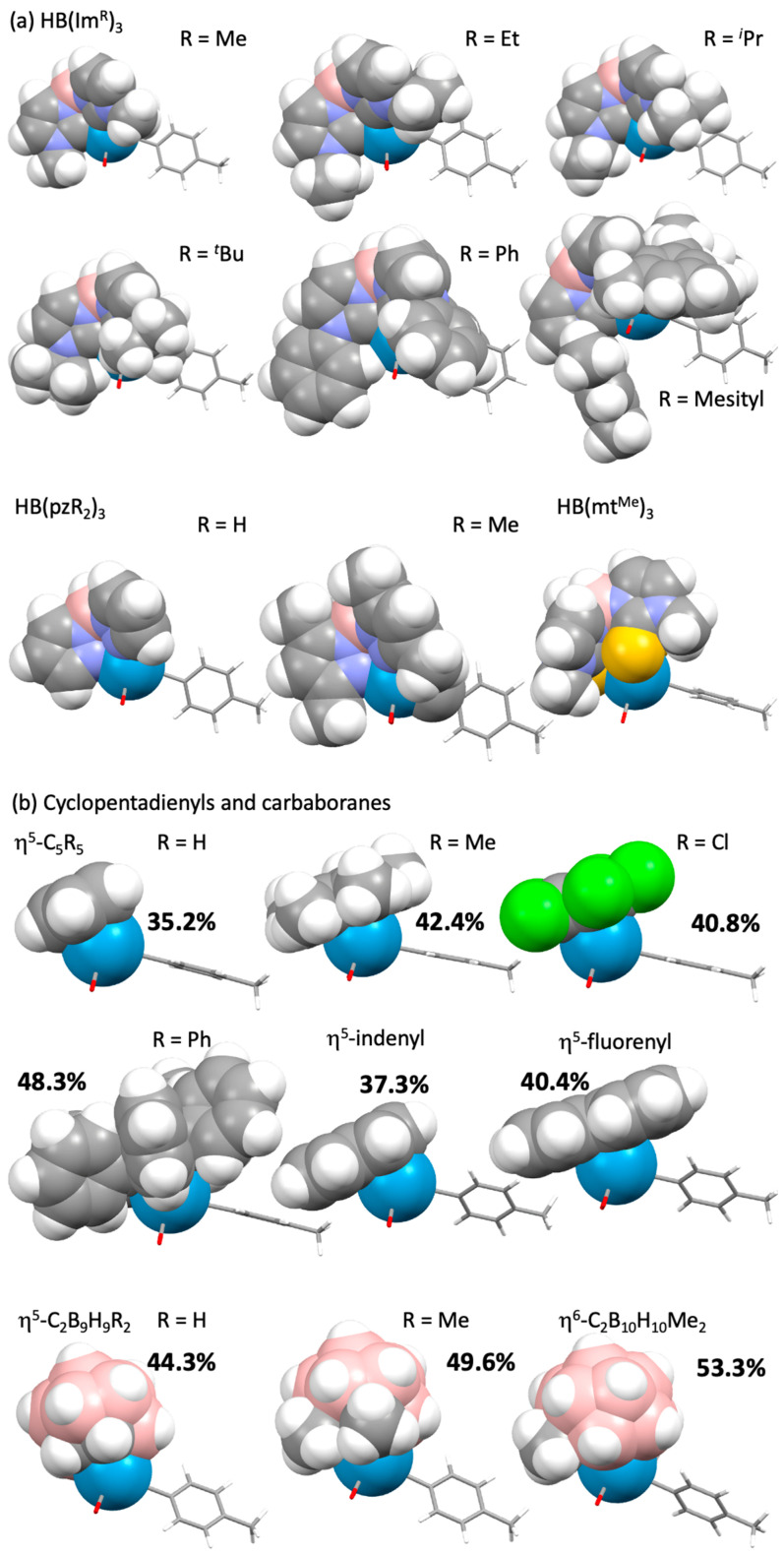

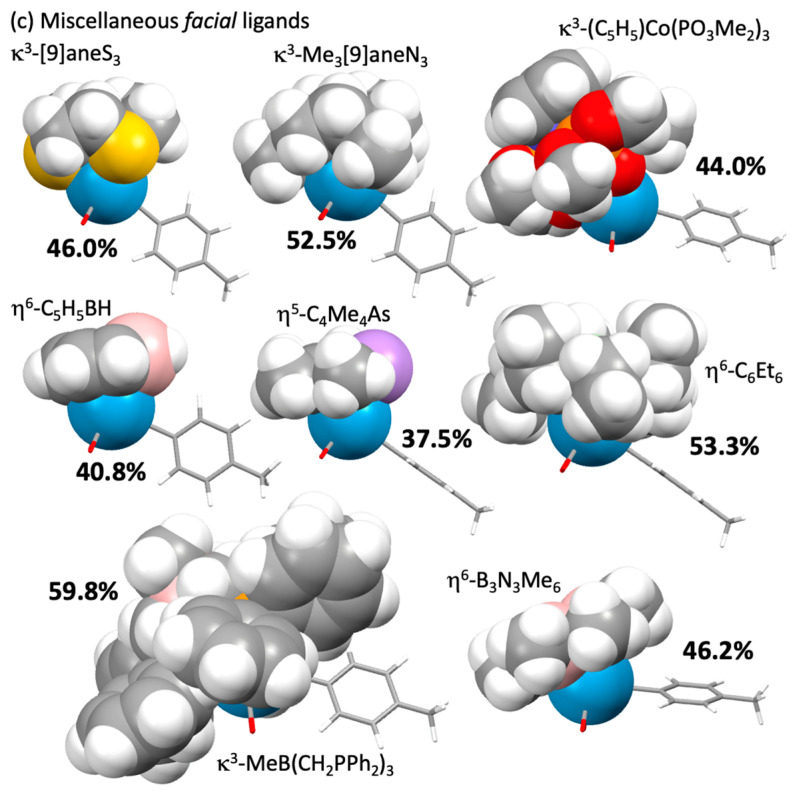
Corey–Pauling–Koltun representations of facial ligand from Table 1 and Table 2 in the complexes [W(≡CC_6_H_4_Me-4)(CO)_2_(L)] toluidyne and carbonyl ligands simplified). (**a**) Poly(azolyl)borates; (**b**) cyclopentadienyls and carbaboranes; (**c**) miscellaneous facial ligands.

The ωB97X-D [95,96] functional was employed with the 6-31G* basis set [97] in combination with the LANL2Dζ effective core potential for tungsten [98,99,100], and while much more sophisticated levels of theory are certainly available, this selection represents a balance between utility and computational economy for these medium-sized molecules. For larger ligands ‘L’, where steric bulk has or might be an intentional design feature, %*V*_bur_ values obtained at the simpler semi-empirical PM3tm level of theory are used, as we are here only concerned with molecular topologies (Figure 4). Taking complexes of the ligands HB(pzMe_2_)_3_, HB(ImMe)_3_ and MeC(CH_2_PMe_2_)_3_ as test cases, the variation in %*V*_bur_ calculated between ωB97X-D/6-31G*/LANL2Dζ and PM3tm methods was <3%, i.e., within the magnitude of molecular libration. Vibrational frequencies, whilst calculated to ensure local minima had been located, were imprecise at the PM3tm level and considered of little use. Accordingly, the ordinate location of such ligands in Figure 3 (shown in green) should be viewed with considerable caution. These were derived with little rigour by simply scaling the PM3tm *k*_CO_ values by 0.9089, this being the ratio of *k*_CO_ values calculated at the PM3tm and DFT levels of theory for **4** and **5**. That said, the peripheral inclusion of sterically obtrusive substituents in ligands often results in rather limited transmission of inductive electronic effects to the metal centre itself, as seen, for example, in experimental data for L = η^5^-C_5_H_5_ (*k*_CO_ = 15.24 Nm^−1^) and η^5^-C_5_Me_5_ (*k*_CO_ = 15.29 Nm^−1^). Similarly, experimental data are not available for toluidyne complexes of all ligands L, in which cases experimental data for the corresponding phenyl or xylyl carbynes are instead provided alongside those calculated for the toluidyne.

A bonus of the requisite frequency calculations is that the vibrational mode for the W≡C bond may be readily identified, though in contrast to similar essentially ‘pure’ vibrations for terminal oxo (M≡O) and toluidyne (M≡N) ligands, this is by necessity coupled to the vibration of the C–C bond connecting it to the aryl substituent. This mode appears within a remarkably narrow frequency range (1345–1356 cm^−1^), with the exception of **4** (1334 cm^−1^), perhaps also reflecting the electron-releasing nature of the HB(ImMe)_3_ ligand. The intensity of this mode, however, varies substantially, such that in some cases it is unlikely to be unambiguously identified in experimental IR spectra. This invariance in the value of ν_WC_ is also reflected in the derived Löwden bond orders (Table 2) for this bond, which fall within the very narrow range of 2.32–2.41. This is despite considerable variation in the calculated natural charge on tungsten (+0.405 to +1.177), while that for carbon is *comparatively* invariant (–0.105 to –0.299); i.e., electroneutrality would appear to balance charge distribution within the ‘LW’ unit so as to not significantly transmit this influence to the carbyne ligand.

Table 1 presents ν_CO_ frequencies corrected by an anharmonic scaling factor (λ_1_) of 0.9740 as implemented in the *SPARTAN20*^®^ software for the ωB97X-D/6-31G* combination [101,102], which, however, still overestimates these frequencies relative to those observed experimentally. Calculated vibrational frequencies generally exceed experimentally determined values due to incomplete incorporation of electron correlation, neglect of mechanical anharmonicity and the use of finite basis sets [103,104,105].

This overestimation is assumed to be relatively uniform, allowing for the development of generic scaling factors (λ) derived via least-squares analysis of calculated vs. experimental frequencies for various test sets of molecules. Such test sets typically involve small molecules comprising first and second row elements but rarely metals. Moreover, single scaling factors are not universally appropriate for the entire vibrational spectroscopy range (400–4000 cm^−1^) [106], and the fundamental modes from which they are derived generally fall below the range of interest to organometallic chemists (1800–2200 cm^−1^). For the present discussion, it therefore seems appropriate to consider an alternative scaling factor (λ_2_ = 0.9297), which we have derived from consideration of 18 experimental and fundamental modes from Table 2, with the caveat that only data measured in dichloromethane solutions were used, discarding those from solid-state or alkane solution measurements. Gas phase data were calculated, since there seemed little benefit in introducing further artificial approximations such as conductor-like polarizable continuum, molecular electron density (SMD) or conductor-like screening models (COSMO) [107,108,109,110,111] when the aim was to construct an approximate but internally consistent steric–electronic map rather than to seek out absolute values.

The data points may be loosely grouped according to the charge on the complex, with the general observation that as this increased from anionic through neutral to cationic, so too did the *k*_CO_ value. It should, however, be noted that these groupings are not well separated. Rather, some cationic complexes are coordinated by strong net σ-donors, e.g., *N,N′,N″-*trimethyltriazacyclononane (Me_3_[9]aneN_3_, Entry **7**) and tris(dimethylpyrazolyl)methane (HC(pzMe_2_)_3_, Entry **17**), such that comparatively low values are observed for *ν*_CO_ and *k*_CO_. Likewise, the icosohedral dicarbollide complexes [W(≡CC_6_H_4_Me-4)(CO)_2_(η^5^-C_2_B_9_H_9_R_2_)]^−^ (R = H, Me), whilst anionic, have frequencies not dissimilar to those of neutral **4** (Entry **1**) and **5** (Entry **2**), while the anionic docosohedral example [W(≡CC_6_H_4_Me-4)(CO)_2_(η^6^-C_2_B_10_H_10_Me_2_)]^−^ has a considerably higher *k*_CO_ value 15.04 Ncm^−1^. There is no correlation obvious to us between the net charge on the complex and derived WC bond orders or W≡C bond lengths for the carbyne ligand.

### 2.4. Sub-Series of Ligands

Table 1 and Table 2 along with Figure 3 and Figure 4 contain a number of as yet hypothetical derivatives that have yet to be prepared but which would appear to be entirely plausible based on the demonstrated viability of the ligands L in other systems. Some comments on sub-classes now follow.

#### 2.4.1. Hydrotris(N-R^1^-imidazolylidenyl)borates

Central to this communication are the tris(imidazolylidene)borates HB(ImR^1^)_3_. From Figure 3, it is clear that the ligand HB(ImMe)_3_ occupies a position in a somewhat sparsely populated area of the electronic–steric map, being both strongly basic and also imparting considerable steric prophylaxis upon the carbonyl and carbyne co-ligands akin to that provided by the popular HB(pzMe_2_)_3_ ligand. The experimental and calculated values for *k*_CO_ are comparable to those for Stone’s dicarbollide complexes (L = η^5^-C_2_B_9_H_9_R_2_ R = H, Me) [79,88] which, however, carry a net negative charge, and so it must be assumed much of the negative charge resides within the carbaborane cage.

**Figure 5:**
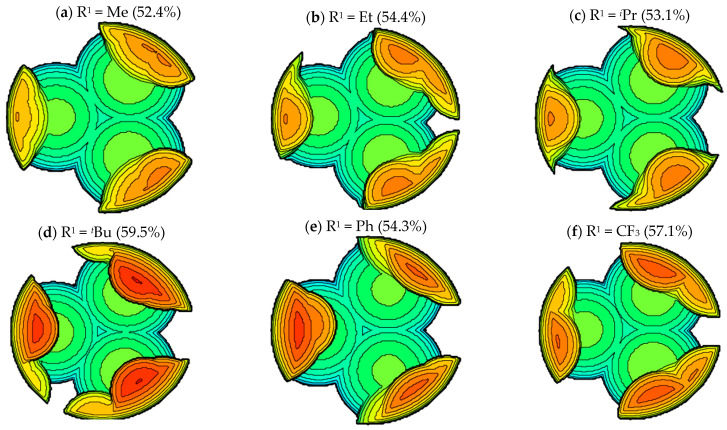
Steric maps [92,93] and %*V*_bur_ values in parentheses for a series of hydrotris(*N*-R^1^-imidazolylidenyl)borates HB(ImR^1^)_3_ where (**a**) R^1^ = Me; (**b**) R^1^ = Et; (**c**) R^1^ = *^i^*Pr; (**d**) R^1^ = *^t^*Bu; (**e**) R^1^ = Ph; (**f**) R^1^ = CF_3_.

As expected, the %*V*_bur_ value for **4** is close to that of **5**. Smith has developed synthetic routes to the pro-ligand salts that carry *N*-substituents of varying bulk (*^t^*Bu, Cy, C_6_H_2_Me_3_-2,4,6) [4] and accordingly entries **1** (R^1^ = Me, **4**), **35** (R^1^ = Et), **36** (R^1^ = *^i^*Pr), **37** (R^1^ = ^t^Bu) and **38** (R^1^ = Ph) survey the sequential inclusion of increasing steric bulk at the position β to the metal. All attempts to geometrically minimize, or indeed even reasonably construct, the derivative with R^1^ = mesityl met with spectacular failure, perhaps indicating a step too far, though this ligand has been successfully installed on four-coordinate nickel [37]. The phenyl derivative **38**, however, is able to accommodate unsubstituted aryl groups by allowing them to interdigitate between the carbonyl and carbyne ligands such that the aryl planes are near colinear with the W^…^B vector. A very approximate value for the %*V*_bur_ of 56.6% is provided by the hypothetical and implausible (distorted) octahedral complex [WMe_3_{HB(ImMes)_3_] (PM3tm level of theory). While it is not dissimilar to the value (59.8%) estimated for L = neutral MeC(CH_2_PPh_2_)_3_ (**16**) and anionic MeB(CH_2_PPh_2_)_3_ (**31**), inclusion of this excessive steric bulk would seem problematic. It should, however, be noted that a rich organometallic chemistry has emerged for the dihydro*bis*(*N*-mesitylimidazolylidenyl)borate ligand coordinated to tantalum [33,34], for which the bidentate variant presents a considerably reduced steric impact, e.g., *V*_bur_ = 39.8% in pseudo*-*octahedral [TaMe_4_{H_2_B(ImMes)_2_}]. The trifluoromethylimidazolylidenyl derivate (Entry **39**) was also considered and found to be a rather modest net donor (ν_CO_ = 15.2 Ncm^−1^) while presenting a comparatively occlusive encapsulating pocket (*V*_bur_ = 57.1%). The only currently available synthesis of *N*-trifluoromethylimidazole [111] is, however, not particularly amenable to the scales needed for an exploration of the HB(ImCF_3_)_3_ ligand. Figure 5 depicts the steric maps that arise from %*V*_bur_ calculations and shows the progression in steric encumbrance as the *N*-substituents are replaced along the alkyl series R^1^ = Me, Et, *^i^*Pr, *^t^*Bu alongside those for R = Ph and CF_3_.

**Figure 6:**
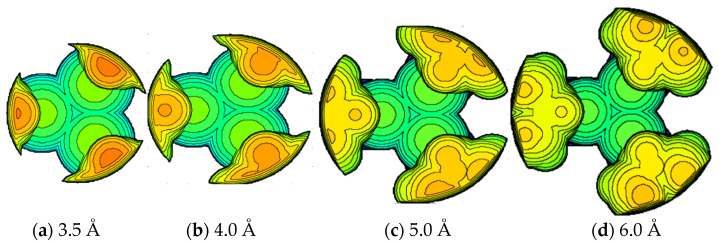
Steric map dependence on radius of coordination sphere employed.

What is immediately apparent from Figure 4 is that replacement of the ‘parent’ *N*-methylimidazole, which is both commercially available and cheap, with ethyl, iso-propyl or phenyl imidazoles actually results in very modest variation in the steric impact around the coordination sphere of the metal because the groups can direct their bulk away from the carbonyl and carbyne ligands. It is only with the *^t^*Bu (and to a lesser extent the CF_3_) derivative that this bulk is unavoidably directed towards the metals centre. This is clear when the 3.5 Å value typically and arbitrarily employed in %*V*_bur_ calculations is replaced by 4.0, 5.0 and 6.0 Å (Figure 6), respectively. Thus, inclusion of phenyl, primary or secondary alkyl groups appears to have rather a modest steric influence directly on the metal coordination sphere but may contribute in a secondary manner to compound longevity by reducing the collisional cross section (Arrhenius pre-exponential factor) for proceeding reactions. It seems that only with tertiary alkyl (e.g., *^t^*Bu) or *ortho*-substituted aryl substituents (e.g., mesityl) that a significant impact on the steric profile is likely to manifest in the reactivity.

An intriguing question does, however, arise when the steric bulk is exaggerated, in that whilst this might be expected to increase the donor strength of the NHC:→W interaction, the inter-ligand repulsion is such that there is a notable increase in the W–C bond lengths of both the NHC donors cis (mean value) and trans to the carbyne (Table 3).

Thus, the simple σ-basicity vs. π-acidity of the free NHC is only part of the story if the metal–donor bond length increases (weakens?) significantly. This does not appear to be the case in the present system, in that while the *^t^*Bu derivative has especially long NHC–W bond lengths, it is nevertheless the most potent net donor (*k*_CO_ = 14.41 Ncm^−1^) of all the ligands considered. In the case of the complexes [Ni(NO)(L)] where L represents a sub-set of ligands considered in Table 1 and Table 2 (η^5^-C_5_Me_5_, Tp*, Hb(mt*^t^*^Bu^)_3_ and PhB(CH_2_PPh_2_)_3_ [112,113,114,115,116]) alongside those for selected tris(imidazolylidenyl)borates RB(ImR^1^)_3_ (R = H, Ph; R^1^ = Me, *^t^*Bu, Mesilyl, CH_2_Cy [37]), Smith employed nitrosyl stretching frequency as a measure of the relative donor ability of ‘L’. Similar σ-donor/π-acceptor arguments apply as they do to CO with the caveat that depending on the electronic nature of the metal centre, the nitrosyl may bend; i.e., lower values for ν_NO_ may indicate an electron rich metal centre *or* bending, which becomes more prevalent for late-transition metal centres with high *d*-occupancies [117]. In the case of four-coordinate nitrosyls of nickel, the situation is complicated by subtleties in the electronic nature of the nickel that remain moot [47,49]. While Smith was consistent in reporting data from the same essentially non-coordinating solvent toluene (or sometimes THF), data from other sources were acquired from a variety of media (not always stated) including the solid state (KBr, Nujol, Ar_(s)_, etc.). The selenoimidazolylborate is a case in point for which the reported solid-state IR spectrum comprised two ν_NO_ bands [114]. Since the crystal structure revealed a single crystallographically independent molecule, one might assume the second vibrational mode was due to an alternative crystal modification in the bulk sample. Given the two bands differ by 11 cm^−1^ and the entire Tolman ν^T^ scale only spans 45 cm^−1^, the importance of using solution derived data, preferably from a common solvent, is demonstrated.

#### 2.4.2. Cyclopentadienyl Derivatives

In terms of percentage buried volume, the cyclopentadienyl ligand is somewhat unassuming (*V*_bur_ = 35.2%), and this is most commonly ‘bulked out’ via permethylation (**12**: L = C_5_Me_5_ *V*_bur_ = 42.4%), inclusion of trimethylsilyl substituents (**26**: L = C_5_H_3_(SiMe_3_)_2_ *V*_bur_ = 54.5%) or benzannulation with either one (**22**: L = indenyl *V*_bur_ = 37.3%) or two (**23**: L = fluorenyl *V*_bur_ = 40.4%) benzo rings. This imbues variable electron-releasing nature in the series C_5_H_3_(SiMe_3_)_2_ > C_5_Me_5_ > C_5_H_5_ ≈ fluorenyl > indenyl. A subtlety emerges from the geometry minimization of the indenyl derivative, which reveals a structural basis for Basolo’s ‘indenyl effect’ [61,118,119]. Incipient ring slippage (η^5^→η^3^) might be inferred, given that the angle between the tungsten, the cyclopentadienyl ring centroid and the unique carbon atom is slightly acute (84.8°), such that the unique carbon (2.319 Å) and adjacent *pseudo* η^3^-carbons (2.355, 2.352 Å) are noticeably closer to tungsten than are the benzo-fused carbons (mean: 2.534 Å). The C_6_H_4_ unit makes an angle of *ca* 5.9° with the three non-ring-fused carbons of the cyclopentadienyl ring. This slippage places the benzenoid ring trans to the carbyne ligand, as might be expected based on the characteristic trans influence of carbyne ligands. Experimentally acquired structural data are not currently available for indenyl, fluorenyl or bis(trimethylsilyl)cyclopentadienyl ligated carbynes through which to further explore this question, though enhanced reactivity in associative ligand addition reactions has been noted for the indenyl carbyne [Mo(≡CC_6_H_3_Me_2_-2,6)(CO)_2_(η^5^-C_9_H_7_)] [61].

Although no examples exist of carbyne complexes bearing by the perchlorocyclopentadienyl ligand (**25**: L = η^5^-C_5_Cl_5_), the tricarbido bimetallic complex [ReMn(μ_2_-C_3_)(CO)_2_(NO)(PPh_3_)(η^5^-C_5_H_5_)(η^5^-C_5_Cl_5_)]^+^ described by Gladysz [120,121] might be viewed as possessing a degree of manganese carbyne character. Perchlorination results in a modest increase in the steric bulk of the ligand (40.8 cf. 42.4% for η-C_5_Me_5_) but a quite substantial decrease in donor ability (*k*_CO_ = 15.51 Ncm^−1^). Perphenylation, in contrast, has only a modest effect on the net basicity of the ligand (*k*_CO_ = 15.34 Ncm^−1^), while the buried volume increases significantly (*V*_bur_ = 48.3%) due to the requisite orientation of the aryl groups to near orthogonal to the cyclopentadienyl plane. The tetraphenylcyclopentadienyl carbyne complex [W(≡CPh)(PPh_2_C_6_H_4_CH=CHPh)(η^5^-C_5_HPh_4_)] [122] and a single rather exotic pentaphenylcyclopentadienyl complex [W(≡CPh)(NCMe)(η^2^-C_60_)(η^5^-C_5_Ph_5_)] [123] have been described.

#### 2.4.3. Arene Derivatives

While hexahapto arene co-ligated carbyne complexes such as **18**, **20** and **21** appear unknown, a manifold of intriguing molybdenum carbyne complexes bearing the C_6_H_4_(C_6_H_4_P*^i^*Pr_2_-2)_2_-1,4 trans-coordinating diphosphine have been shown by Agapie to enter into variable degrees of arene-molybdenum interaction during transformations that demonstrate the interplay of carbyne and carbido ligands [124,125,126,127]. It therefore seems reasonable to anticipate that compounds akin to Entries **18**, **20** and **21** will emerge. It is apparent that conclusions similar to those for cyclopentadienyl substituents will result, except that the overall complex bears a positive charge, providing a point of connection with group 7 carbynes [M(≡CR)(CO)_2_(η^5^-C_5_H_5_)]^+^ (M = Mn, Re) [128,129,130,131,132]. The hexaethylbenzene derivative **21** would appear to present a sterically quite encapsulating environment (*V*_bur_ = 53.3%) cf. the hexamethyl analogue (*V*_bur_ = 45.9%) due to the 3-up/3-down mutual disposition of the ethyl substituents. This feature has been employed to favour unusual regiochemistry in selective alkane binding by the ‘W(CO)_2_(η^6^-C_6_Et_6_)’ fragment [133]. Finally, we note that the inorganic benzene B_3_N_3_Me_3_ has, as expected, a steric profile similar (*V*_bur_ = 46.2%) to that of C_6_Me_6_ (*V*_bur_ = 45.9%), and the non-planar ring is a comparable net donor to the tungsten centre (*k*_CO_ = 15.84 Ncm^−1^ vs. 15.85 Ncm^−1^ for **20**). This is also implicit from infrared data for [Cr(CO)_3_(η^6^-B_3_N_3_Me_6_)] (Cyclohexane: ν_CO_ = 1963, 1867 cm^−1^) vs. [Cr(CO)_3_(η^6^-C_6_Me_6_)] (ν_CO_ = 1962, 1888 cm^−1^) provided in a publication in which Werner indicated that [W(CO)_3_(η^6^-B_3_N_3_Me_6_)] also appeared viable [134,135].

#### 2.4.4. Pnictolyl Ligands

Schrock has explored the utility of high oxidation state carbene and carbyne complexes ligated by σ- and η^5^-pyrollyl ligands [136], though low oxidation variants have yet to emerge. Carbynes ligated by the heavier pnictolyl ligands η^5^-AC_4_R_4_ (A = P, As), however, remain unknown, though both ligands have been shown to serve as ersatz cyclopentadienyls [137,138,139,140]. With the ready availability of synthetic routes to anionic pnictolyl reagents, it may be presumed that complexes of the form [M(≡CR)(CO)_2_(η^5^-AC_4_R’_4_)] (A = P, As, Sb) will emerge in the future, given that, like carbynes, arsolyl ligands have been shown to support intermetallic bonding [141,142,143].

#### 2.4.5. Toluidyne Orientation

**Figure 7:**
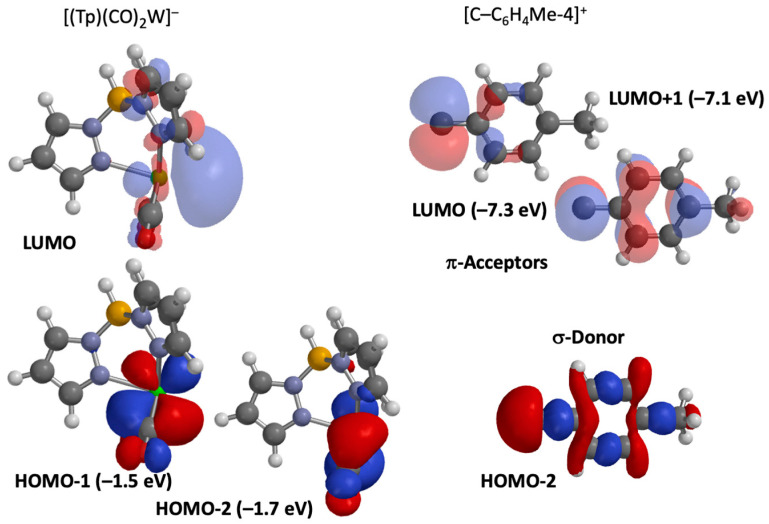
Valence orbitals of the hypothetical [CC_6_H_4_Me-4]^+^ and *d^6^*-[W(CO)_2_(Tp)] fragments.

Perusal of the structures, experimentally or computationally derived, reveals a broad range of orientations of the toluidine ring with respect to the nominal coordination axes. This is of secondary importance in that for all examples, the ^1^H NMR spectra involve a simple AA’BB’ pattern indicating free rotation on the ^1^H NMR (and ^13^C) NMR timescale(s). Arbitrarily adopting the cationic carbyne formalism ([CF]^+^, [NO]^+^ and CO being isoelectronic molecules), coordinated to a *d^6^*-ML_5_ fragment, the two carbyne acceptor orbitals vary in energy by only 0.2 eV, as do the two metal retrodative orbitals (HOMO-1, HOMO-2) of, e.g., the ‘W(CO)_2_(Tp)’ fragment (Figure 7). The HOMO itself is invariably associated with metal–carbonyl π-bonding and is orthogonal (δ-symmetry) to the W–Carbyne vector. Accordingly, any conformational preference should be presumed to reflect inter-ligand steric factors and/or intermolecular packing effects. For the majority of structurally characterized carbyne complexes of the M(CO)_2_(Tp*) fragment; for example, the carbyne substituent typically nestles between two dimethylpyrazolyl groups. NB: The molecular orbitals of the actual carbyne complex are, as they must be, independent of the arbitrary electron allocation to hypothetical constituent fragments; i.e., similar interpretation based on [CC_6_H_4_Me]^3–^ and *d^2^*-ML_5_^3+^ or neutral CC_6_H_4_Me-4 and *d^5^*-ML_5_ deconstructions lead to the same conclusion.

### 2.5. A Heterobimetallic Hydrotris(imidazolylidenyl)borate Complex

To date, the tris(imidazolylidenyl)borate class of ligands has only been employed in monometallic systems; however, terminal carbyne ligands have an extensively documented propensity to support metal–metal bond formation, as championed by Stone [144]. In particular, the addition of gold(I) reagents to monometallic carbyne complexes [145,146,147,148,149,150,151,152,153,154,155] is of interest due to the tendency of the carbyne to adopt a semi-bridging rather than the more common symmetrical bridging geometry. This is considered to arise when the carbyne bridges electronically disparate metals, and therefore, the late high *d*-occupancy metal (*d^10^* gold(I) or platinum(0)) is considered to act as a σ-donor (*Z*-type metal–ligand bonding [156]) to the carbyne carbon. Accordingly, the reaction of **4** with [AuCl(SMe_2_)] was investigated and found to readily provide the bimetallic complex [WAu(μ-CC_6_H_4_Me-4)Cl(CO)_2_{HB(ImMe)_3_}] (**6**, Scheme 4). The complex is somewhat unstable in solution, slowly depositing elemental gold during unsuccessful attempts to slowly obtain crystallographically serviceable crystals. The formulation, however, rests reliably on spectroscopic data which may be compared with precedents for other carbyne and tungsten substituents. The reaction is accompanied by a shift in the ν_CO_ absorptions to a higher frequency (CH_2_Cl_2_: 1971, 1879 cm^−1^) than those of the precursor in the same solvent (1958, 1873 cm^−1^). The carbyne carbon resonance in the ^13^C{^1^H} NMR spectrum appears at δ_C_ = 277.7, and while this is only marginally shifted from that of the precursor (280.7 ppm), there is a dramatic decrease in the value of ^1^*J*_WC_ (85 Hz cf. 171.3 Hz for **4**), which is consistent with the increase coordination number (reduced *s*-character) of both tungsten and carbon. The resonances due to the imidazolylidene donors appear at 187.7 [^1^*J*_CW_ = 90 Hz], 173.7 [^1^*J*_CW_ = 71 Hz] in a similar region to the precursor but with more similar values for ^1^*J*_WC_ (90, 71 Hz) once the trans influence of the carbyne is alleviated upon gold adduct formation.

**Figure 8:**
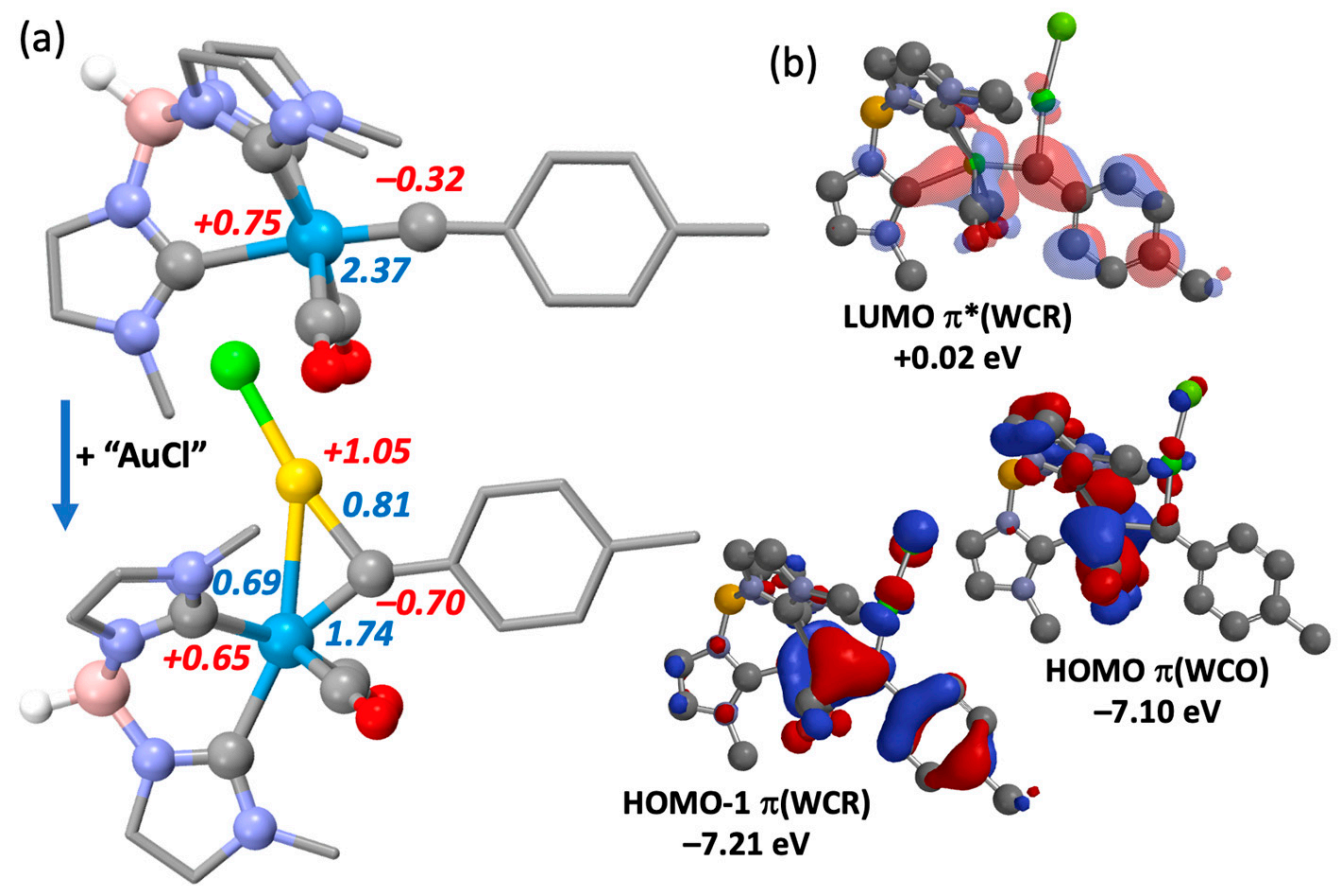
(**a**) Optimized geometries of [W(≡CC_6_H_4_Me-4)(CO)_2_{HB(ImMe)_3_}] (**4**) and the heterobimetallic complex [WAu(μ-CC_6_H_4_Me-4)Cl(CO)_2_{HB(ImMe)_3_}] (**6**) indicating changes in Löwdin bond order (blue) and natural atomic charge (red) upon ‘AuCl’ adduct formation (ωB97X-D/6-31G*/LANL2Dζ, hydrogen atoms omitted, tolyl and imidazolyl groups simplified). (**b**) Frontier molecular orbitals of interest for **6** at Isovalue = 0.032 √(e/au^2^).

While the ^1^H and ^13^C{^1^H} NMR spectra each confirm a locally *C*_s_ symmetric environment around the tungsten, at least on these timescales, they do not distinguish between the AuCl unit lying syn or anti to the imidazolylidene units; however, based on precedent from the sterically similar HB(pzMe_2_)_3_ ligand, it seems likely that the AuCl unit nestles between two imidazolylidene rings. This geometry was adequately modelled (Figure 8) at the ωB97X-D/6-31G/LANL2Dζ/gas-phase level of DFT, from which it would appear that the W–C bond clearly retains its considerable multiple-bond character (W–C = 1.913 Å). The W–C–C (148.9°) and Au–C–C (121.5°) angles indicate semi rather than symmetrical bridging such that the C–C and W–Au vectors form an obtuse angle of 101.4°. Despite numerous (>80) examples of structurally authenticated W–Au bonds, only two have bonds that are not supported by bridging ligands, viz. the compounds [WAu(CO)_3_(PPh_3_)(η^5^-C_5_H_4_R)] (R = H 2.698 Å [157] and CH_2_CH_2_NHMe_2_^+^Cl^−^ 2.712 Å [158]). The optimized Au–W bond length for **6** (2.812 Å) is therefore comparable to these, though towards the longer end of the range. The infrared ν_CO_ absorptions are noted at 1955 and 1899 cm^−1^ (λ_2_), while TD-DFT analysis suggests that the colour of the complex may be attributed to absorptions calculated at 420 nm (W–C ≈ *z*-axis: HOMO-LUMO; *d*_xy_-W=Cπ*), 357 (HOMO-LUMO+1; *d*_xy_-WAuσ*) and 344 nm (HOMO-1-LUMO; W=Cπ-W=Cπ*), the first two of which involve considerable charge transfer.

## 3. Experimental

### 3.1. General Considerations

Experimental work was performed using standard Schlenk techniques with pure dry nitrogen or argon, or in an argon atmosphere glovebox, unless otherwise specified. All solvents used in the syntheses were dried and degassed. Unless otherwise indicated, reagents were used as received from commercial suppliers.

Infrared data were obtained using a Shimadzu FTIR-8400 for solutions and a Perkin Elmer FTIR Spectrum 2 for solid-state ATR measurements. NMR spectra were measured using Bruker Avance 400, Bruker Avance 600 or Bruker Avance 800 spectrometers at the temperatures indicated. Chemical shifts (δ) are reported in ppm with coupling constants in Hz, all referenced to the appropriate solvent resonance. Multiplicities indicated do not include the satellites for the ^183^W isotopomers, the couplings for which are listed separately. Positive ion high-resolution electrospray ionisation mass spectroscopy (ESI) data were provided by the ANU Research School of Chemistry Joint Mass Spectrometry team; an acetonitrile matrix was used for all samples. Single-crystal X-ray diffraction (XRD) crystallographic data were acquired with a SuperNova CCD diffractometer using Mo-Kα radiation (λ = 0.71073 Å), employing CrysAlis PRO software [159] (https://www.agilent.com/ accessed on 20 November 2023), refined with Olex2 [160], and structural models were depicted using Mercury [161]. Elemental microanalytical data were not acquired [162].

Computational studies were performed using the *SPARTAN20* suite of programs [40]. Cyclic voltammetry (CV) was performed using a PalmSens 4 Potentiostat/Galvanostat/Impedance Analyser and carried out in a single-compartment 3-electrode glass cell, with a 3 mm glassy carbon working electrode, platinum wire counter electrode and silver wire pseudo-reference electrode. Analyte solutions were prepared at 1 mM in dichloromethane with 0.1 M [NBu_4_][PF_6_] supporting electrolyte. Solutions were sparged with N_2_ bubbled through dichloromethane prior to measurements, then maintained under an atmosphere of N_2_ during voltammetry. All measurements were referenced to ferrocene, which was added to the solution following each measurement.

Infrared and NMR spectra for all new compounds are provided in the accompanying Supplementary Materials.

### 3.2. Synthesis of [W(≡CC_6_H_4_Me-4)(CO)_2_(pic)_2_(Br)] (***2a***)

Note: the following synthesis is a modified version of the synthesis of cis,cis,trans*-*[W(≡CC_6_H_3_Me_2_-2,6)(CO)_2_(pic)_2_Br] [15]. A solution of 4-bromotoluene (6.568 g, 38.40 mmol) in diethyl ether (60 mL) was cooled to 0 °C before lithium (0.618 g, 89.0 mmol, hammered and cut wire) was added. This was stirred vigorously at 0 °C for 30 min before being allowed to slowly warm to room temperature and being stirred for a further 3 h. The lithium reagent was added dropwise to a suspension of [W(CO)_6_] (8.445 g, 24.00 mmol) in diethyl ether (60 mL) until IR spectroscopy indicated no [W(CO)_6_] remained. The red solution was cooled to –78 °C before trifluoroacetic anhydride (3.40 mL, 24.3 mmol) was added dropwise over a period of 10 min, resulting in a yellow precipitate. After stirring for 30 min at –78 °C, 4-picoline (6.0 mL, 62 mmol) was added. The suspension was allowed to slowly warm to room temperature and stirred overnight. The yellow precipitate was isolated via cannula filtration and extracted with dichloromethane (60 mL) and the combined extracts were filtered through diatomaceous earth, followed by washing with further dichloromethane until the extracts were colourless (total volume 200 mL). The solvent volume was reduced to *ca* 40 mL under reduced pressure before slow dilution with hexane (300 mL) to precipitate a yellow-orange solid that was freed of supernatant via cannula filtration. Hexane (80 mL) was added, and the suspension was ultrasonically triturated for 10 min to remove residual [W(CO)_6_]. The yellow-orange solid was collected on a sinter, washed with further hexane (20 mL) and dried under high vacuum (13.094 g, 21.446 mmol, 89% isolated yield).

**Figure 9:**
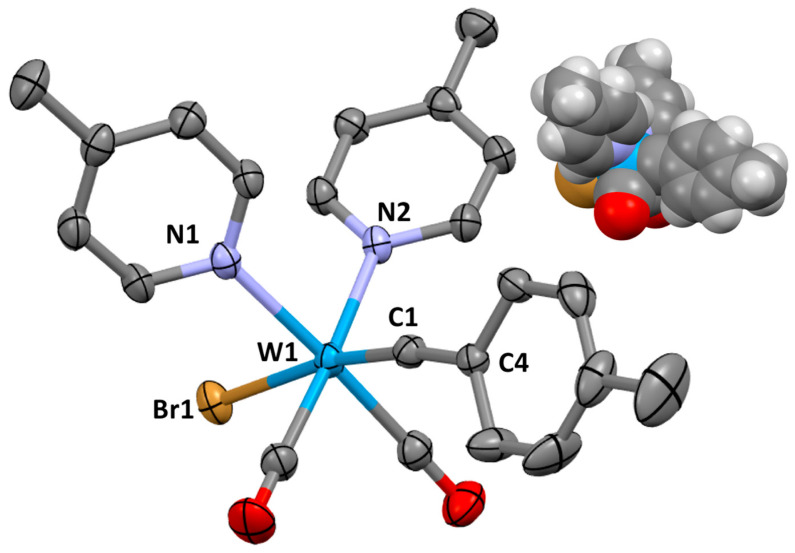
Molecular structure of cis,cis,trans*-*[W(≡CC_6_H_4_Me-4)(CO)_2_(pic)_2_(Br)] in a crystal (50% displacement ellipsoids, hydrogen atoms omitted for clarity).

**IR** (CH_2_Cl_2_, cm^−1^): 1986 vs. ν_CO_, 1897 vs. ν_CO_. **IR** (ATR, cm^−1^): 1970 vs. ν_CO_, 1881 vs. ν_CO_.**^1^H NMR** (600 MHz, CD_2_Cl_2_, 298 K) δ_H_ = 8.91 [d, 4 H, ^3^*J*_HH_ = 7, H^2,6^(pic)], 7.25 [d, 2 H, ^3^*J*_HH_ = 8, H^2,6^(C_6_H_4_)], 7.13 [d, 4 H, ^3^*J*_HH_ = 7, H^3,5^(pic)], 7.09 [d, 2 H, ^3^*J*_HH_ = 9, H^3,5^(C_6_H_4_)], 2.38 [s, 6 H, pic-CH_3_], 2.29 [s, 3 H, tolyl-CH_3_]. **^13^C{^1^H} NMR** (151 MHz, CD_2_Cl_2_, 298 K) δ_C_ = 263.9 [^1^*J*_WC_ = 203 Hz, W≡C], 221.4 [^1^*J*_WC_ = 169 Hz, CO], 153.3 [C^2,6^(pic)], 151.0 [C^4^(pic)], 146.9 [C^1^(C_6_H_4_)], 138.5 [C^4^(C_6_H_4_)], 129.4 [C^2,6^(C_6_H_4_)], 129.1 [C^3,5^(C_6_H_4_)], 126.3 [C^3,5^(pic)], 21.8 [tolyl-*C*H_3_], 21.3 [pic-*C*H_3_]. **MS** (ESI, +ve ion, *m*/*z*): Found: 609.0375. Calcd for C_22_H_22_N_2_O_2_^79^Br^184^W [M + H]^+^: 609.0374. Crystals suitable for structural determination were grown from liquid diffusion of diethyl ether into a saturated dichloromethane solution of the sample at -20 °C. **Crystal Data** for C_22_H_21_BrN_2_O_2_W.(OEt_2_)_0.5_ (*M*_w_= 646.23 gmol^−1^): monoclinic, space group *C2*/c (no. 15), *a* = 23.3477(7) Å, *b* = 12.5106(2) Å, *c* = 17.9409(5) Å, *β* = 110.628(3) °, *V* = 4904.4(2) Å^3^, *Z* = 8, *T* = 150.0(1) K, μ(Mo *K*α) = 6.364 mm^−1^, *D*_calc_ = 1.750 Mgm^−3^, 37431 reflections measured (7.422° ≤ 2Θ ≤ 64.280°), 8075 unique which were used in all calculations. The final *R*_1_ was 0.0311 (*I* > 2σ(*I*)) and *wR*_2_ was 0.0671 (all data) with 291 refined parameters with one restraint, CCDC 2305468. The molecular geometry in the solid state is depicted in Figure 9.

### 3.3. Synthesis of [Mo(≡CC_6_H_4_Me-4)(CO)_2_(pic)_2_(Br)] (***2b***)

A solution of 4-bromotoluene (6.842 g, 40.00 mmol) in diethylether (50 mL) was cooled to 0 °C before lithium (1.3 g, 190 mmol, hammered and cut wire) was added. This was stirred vigorously at 0 °C for 30 min before being allowed to slowly warm to room temperature and being stirred for a further 3.5 h. The lithium reagent was added dropwise to a suspension of [Mo(CO)_6_] (6.338 g, 24.01 mmol) in diethyl ether (60 mL) until negligible [Mo(CO)_6_] remained, as indicated by in situ IR spectroscopy. The red solution was cooled to –78 °C before trifluoroacetic anhydride (3.40 mL, 24.3 mmol) was added dropwise over a period of 10 min. After being stirred for 45 min at –78 °C, 4-picoline (6.0 mL, 62 mmol) was added. The suspension was allowed to slowly warm to room temperature and stirred overnight. The yellow precipitate was isolated via cannula filtration and extracted with dichloromethane (50 mL) and the extracts filtered through diatomaceous earth, followed by washing with further dichloromethane (6 × 5 mL). The volume was reduced to 50 mL under reduced pressure before slow dilution with hexane (120 mL) to precipitate a yellow solid that was freed of supernatant via cannula filtration and dried under high vacuum (8.473 g, 16.25 mmol, 68% isolated yield).

**IR** (CH_2_Cl_2_, cm^−1^): 2000 vs. ν_CO_, 1918 vs. ν_CO_. **IR** (ATR, cm^−1^): 1986 vs. ν_CO_, 1913 vs. ν_CO_. **^1^H NMR** (600 MHz, CD_2_Cl_2_, 298 K) δ_H_ = 8.87 [d, 4 H, ^3^*J*_HH_ = 6, H^2,6^(pic)], 7.36 [d, 2 H, ^3^*J*_HH_ = 8, H^2,6^(C_6_H_4_)], 7.08-7.14 [m, 6 H, H^3,5^(pic) and H^3,5^(C_6_H_4_) over-lapped], 2.37 [s, 6 H, pic-CH_3_], 2.32 [s, 3 H, tolyl-CH_3_]. **^13^C{^1^H} NMR** (151 MHz, CH_2_Cl_2_, 298 K) δ_C_ = 276.8 [Mo≡C], 224.4 [CO], 152.9 [C^2,6^(pic)], 150.6 [C^4^(pic)], 143.5 [C^1^(C_6_H_4_)], 139.5 [C^4^(C_6_H_4_)], 129.3 [C^3,5^(C_6_H_4_)], 129.2 [C^2,6^(C_6_H_4_)], 125.9 [C^3,5^(pic)], 21.8 [tolyl-CH_3_], 21.26 [pic-CH_3_)]. **MS** (ESI, +ve ion, *m/z*): Found: 443.0659. Calcd for C_22_H_22_N_2_O_2_^98^Mo [M – Br]^+^: 443.0662.

### 3.4. Synthesis of [Tris(1-methylimidazolium)borate] Bis(hexafluorophosphate) ([1](PF_6_)_2_)

A 1 L three-necked flask was fitted with a stirrer bar, water-cooled reflux condenser, pressure-equalizing dropping funnel and a gas outlet leading to a NaOH scrubber. The entire apparatus was flushed with nitrogen for 30 min before trimethylamine-borane complex (7.32 g, 100 mmol) was added, followed by 150 mL degassed chlorobenzene. To the dropping was added 50 mL chlorobenzene and bromine (7.8 mL, 85 mmol Br_2_). The bromine solution was added to the flask at a rate of about one drop/second whilst the reaction was flushed with a gentle stream of nitrogen. This reaction is initially very exothermic and the rate of bromine addition should be adjusted accordingly; caution should also be exercised, since hydrogen gas is also liberated at this stage. After approximately half of the bromine had been added, the exothermicity was less pronounced and rate of addition of the remainder could be increased safely. The mixture was then stirred for 3 h at ambient temperature, during which time the orange colour of bromine slowly faded to a pale yellow. Hydrogen bromide was liberated during this time as nitrogen was continuously swept over the reaction. *N*-methylimidazole (28 mL, 330 mmol) was added to the mixture, and the apparatus was carefully transferred to a heating mantle, where it was brought to reflux for 4-6 h. Upon heating, a white crystalline solid precipitated from the reaction mixture; extended heating is to be discouraged, as this leads to the formation of tarry yellow materials and poor yields of product. The chlorobenzene layer was decanted from the solids while warm, and the flask was then rinsed with 3 × 100 mL portions of toluene; the washings were subsequently discarded. The white solid was dissolved into 100 mL methanol and added slowly to a vigorously stirred solution of NaPF_6_ (35 g, 210 mmol; NaBF_4_ may also be used) in 100 mL methanol, from which the product precipitated as a fluffy white solid. The white solids were collected via filtration, washed with 3 × 50 mL portions each of methanol and Et_2_O and dried under suction. Purity was sufficient for synthetic purposes, though an analytically pure sample was obtained via re-crystallisation from acetone/Et_2_O (vapour diffusion). Isolated yield 11.50 g (21 mmol, 21%) as the PF_6_ salt or 12.90 g (30 mmol, 30%) as the BF_4_ salt.

**IR** (THF, cm^−1^): 2454 w ν_BH_. **IR** (ATR, cm^−1^): 2455 vs. ν_BH_, 827 vs. ν_PF_. ^1^H NMR (400 MHz, CD_3_CN, 25 °C): δ_H_ = 8.17 (s, 3 H, N_2_CH), 7.38 (t, ^3^*J*_HH_ = 1.7 Hz, 3 H, NCHCH), 7.17 (t, ^3^*J*_HH_ = 1.7 Hz, 3 H, NCHCH), 3.87, (s.br, 1 H, BH), 2.18 (s, 9 H, NCH_3_). **^13^C{^1^H} NMR** (101 MHz, CD_3_CN, 25 °C): δ_C_= 139.9 (N_2_C), 125.6 (NCC), 124.2 (NCC), 36.4 (NCH_3_). ^11^B{^1^H} NMR (128 MHz, CD_3_CN, 25 °C): δ_B_ = −3.50 (BH). **^11^B NMR** (128 MHz, CD_3_CN, 25 °C): δ_B_ = −3.42 (d, ^1^*J*_BH_ = 121.7 Hz, BH). ^19^F NMR (376 MHz, CD_3_CN, 25 °C): δF = −72.9 (d, ^1^*J*_PF_ = 708 Hz, PF_6_). **^31^P{^1^H} NMR** (162 MHz, CD_3_CN, 25 °C): δ_P_ = −144.6 (sept, ^1^*J*_PF_ = 700 Hz, PF_6_). **MS** (ESI, +ve ion, *m*/*z*): Found: 257.1685. Calcd for C_12_H_18_N_6_^11^B. [M – H]^+^: 257.1686. **Crystal Data** for C_12_H_19_BF_12_N_6_P_2_ (*M*_w_ =548.08 gmol^−1^): monoclinic, space group *P2*_1_/n (no. 14), *a* = 20.7403(2) Å, *b* = 10.10590(10) Å, *c* = 20.7879(2) Å, *β* = 97.4530(10)°, *V* = 4320.32(7) Å^3^, *Z* = 8, *T* = 150.2(1) K, μ(CuKα) = 2.945 mm^−1^, *D*_calc_ = 1.685 Mgm^−3^, 54277 reflections measured (5.664° ≤ 2Θ ≤ 156.216°), 9112 unique (*R*_int_ = 0.0625, *R*_sigma_ = 0.0400), which were used in all calculations. The final *R*_1_ was 0.0603 (*I* > 2σ(*I*)) and *wR*_2_ was 0.1725 (all data) for 711 refined parameters with 296 restraints. CCDC 2305467.

### 3.5. Synthesis of [W(≡CC_6_H_4_Me-4)(CO)_2_{HB(ImMe)_3_}] (***4***)

Tris(1-methylimidazolium)borate bis(hexafluorophosphate) ([1](PF_6_)_2_: 0.400 g, 0.730 mmol) was dissolved in tetrahydrofuran (30 mL) and cooled (dry ice/propanone). A solution of *n*-butyllithium (1.40 mL, 1.6 M, 2.20 mmol, hexanes) was added dropwise at –78 °C. While being stirred for 90 min at this temperature, the solution became pale yellow, at which point solid [W(≡CC_6_H_4_Me-4)(CO)_2_(γ-pic)_2_(Br)] (**2a**: 0.45 g, 0.70 mmol) was added. After it was stirred at this temperature for 15 min, the mixture was allowed to warm to room temperature and stirred for a further 3 h and then freed of volatiles under reduced pressure. The residual black tar was dissolved in a minimum of dichloromethane (~5mL) and subjected to flash column chromatography (silica gel, N_2_, CH_2_Cl_2_). The orange band that eluted first was collected, and the solvent was removed under reduced pressure to give (**4**) a bright orange powder. Yield: 0.11 g (0.18 mmol, 20%).

**IR** (CH_2_Cl_2_, cm^−1^): 2453 w ν_BH_, 1958 vs. ν_CO_, 1873 vs. ν_CO_. **IR** (ATR, cm^−1^): 2442 w ν_BH_, 1949 vs. ν_CO_, 1867 vs. ν_CO_. **^1^H NMR** (800 MHz, CD_2_Cl_2_, 25 °C): δ_H_ = 7.36 [d, ^3^*J*_HH_ = 7.4 Hz, 2 H, H^2,6^(C_6_H_4_)], 7.11 [d, ^3^*J*_HH_ = 1.4 Hz, 2 H, NCHCH], 7.08 (d, ^3^*J*_HH_ = 1.5 Hz, 1 H, NCCH], 7.07 [d, ^3^*J*_HH_ = 7.0 Hz, 2 H, H^3,5^(C_6_H_4_)], 6.84 [d, ^3^*J*_HH_ = 1.4 Hz, 2 H, NCCH], 6.76 [d, ^3^*J*_HH_ = 1.3 Hz, 1 H, NCCH], 3.80 [s, 3 H, NCH_3_), 3.79 [s, 6 H, NCH_3_], 2.26 [s, 3 H, CCH_3_]. **^13^C{^1^H} NMR** (201 MHz, CD_2_Cl_2_, 25 °C): δ_C_ = 280.7 [^1^*J*_CW_ = 171.3 Hz, W≡C], 223.3 [^1^*J*_CW_ = 132.1 Hz, CO], 192.5 [^1^*J*_CW_ = 95.0 Hz, NCN)], 181.7 [^1^*J*_CW_ = 44.7 Hz, NCN], 151.5 [^2^*J*_CW_ = 39.1 Hz, C^4^(C_6_H_4_)], 136.9 [C^2,6^(C_6_H_4_)], 129.0 [C^3,5^(C_6_H_4_)], 128.8 [C^4^(C_6_H_4_)], 124.3 [C^5^(C_3_N_2_H_2_)], 123.7 [C^5^(C_3_N_2_H_2_), 120.7 [C^4^(C_3_N_2_H_2_)], 120.3 [C^4^(C_3_N_2_H_2_)], 38.8 [NCH_3_], 38.1 [NCH_3_], 21.8 [CCH_3_]. **^11^B{^1^H} NMR** (128 MHz, CDCl_3_, 25 °C): δ_B_ = −1.41 (BH). **^11^B NMR** (128 MHz, CDCl_3_, 25 °C): δ_B_ = −1.35 (d, ^1^*J*_BH_ = 97.9 Hz, BH). **MS** (ESI, +ve ion, *m*/*z*): Found: 599.1572. Calcd for C_22_H_24_^11^BN_6_O_2_^184^W. [M + H]^+^: 599.1563. **CV** (CH_2_Cl_2_): *E*_½_ = 0.00 V vs. [Fe(C_5_H_5_)_2_]/[Fe(C_5_H_5_)_2_]^+^. See Figure 8 for computationally optimized molecular structure.

### 3.6. Synthesis of [W(≡CC_6_H_4_Me-4)(CO)_2_(Tp*)] (***5***)

The complex has been previously described via the reaction of the thermolabile intermediate [W(≡CC_6_H_4_Me-4)Br(CO)_4_] (from [W{=C(OMe)C_6_H_4_Me-4}(CO)_5_] and BBr_3_) and K[Tp*] (80%) [17]. The present synthesis follows a similar approach to the synthesis of [W(≡CC_6_H_3_Me_2_-2,6)(CO)_2_(Tp)] [17]. Sodium hydrotris(3,5-dimethyl-1-pyrazolyl) borate (0.183 g, 0.544 mmol) was dissolved in dichloromethane (15 mL) and added to a solution of [W(≡CC_6_H_4_Me-4)(CO)_2_(γ-pic)_2_(Br)] (**2a**: 0.307 g, 0.506 mmol) in dichloromethane (20 mL) with stirring overnight. The solution slowly darkened from pale orange to dark red, and this transition was visible after 3 h. Solvent and picoline were removed under reduced pressure, and the residue was redissolved in a minimum of dichloromethane (~2mL) and purified via flash column chromatography using a 1:2 DCM to petroleum spirits 60-80 eluent (silica gel, N_2_). The orange fraction which eluted first was collected, and solvent was removed under reduced pressure to give (**5**) a bright orange powder. Yield: 217 mg (0.339 mmol, 67%). **IR** (CH_2_Cl_2_, cm^−1^): 2554w ν_BH_, 1971 vs. ν_CO_, 1879 vs. ν_CO_. **IR** (ATR, cm^−1^): 2550 w ν_BH_, 1954 vs. ν_CO_, 1861 vs. ν_CO_. **^1^H NMR** (800 MHz, CDCl_3_, 25°C): δ_H_ = 7.36 [d, ^3^*J*_HH_ = 7.4 Hz, 2 H, H^2,6^(C_6_H_4_)], 7.10 [d, ^3^*J*_HH_ = 7.9 Hz, 2 H, H^3,5^(C_6_H_4_)], 5.89 [s, 2 H, H^4^(pz)], 5.79 [s, 1 H, H^4^(pz)], 2.52 [s, 6 H, pzCH_3_], 2.47 [s, 3 H, pzCH_3_], 2.38 [s, 6 H, pzCH_3_], 2.35 [s, 3 H, pzCH_3_], 2.31 [s, 3 H, C_6_H_4_C*H_3_*). **^13^C{^1^H} NMR** (201 MHz, CDCl_3_, 25°C): δ_C_ = 279.2 [^1^*J*_CW_ = 186.3 Hz, W≡C], 224.0 [^1^*J*_CW_ = 166.5 Hz, CO], 152.4 [C^5^(pz)], 152.1 [C^5^(pz)], 148.0 [^2^*J*_CW_ = 42.5 Hz, C^1^(C_6_H_4_)], 145.7 [C^3^(pz)], 144.5 [C^3^(pz)], 137.8 [C^2,6^(C_6_H_4_)], 129.3 [C^3,5^(C_6_H_4_)], 128.8 [C^4^(C_6_H_4_)], 106.7 [C^4^(pz)], 106.5 [C^4^(pz)], 21.8 [C_6_H_4_*C*H_3_], 16.7 [pzCH_3_], 15.4 [pzCH_3_], 12.8 [pzCH_3_], 12.8 [pzCH_3_)]. **^11^B{^1^H} NMR** (128 MHz, CDCl_3_, 25 °C): δ_B_ = −9.17 (BH). **^11^B NMR** (128 MHz, CDCl_3_, 25 °C): δ_B_ = −9.15 (br, BH). **MS** (ESI, +ve ion, *m/z*): Found: 641.2039. Calcd for C_25_H_30_^11^BN_6_O_2_^184^W 641.2033. [M + H]^+^. **CV** (CH_2_Cl_2_): *E*_½_ = 0.18 V vs. [Fe(C_5_H_5_)_2_]/[Fe(C_5_H_5_)_2_]^+^.

### 3.7. Synthesis of [WAu(μ_2_-CC_6_H_4_Me-4)Cl(CO)_2_{HB(ImMe)_3_}] (***6***)

To a solution of [W(≡CC_6_H_4_Me-4)(CO)_2_{HB(ImMe)_3_}] (**4**: 20 mg, 0.033 mmol) in dichloromethane (5 mL) was added [AuCl(SMe_2_)] (10 mg, 0.034 mmol) with stirring, whereupon the solution turned from bright orange to dark red. After 30 min, a further equivalent of [AuCl(SMe_2_)] (10 mg, 0.034 mmol) was added to the reaction, which was stirred for a further 15 min (longer reaction times resulted in gold mirror formation). After this time, the resulting solution was subjected to flash column chromatography (diatomaceous earth, CH_2_Cl_2_, N_2_) to collect the bright orange fraction, from which solvent was removed under reduced pressure. The resulting dark orange powder was suspended in n-hexane (10 mL) and then collected via vacuum filtration, washed with n-hexane (3 × 5 mL) and dried in vacuo for 45 min, to give a brown-gold powder of (**6**). Yield: 14 mg (8.7 μmol, 54%). **IR** (CH_2_Cl_2_, cm^−1^): 2455 w ν_BH_, 1986 vs. ν_CO_, 1911 vs. ν_CO_. **IR** (ATR, cm^−1^): 2454 w ν_BH_, 1983 vs. ν_CO_, 1879 vs. ν_CO_.**^1^H NMR** (600 MHz, CD_2_Cl_2_, 25 °C): δ_H_ = 7.87 [d, ^3^*J*_HH_ = 8.1 Hz, 2 H, H^2,6^(C_6_H_4_)]), 7.24 [d, ^3^*J*_HH_ = 7.8 Hz, 2 H, H^3,5^(C_6_H_4_)], 7.20 [d, ^3^*J*_HH_ = 1.6 Hz, 2 H, NCCH], 7.14 [d, ^3^*J*_HH_ = 1.6 Hz, 1 H, NCCH], 6.91 [d, ^3^*J*_HH_ = 1.6 Hz, 2 H, NCCH], 6.90 [d, ^3^*J*_HH_ = 1.6 Hz, 1 H, NCCH), 3.95 [s, 3 H, NCH_3_], 3.72 [s, 6 H, NCH_3_), 2.36[s, 3 H, CCH_3_]. **^13^C{^1^H} NMR** (151 MHz, CD_2_Cl_2_, 25°C): δ_C_ = 277.7 [^1^*J*_CW_ = 85 Hz, W≡C], 216.0 [^1^*J*_CW_ = 119 Hz, CO], 187.7 [^1^*J*_CW_ = 90 Hz, NCN], 173.7 [^1^*J*_CW_ = 71 Hz, NCN], 149.6 [C^2,6^(C_6_H_4_)], 140.4 [^2^*J*_CW_ = 97 Hz, C^1^(C_6_H_4_)], 130.3 [C^3,5^(C_6_H_4_)], 129.5 [C^4^(C_6_H_4_)], 124.8 [C^3^(C_3_N_2_H_2_)], 124.5 [C^3^(C_3_N_2_H_2_)], 122.0 [C^4^(C_3_N_2_H_2_)], 121.5 [C^4^(C_3_N_2_H_2_)], 39.4 [NCH_3_], 38.0 [NCH_3_], 21.81 [C_6_H_4_*C*H_3_]. **^11^B{^1^H} NMR** (128 MHz, CD_2_Cl_2_, 25 °C): δ_B_ = –1.60 (BH). **^11^B NMR** (128 MHz, CD_2_Cl_2_, 25°C): δ_B_ = –1.47 [d, ^1^*J*_BH_ = 101.2 Hz, BH]. **MS** (ESI, +ve ion, *m/z*): Found: 853.0721. Calcd for C_22_H_23_Au^11^B^35^ClN_6_O_2_^184^W 853.0731. [M + Na]^+^. See Figure 8 for computationally optimized molecular geometry.

## 4. Conclusions

The first examples of mononuclear and binuclear carbyne complexes ligated by poly(imidazolylidenyl)borates have been isolated. Spectroscopic data for these add to the growing evidence that poly(imidazolylidenyl)borates are particularly strong net donor ligands. These data are contextualised by comparison with those having a wide range of more familiar κ^3^, η^5^ and η^6^ facially capping ligands, with recourse to two parameters *k*_CO_ and %*Vol*_bur_. Reminiscent of the steric/electronic map presented by Tolman to describe the coordinative features of phosphines, a similar map based on *k*_CO_ and %*Vol*_bur_ suggests that HB(Im^R^)_3_ ligands occupy a sparsely populated region of ligand space, associated with potent net basicity and significant (but variable) steric encumbrance.

The first of these parameters, *k*_CO_ (a Cotton–Kraihanzel force constant), is given by

*k*_CO_ [Ncm^−1^] = 1.7426 × 10^−6^ Ncm × (ν_s_^2^ + ν_as_^2^)

where ν_s_ and ν_as_ are the uncorrected frequencies (in cm^−1^) calculated at the ωB97X-D/6-31G*/LANL2Dζ level of theory for the complexes [W(≡CC_6_H_4_Me-4)(CO)_2_(L)] in the gas phase.

The second of these, %*Vol*_bur_, is obtained using the *SambVca* protocol [35] applied to either the computationally optimised geometries or, where available, the experimentally determined atomic coordinates with hydrogen atoms included based on a sphere of 3.5Å radius centred on tungsten. Because this approach may be applied to hypothetical as well as real molecules, the method may enjoy predictive value with limited computational expense. For comparison of calculated and experimentally determined infrared data in the region of ν_CO_-associated vibrations (1850–2100 cm^−1^), an anharmonic scaling factor of 0.9297 is recommended for the combination of the ωB97X-D functional and 6-31G* basis set.

## Figures and Tables

**Table 1:** Experimental *^a^* and calculated *^b^* infra-red and steric *^c^* properties of [W(≡CC_6_H_4_Me)(CO)_2_(L)]^x+^.

	L	x	ν_(CO)_/cm^−1^	*k*_CK_/Ncm^–1 *d*^	ν_(CO)_/cm^−1^	*k*_CK_/Ncm^−1^	ν_(WC)_/cm^−1^	*%*Vol_bur_*^c^*	Ref
			Experimental *^a^*		Calculated *^b^*	λ_1_(λ_2_) *^i^*			
**1**	**κ** **^3^** **-HB(ImMe)_3_**	**0**	**1958, 1873**	**14.80**	**1969, 1907**	**15.15 (14.76)**	**1334**	**52.4**	**-**
**2**	**κ** **^3^** **-HB(pzMe_2_)_3_ *^g^***	**0**	**1971, 1889 *^c^***	**15.07**	**1980, 1912**	**15.27 (14.86)**	**1350**	**50.7**	[64]
**3**	η^5^-C_2_B_9_H_9_Me_2_	1–	1956, 1874	14.82	1970, 1900	15.10 (14.71)	1354	49.6	[79]
**4**	κ^3^-CpCo(PO_3_Me_2_)_3_	0	1961, 1859	14.74	1980, 1906	15.23 (14.83)	1353	44.0	[68]
**5**	κ^3^-HB(mt)_3_	0	1967, 1875	14.91	1983, 1916	15.33 (14.93)	1352	48.7	[67]
**6**	η^5^-C_2_B_9_H_11_	1–	1965, 1880	14.93	1974, 1906	15.18 (14.77)	1356	44.3	[79]
**7**	κ^3^-Me3[9]aneN_3_ *^e^*	1+	1975, 1879 *^f^*	15.00	2003, 1940	15.68 (15.27)	1347	52.5	[80]
**8**	κ^3^-HC(py)_3_ *^e^*	1+	1988, 1894 *^b,f^*	15.22	2007, 1949	15.78 (15.37)	1346	46.2	[81]
**9**	κ^3^-[9]aneS3 *^e,h^*	1+	2007, 1925 *^f^*	15.59	2029, 1980	16.20 (15.78)	1346	46.0	[81]
**10**	η^5^-C_5_H_5_	0	1982, 1902	15.24	1997, 1941	15.64 (15.23)	1348	35.2	[82]
**11**	κ^3^-HB(pz)_3_ *^k^*	0	1986, 1903	15.28	1998, 1934	15.49 (15.11)	1347	43.3	[84]
**12**	η^5^-C_5_Me_5_	0	1981, 1910 *^c,j^*	15.29	1989, 1933	15.51 (15.12)	1349	42.4	[86]
**13**	κ^3^-HC(pz)_3_	1+	1995, 1912	15.42	2016, 1959	15.93 (15.52)	1347	41.7	[87]
**14**	η^6^-C_2_B_10_H_10_Me_2_	1–	1990, 1930	15.52	1981, 1932	15.44 (15.04)	1352	53.5	[89]
**15**	κ^3^-P(py)_3_ *^e^*	1+	2007, 1925 *^f^*	15.62	2008, 1951	15.80 (15.39)	1349	47.9	[81]
**16**	κ^3^-MeC(CH_2_Ph_2_)_3_ *^e,g^*	1+	1999, 1934 ^b,*f*^	15.62	*2095, 2037*	*17.01 (15.46)*	*n.r.*	*59.8*	[81]
**17**	κ^3^-HC(pzMe_2_)_3_	1+	–	–	2002, 1941	15.68 (15.27)	1349	49.1	–
**18**	κ^3^-MeC(CH_2_Pme_2_)_3_	1+	–	–	2021, 1974	16.09 (15.67)	1342	51.5	–
**19**	η^6^-C_6_H_6_	1+	–	–	2051, 2017	16.68 (16.25)	1356	39.3	–
**20**	η^6^-C_6_Me_6_	1+	–	–	2030, 1989	16.28 (15.85)	1351	45.9	–
**21**	η^6^-C_6_Et_6_	1+	–	–	2019, 1975	16.08 (15.66)	1351	53.3	
**22**	η^5^-C_9_H_7_ (indenyl)	0	–	*^h^*	2002, 1949	15.74 (15.32)	1348	37.3	[61]
**23**	η^5^-C_13_H_9_ (fluorenyl)	0	–	–	1999, 1941	15.65 (15.24)	1356	40.4	–
**24**	η^5^-C_5_Ph_5_ *^g^*	0	–	–	*2077, 2015*	*16.88 (15.34)*	*1532*	*48.3*	*–*
**25**	η^5^-C_5_Cl_5_	0	–	–	2012, 1962	15.92 (15.51)	1354	40.8	–
**26**	η^5^-C_5_H_3_(SiMe_3_)_2_-1,3	0	–	–	1987, 1931	15.48 (15.08)	1307	54.5	–
**27**	η^5^-C_5_Me_4_N	0	–	–	1992, 1937	15.56 (15.16)	1350	39.2	–
**28**	η^5^-C_5_Me_4_P	0	–	–	1990, 1937	15.55 (15.15)	1348	41.7	–
**29**	η^5^-C_5_Me_4_As	0	–	–	1989, 1936	15.53 (15.13)	1348	37.5	–
**30**	η^5^-C_5_H_5_BH	0	–	–	2006, 1953	15.80 (15.39)	1351	40.8	–
**31**	κ^3^-MeB(CH_2_PPh_2_)_3_ *^g^*	0	–	–	*2072*, *2003*	*16.74 (15.22)*	*1519*	*59.8*	–
**32**	κ^3^-MeB(CH_2_Pme_2_)_3_	0	–	–	1988, 1933	15.50 (15.10)	1339	51.2	–
**33**	κ^3^-MeB(CH_2_Sme)_3_	0	–	–	1996, 1935	15.58 (15.17)	1348	49.7	–
**34**	κ^3^-HB(mt^Se^)_3_	0	–	–	1981, 1915	15.31 (14.91)	1357	49.7	–
**35**	κ^3^-HB(ImEt)_3_	0	–	–	1968, 1906	15.13 (14.74)	1322	54.4	–
**36**	κ^3^-HB(Im^i^Pr)_3_	0	–	–	1970, 1907	15.16 (14.76)	1340	53.1	
**37**	κ^3^-HB(Im*^t^*Bu)_3_	0	–	–	1950, 1881	14.80 (14.41)	1334	59.5	–
**38**	κ^3^-HB(ImPh)_3_	0	–	–	1981, 1919	15.34 (14.94)	1329	54.3	–
**39**	κ^3^-HB(ImCF_3_)_3_	0	–	–	1999, 1947	15.70 (15.29)	1337	57.1	

*^a^* Unless otherwise indicated, data were determined from dichloromethane solutions. *^b^* DFT:ωB97X-D/6-31G*/LANL2Dζ(W)/Gas-phase, anharmonic scaling factor 0.9420. *^c^* Percentage buried volume calculated [92] for a 3.5 Å sphere centred on tungsten with H-atoms included. *^d^*Cotton–Kraihanzel force constant [90].^*e*^ Experimental data for benzylidyne. *^f^* KBr pellet. *^g^*Values in italics were determined at the reduced PM3tm level of theory. *^h^* [Mo(≡CC_6_H_3_Me_2_-2,6)(CO)_2_(η^5^-C_9_H_7_)] has ν_CO_ = 1998, 1925 cm^−1^ [61]. *^i^*λ_1_ = 0.9420, λ_2_ = 0.9297. *^j^*Measured in *n*-hexane. *^k^*The complex [W(≡CC_6_H_4_Me-4)(CO)_2_{B(pz)_4_}] has identical ν_CO_ values to those for [W(≡CC_6_H_4_Me-4)(CO)_2_{HB(pz)_3_}], i.e., replacing the remote B–H substituent with pz has negligible electronic impact. *n.r.* = not identified with confidence or heavily coupled with other oscillators.

**Table 2:** Calculated (TT-DFT) *^a^* electronic absorptions of interest, natural atomic charges (*Z*) and Löwdin bond orders (LBO) for selected complexes [W(≡CC_6_H_4_Me)(CO)_2_(L)]^x++^.

	L	x	λ_max_/nm	λ_max_/nm	*Z*(W)	*Z*(C)	LBO	*r*(W≡C)/Å
			*d*_xy_→π*_W≡C_	π_W≡C_→π*_W≡C_			(W≡C)	
**1**	**κ** **^3^** **-HB(ImMe)_3_ (4)**	**0**	**433**	**332**	**+0.748**	**–0.316**	**2.37**	**1.833**
**2**	**κ** **^3^** **-HB(pzMe_2_)_3_ (5)**	**0**	**406**	**316**	**+1.013**	**–0.268**	**2.40**	**1.811**
**3**	η^5^-C_2_B_9_H_9_Me_2_	1–	435	359	+0.831	–0.214	2.35	1.810
**4**	κ^3^-CpCo(PO_3_Me_2_)_3_	0	431	374	+1.177	–0.299	2.40	1.802
**5**	κ^3^-HB(mt)_3_	0	444	335	+0.685	–0.256	2.42	1.800
**6**	η^5^-C_2_B_9_H_11_	1–	428	358	+0.845	–0.230	2.36	1.810
**7**	κ^3^-Me_3_[9]aneN_3_ *^b^*	1+	400	377	+0.858	–0.188	2.39	1.812
**8**	κ^3^-HC(py)_3_^x^	1+	403 *^b^*	377 *^b^*	+0.909	–0.213	2.40	1.813
**9**	κ^3^-[9]aneS_3_	1+	377	330	+0.405	–0.131	2.35	1.818
**10**	η^5^-C_5_H_5_	0	420	319	+0.851	–0.270	2.40	1.815
**11**	κ^3^-HB(pz)_3_	0	412	313	+0.979	–0.253	2.42	1.810
**12**	η^5^-C_5_Me_5_	0	430	326	+0.870	–0.284	2.41	1.814
**13**	κ^3^-HC(pz)_3_ *^b^*	1+	405	337	+0.886	–0.190	2.41	1.811
**14**	η^6^-C_2_B_10_H_10_Me_2_	1–	417	372	+0.732	–0.171	2.34	1.811
**15**	κ^3^-P(py)_3_ *^b^*	1+	384	332	+0.911	–0.214	2.40	1.809
**17**	κ^3^-HC(pzMe_2_)_3_	1+	386	319	+0.920	–0.208	2.40	1.810
**18**	κ^3^-MeC(cH_2_PMe_2_)_3_	1+	390	335	+0.146	–0.130	2.33	1.830
**19**	η^6^-C_6_H_6_	1+	356	381	+0.697	–0.105	2.32	1.820
**20**	η^6^-C_6_Me_6_	1+	386	333	+0.754	–0.134	2.35	1.813
**21**	η^6^-C_6_Et_6_	1+	379	336	+0.759	–0.130	2.33	1.814
**22**	η^5^-C_9_H_7_ (indenyl)	0	415	354	+0.889	–0.269	2.45	1.802
**23**	η^5^-C_13_H_9_ (fluorenyl)	0	436	357	+0.909	–0.237	2.45	1.798
**25**	η^5^-C_5_Cl_5_	0	422	323	+0.851	–0.214	2.41	1.805
**26**	η^5^-C_5_H_3_(SiMe_3_)_2_-1,3	0	418	318	+0.849	–0.257	2.40	1.812
**27**	η^5^-C_4_Me_4_N	0	415	326	+0.939	–0.273	2.40	1.811
**28**	η^5^-C_4_Me_4_P	0	391	365	+0.755	–0.247	2.37	1.816
**29**	η^5^-C_4_Me_4_As	0	390	366	+0.740	–0.255	2.37	1.816
**30**	η^5^-C_5_H_4_BH	0	383	323	+0.769	–0.164	2.37	1.811
**32**	κ^3^-MeB(cH_2_PMe_2_)_3_	0	412	329	+0.274	–0.213	2.37	1.825
**33**	κ^3^-MeB(cH_2_SMe)_3_	0	422	323	+0.587	–0.224	2.40	1.809
**34**	κ^3^-HB(mt^Se^)_3_	0	443	338	+0.631	–0.263	2.42	1.800
**35**	κ^3^-HB(ImEt)_3_	0	424	329	+0.763	–0.324	2.35	1.835
**36**	κ^3^-HB(Im*^i^*Pr)_3_	0	437	335	+0.749	–0.318	2.38	1.830
**37**	κ^3^-HB(Im*^t^*Bu)_3_	0	423	322	+0.864	–0.294	2.33	1.820
**38**	κ^3^-HB(ImPh)_3_	0	449	335	+0.960	–0.300	2.37	1.828
**39**	κ^3^-HB(ImCF_3_)_3_	0	426	329	+0.689	–0.238	2.37	1.824

*^a^* TD-DFT:ωB97X-D/6-31G*/LANL2Dζ(W)/gas-phase. *^b^* π*_W≡C_ does not correspond to the LUMO due to low-lying ligand(L)-centred virtual orbitals.

**Table 3:** Steric Impact of *N*-substituents in the Complexes [W(≡CC_6_H_4_Me)(CO)_2_{HB(ImR)_3_}].

R	Mean W–C	Mean W–C_cis_	W–C_trans_	*TR ^a^*
	Å	Å	Å	
Me	2.262	2.226	2.335	1.049
CF_3_	2.276	2.232	2.365	1.060
Et	2.268	2.233	2.339	1.047
*^i^*Pr	2.268	2.232	2.341	1.049
Ph	2.277	2.237	2.357	1.054
*^t^*Bu	2.349	2.312	2.424	1.048

*^a^* (W–C_trans_)/(Mean W–C_cis_).

## Data Availability

The data presented in this study are available in the accompanying electronic supporting information.

## Supplementary Materials

The following supporting information can be downloaded at: https://www.mdpi.com/1420-3049/28/23/7761/s1, IR, ^1^H, ^13^C{^1^H} and ^11^B NMR spectra for new compounds.
