# Supplementary material for: Poly(imidazolyliden-yl)borato Complexes of Tungsten: Mapping Steric vs. Electronic Features of Facially Coordinating Ligands
Source: Molecules. 2023 Nov 24;28(23):7761. doi: 10.3390/molecules28237761 (PMC10798377; doi:10.3390/molecules28237761)

Supporting Information

Poly(imidazolyliden-yl)borato Complexes of Tungsten: Map-ping Steric vs. Electronic Features of Facially Coordinating Ligands

Callum M. Inglis, Richard A. Manzano, Ryan M. Kirk, Manab Sharma, Madeleine D. Stewart, Lachlan J. Watson and Anthony F. Hill *

The following supporting information comprise characterisational spectra for new compounds reported in the associated manuscript.

CCDC 2305467 and 2305468 contain the supplementary crystallographic data for this paper and are available free of charge from the Cambridge Crystallographic Data Centre.

^{1}H NMR Spectrum of [HB(ImMe)$_3$](PF$_6$)$_2$ ([1](PF$_6$)$_2$) (400 MHz, CD$_3$CN, 25°C, δ)

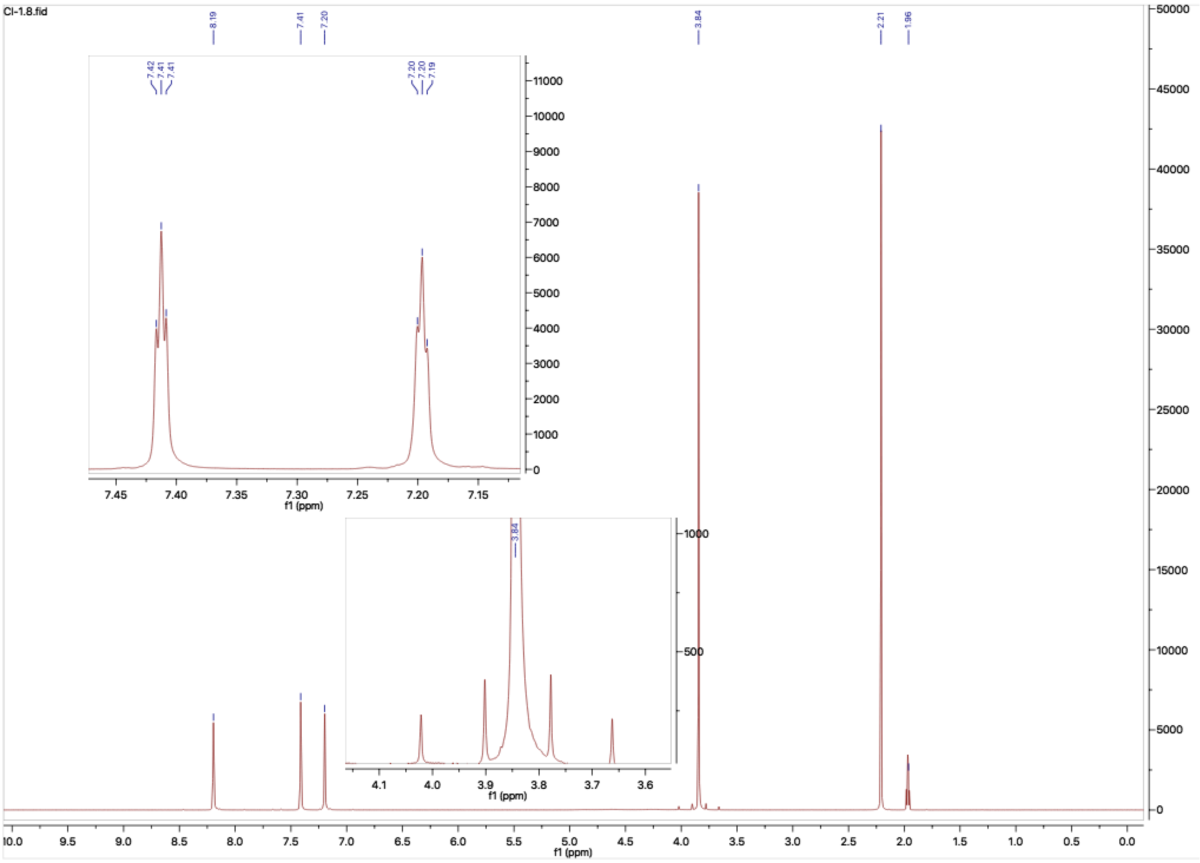

^{13}C{^{1}H} NMR spectrum of ([1](PF$_6$)$_2$) (101 MHz, CD$_3$CN, 25°C, δ)

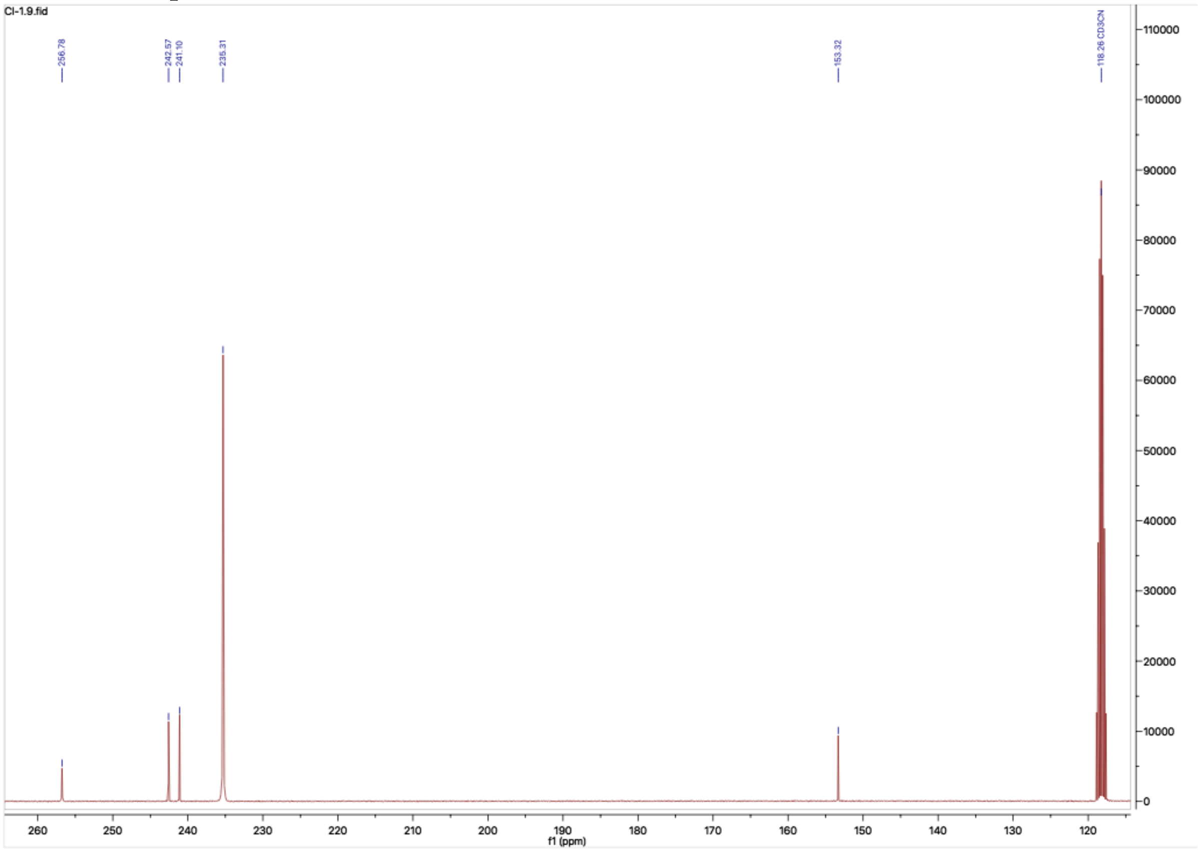

$^{11}B\{^1H\}$ NMR spectrum of ([1](PF_6)$_2$) (128.4 MHz, CD_3CN, 25°C, δ)

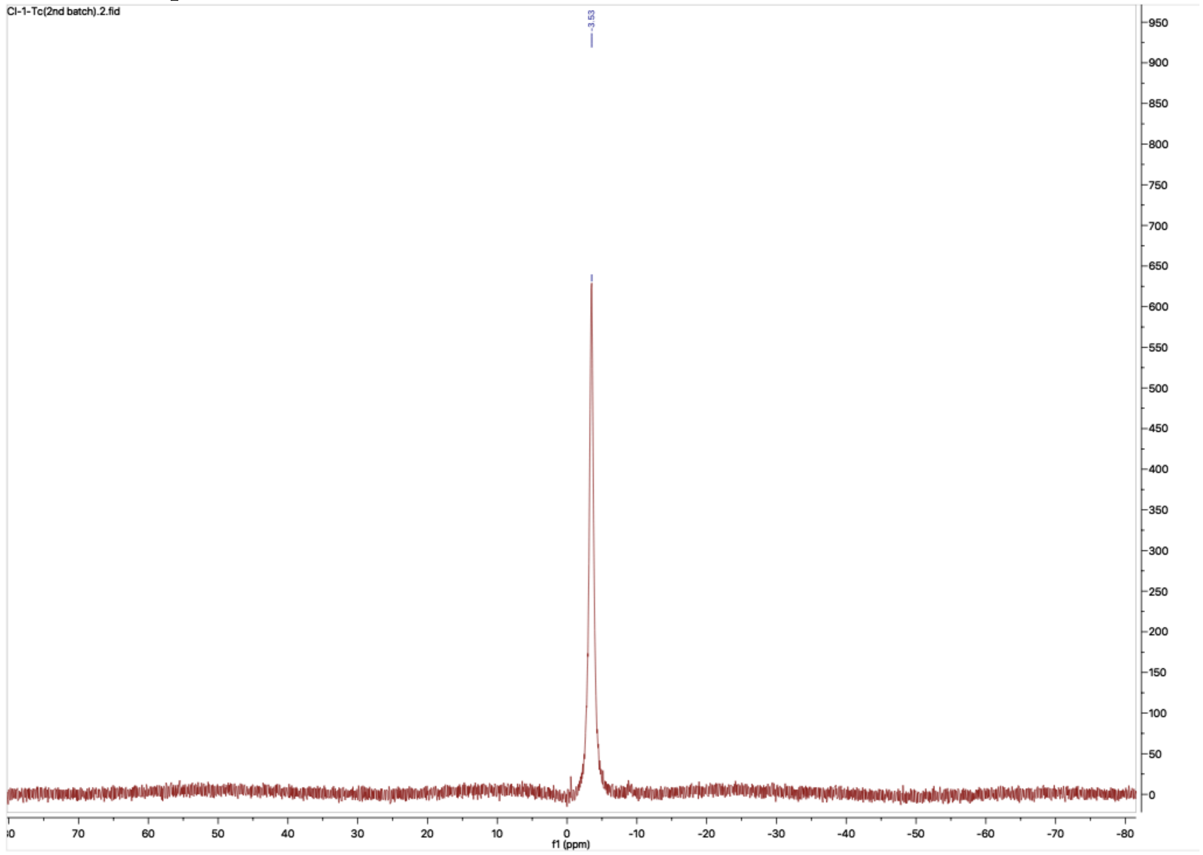

^{11}B NMR spectrum of ([1](PF_6)$_2$) (128.4 MHz, CD_3CN, 25°C, δ)

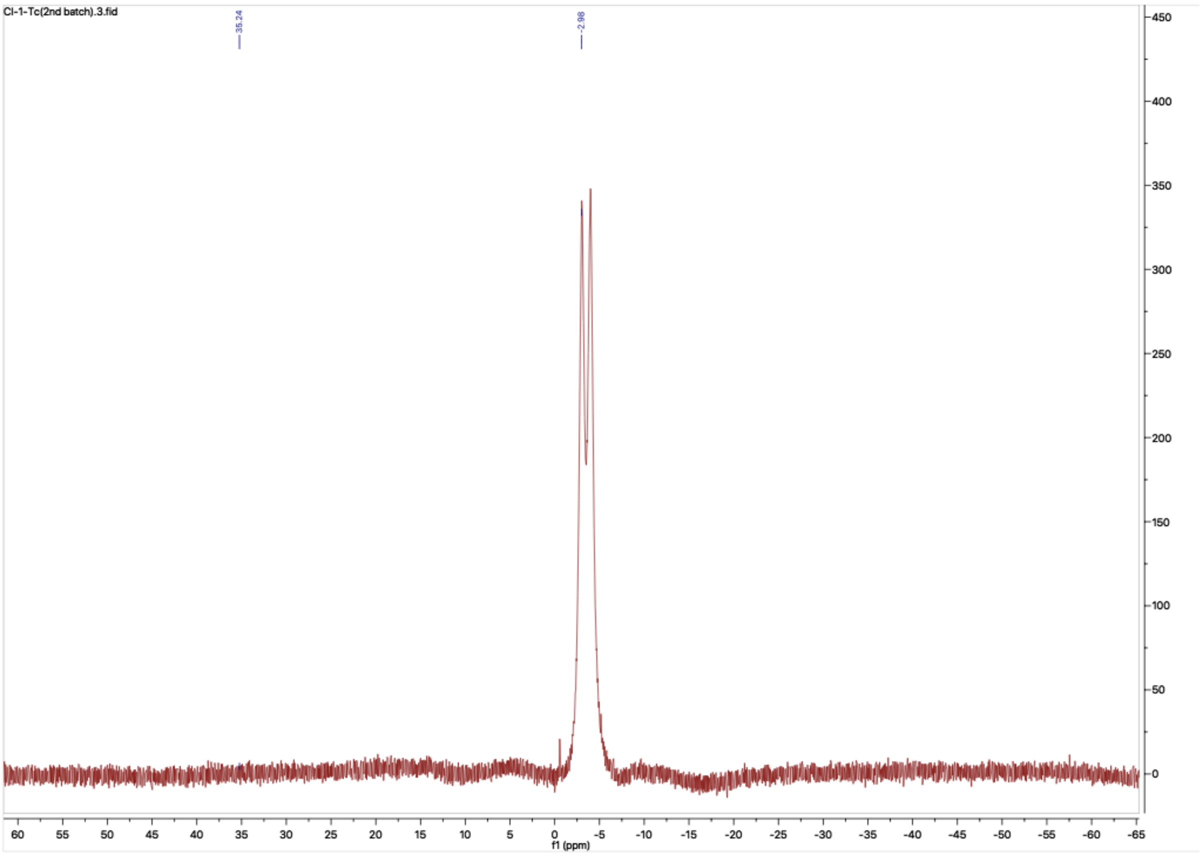

ATR Infrared Spectrum of [1](PF_6)$_2$ (Inset = CH_2Cl_2 solution spectrum, ν_{BH} region)

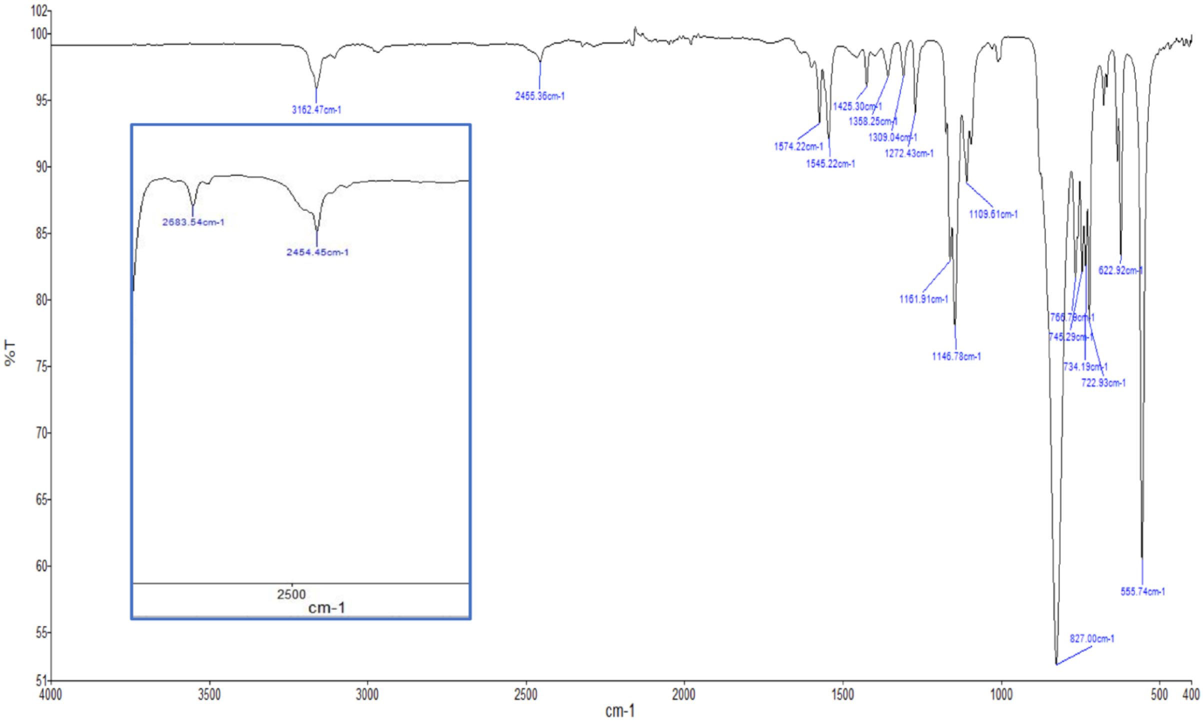

Mass Spectrometry for [1](PF_6)$_2$

Elemental Composition Report **Page 1**

Single Mass Analysis
Tolerance = 3.0 PPM / DBE: min = -1.5, max = 100.0
Element prediction: Off
Number of isotope peaks used for i-FIT = 3

Monoisotopic Mass, Odd and Even Electron Ions
90 formula(e) evaluated with 1 results within limits (up to 50 best isotopic matches for each mass)
Elements Used:
C: 0-50 H: 0-50 11B: 0-2 N: 0-6
CI-1/AJ SYNAPTG2-SI#NotSet 02-Aug-2022
67126 16:00:42
2925A 67 (0.149) Cm (43:169)
1: TOF MS ES+ 5.90e+007

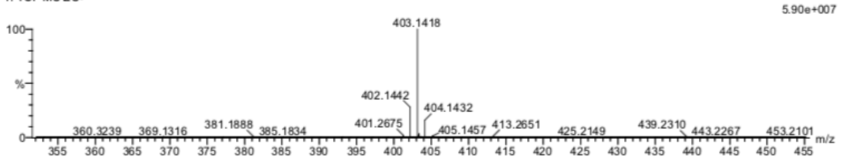

Minimum:				-1.5		
Maximum:		5.0	3.0	100.0		
Mass	Calc. Mass	mDa	PPM	DBE	i-FIT	Formula
403.1418	403.1407	1.1	2.7	23.5	4021.6	C29 H16 11B N2

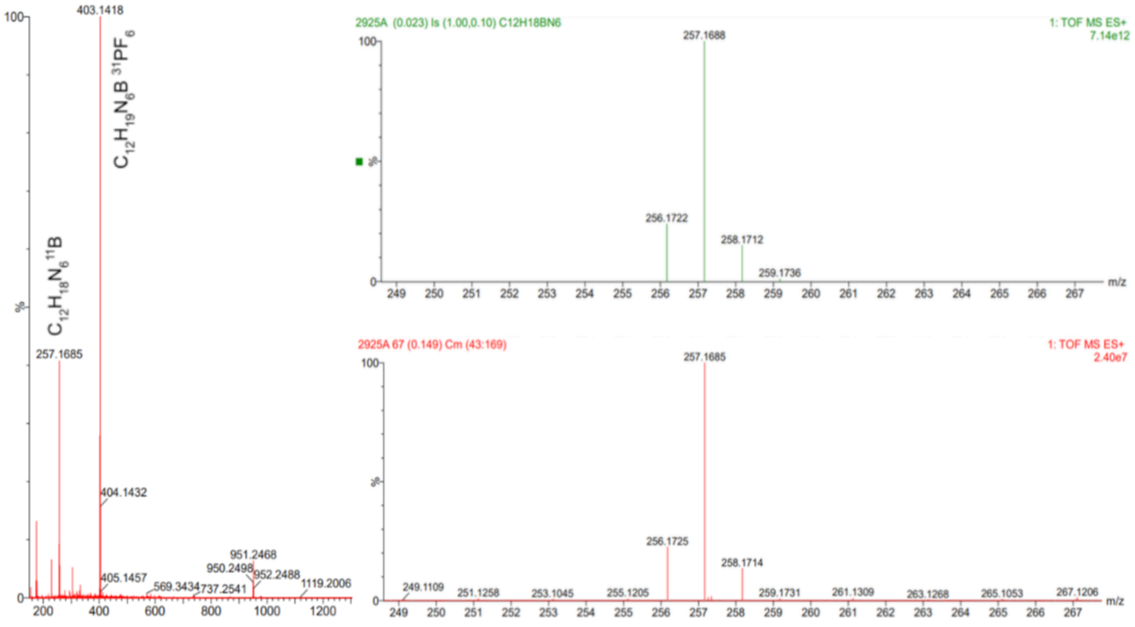

^{1}H NMR spectrum of [W(≡CC$_6$H$_4$Me-4)(CO)$_2$(pic)$_2$(Br)] (2a) (600 MHz, CD$_2$Cl$_2$, 298 K)

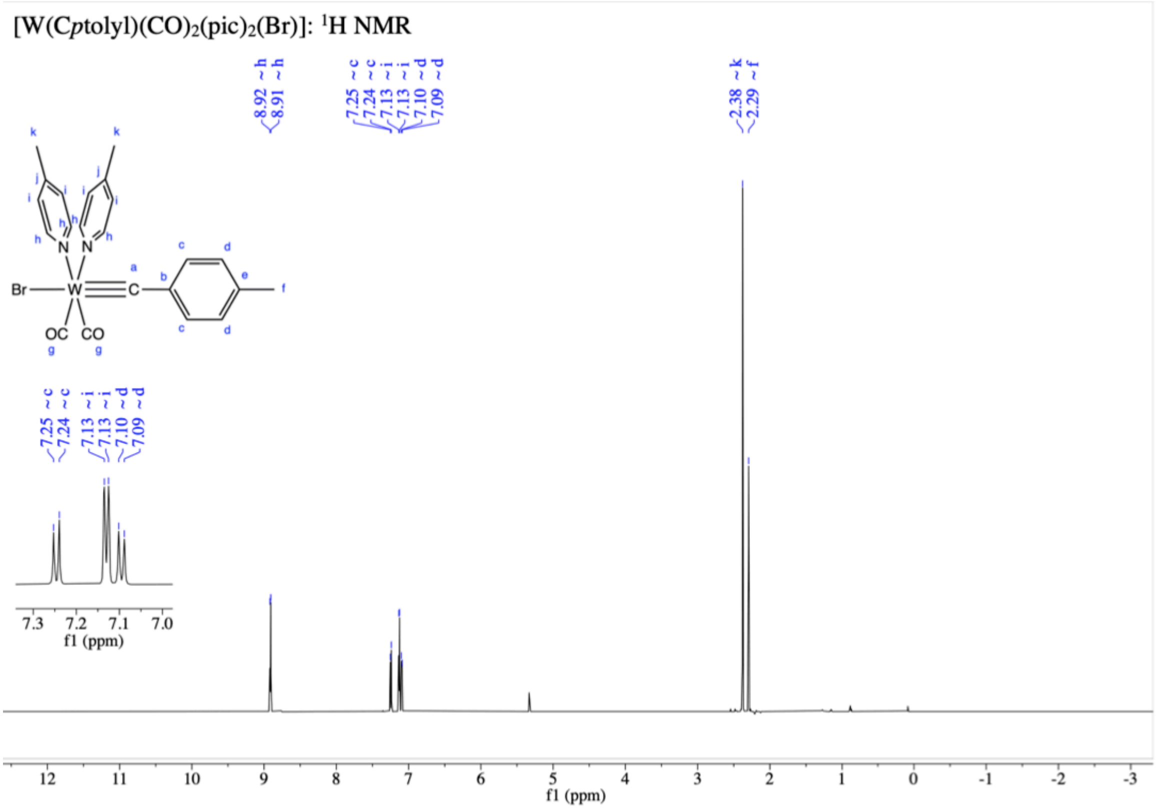

$^{13}C\{^1H\}$ NMR spectrum of [W(≡CC$_6$H$_4$Me-4)(CO)$_2$(pic)$_2$(Br)] (2a) (151 MHz, CD_2Cl_2, 298 K)

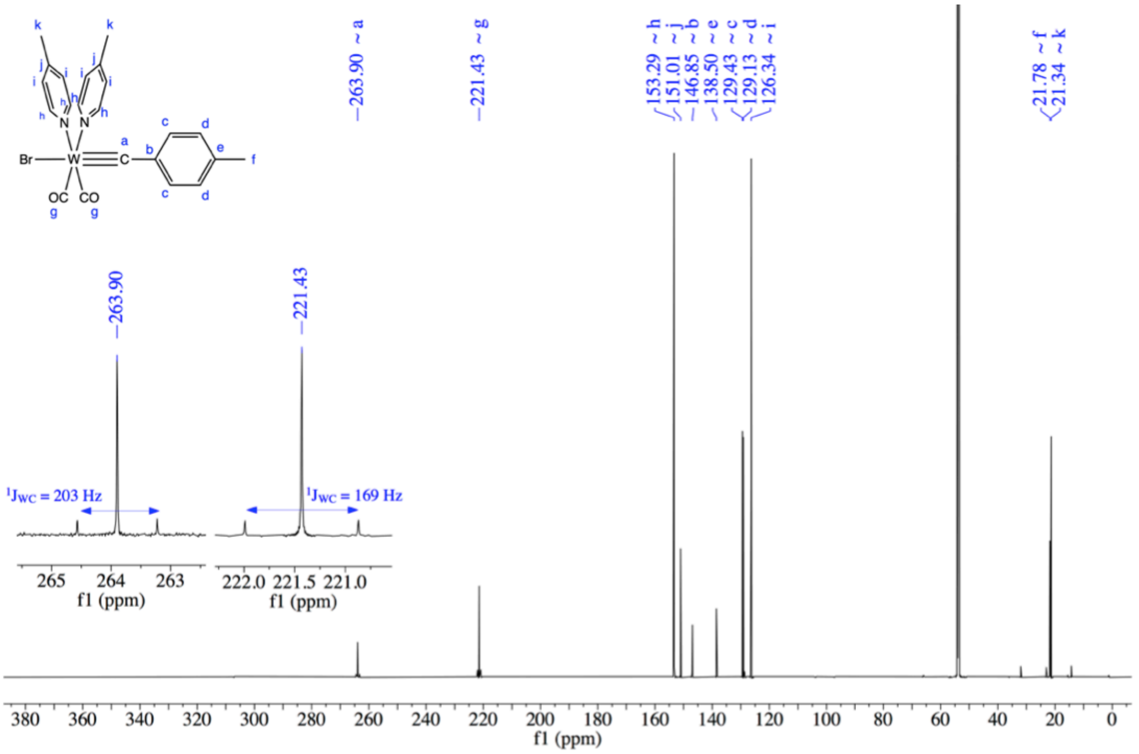

COSY NMR spectrum of [W(≡CC$_6$H$_4$Me-4)(CO)$_2$(pic)$_2$(Br)] (2a) (600 MHz, CD_2Cl_2, 298 K)

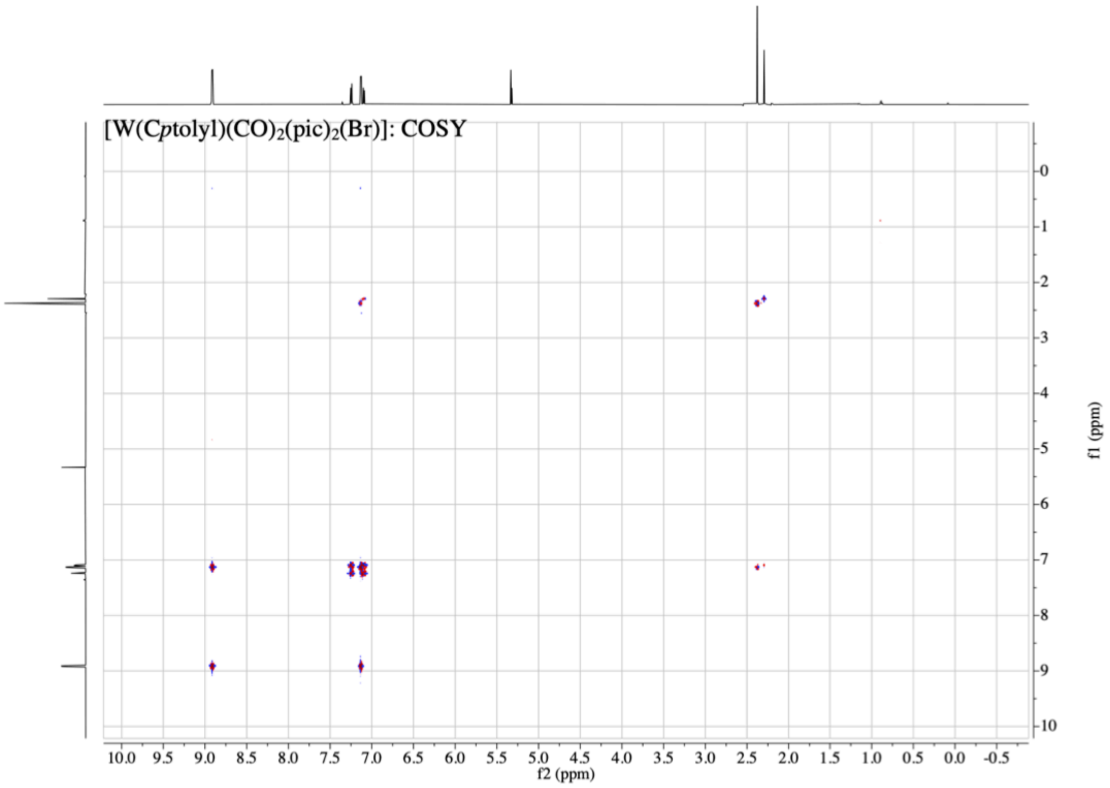

HSQC NMR spectrum of [W(≡CC$_6$H$_4$Me-4)(CO)$_2$(pic)$_2$(Br)] (2a) (600/151 MHz, CD$_2$Cl$_2$, 298 K)

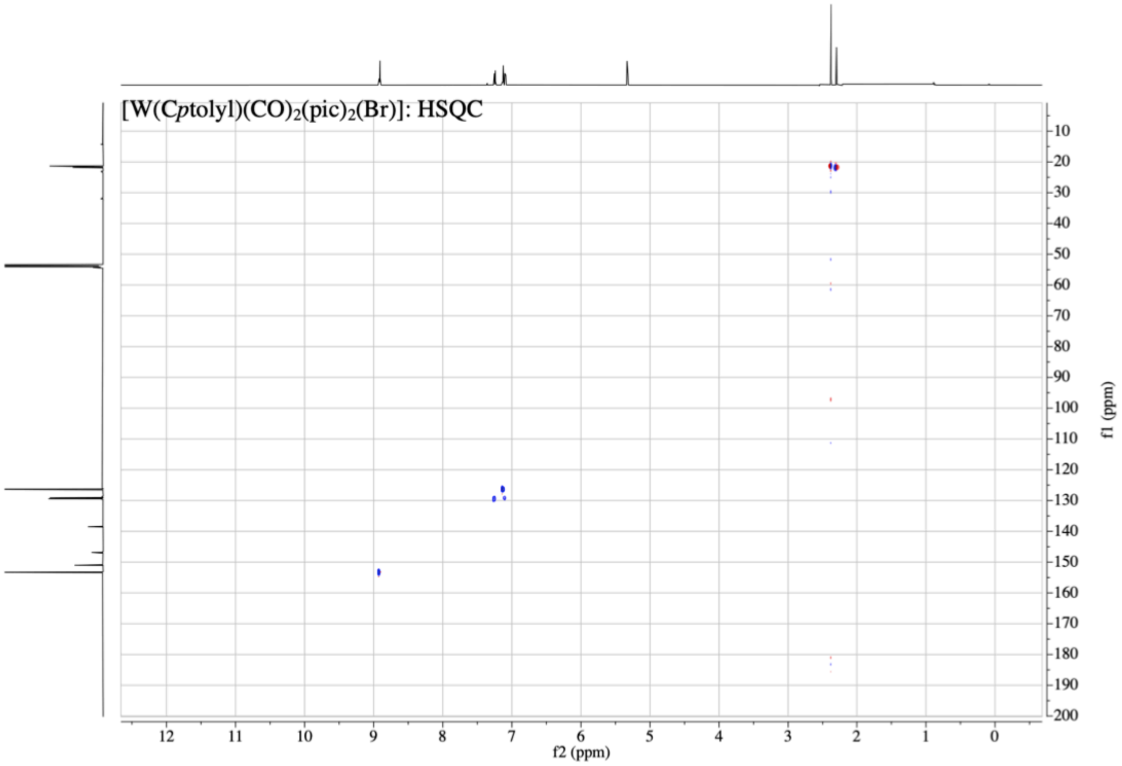

HMBC NMR spectrum of [W(≡CC$_6$H$_4$Me-4)(CO)$_2$(pic)$_2$(Br)] (2a) (600/151 MHz, CD$_2$Cl$_2$, 298 K)

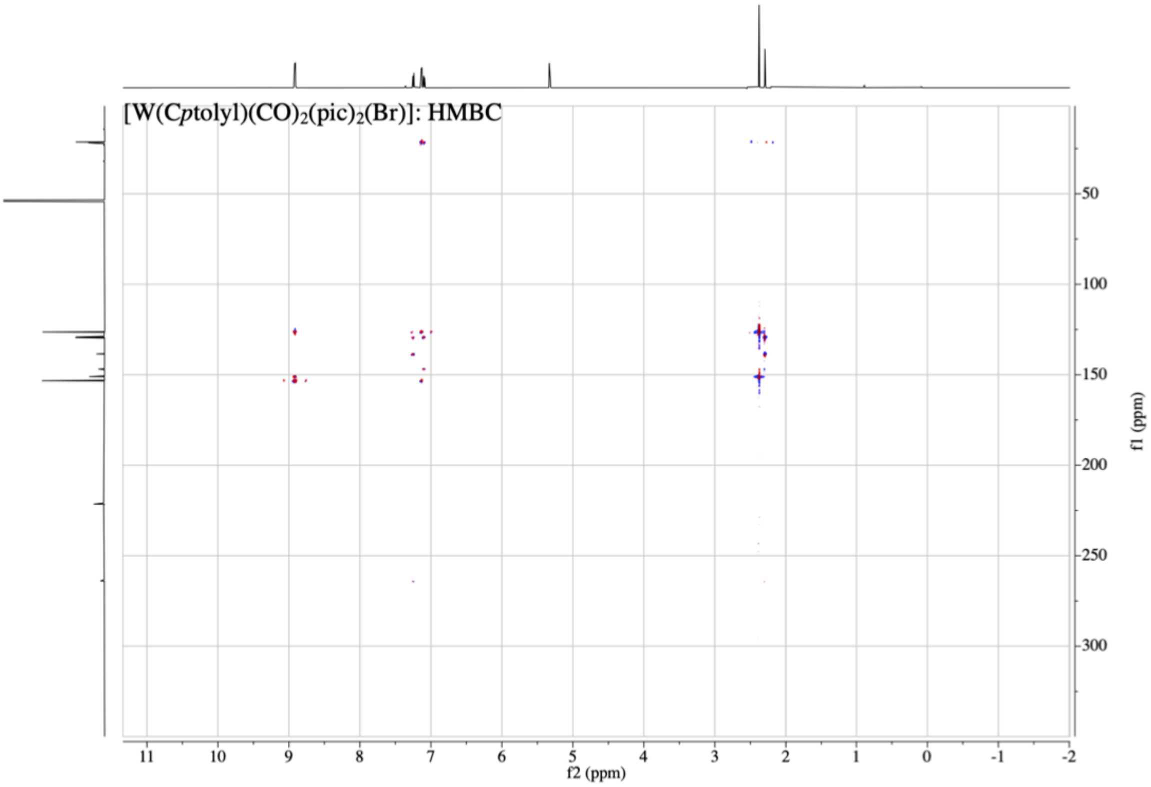

Solution IR spectrum of [W(≡CC_6H_4Me-4)$(CO)_2$$(pic)_2$(Br)] (2a) ($CH_2Cl_2$).

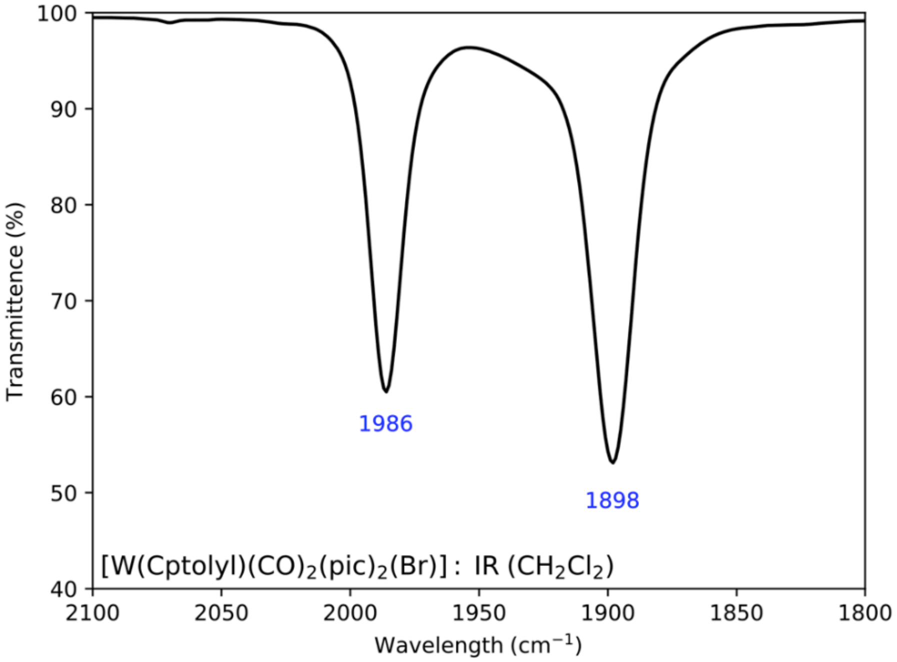

Solid State IR spectrum of [W(≡CC_6H_4Me-4)$(CO)_2$$(pic)_2$(Br)] (2a) (ATR).

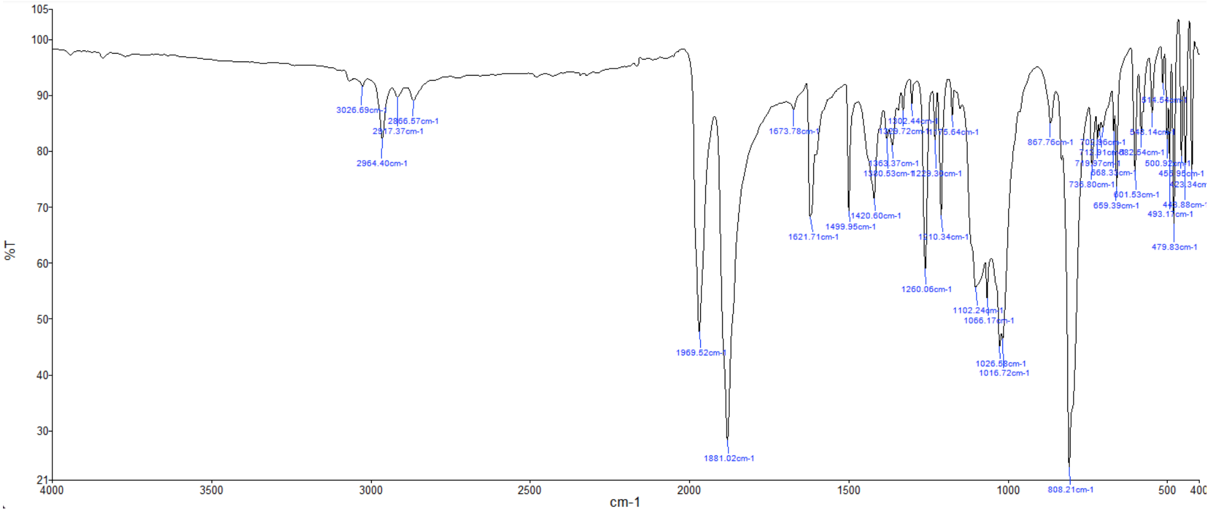

^{1}H NMR Spectrum of [Mo(≡CC$_6$H$_4$Me-4)(CO)$_2$(pic)$_2$Br] (2b) (800 MHz, CD$_2$Cl$_2$, 25 °)

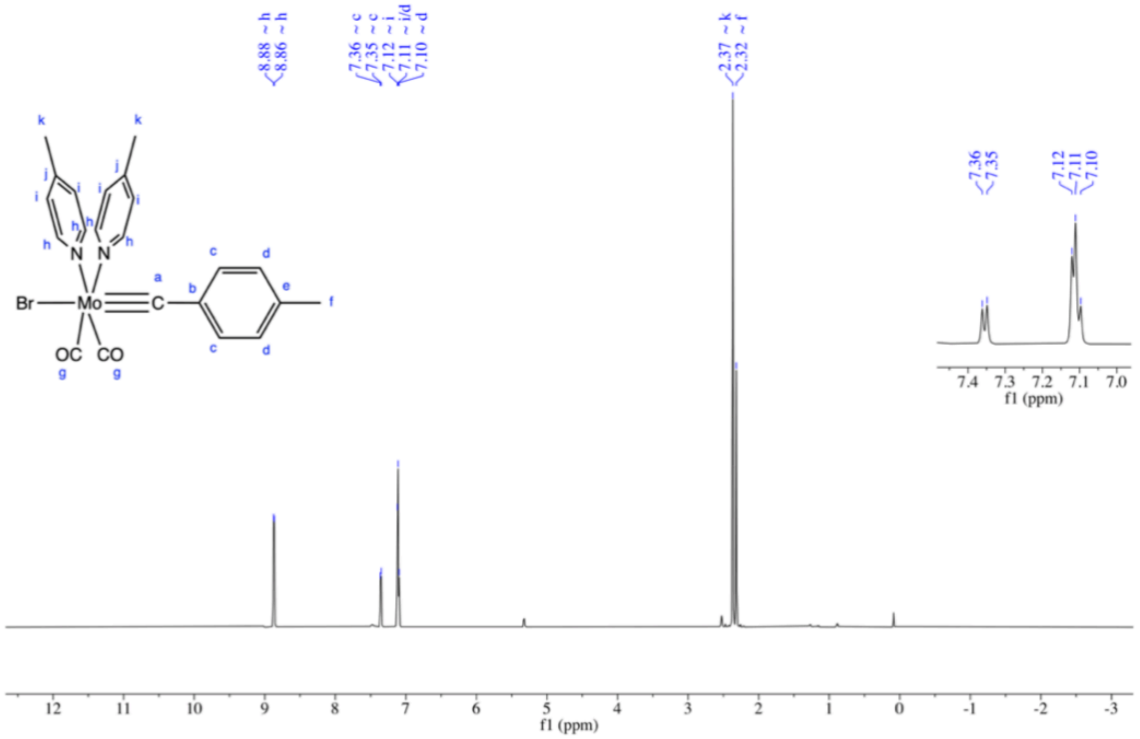

$^{13}C\{^{1}H\}$ NMR Spectrum of [Mo(≡CC$_6$H$_4$Me-4)(CO)$_2$(pic)$_2$Br] (2b) (151 MHz, CD$_2$Cl$_2$, 25 °)

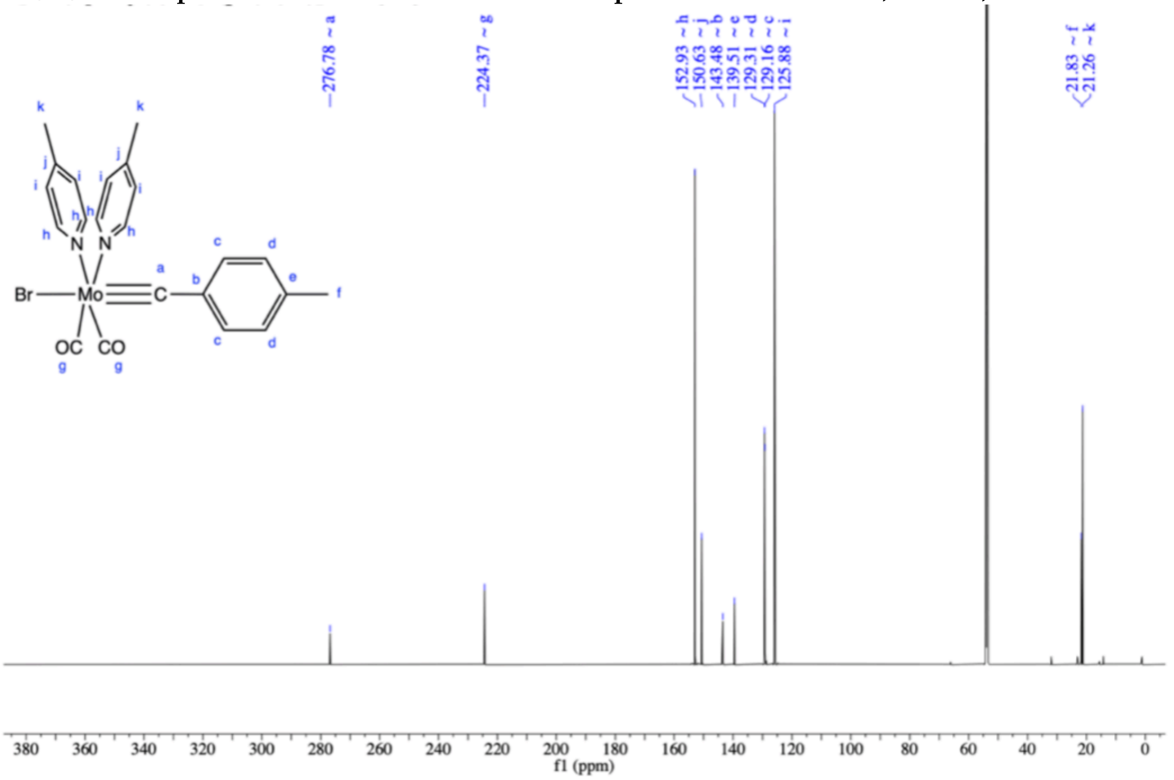

COSY NMR spectrum of [Mo(≡CC$_6$H$_4$Me-4)(CO)$_2$(pic)$_2$Br] (2b) (600 MHz, CD$_2$Cl$_2$, 298 K).

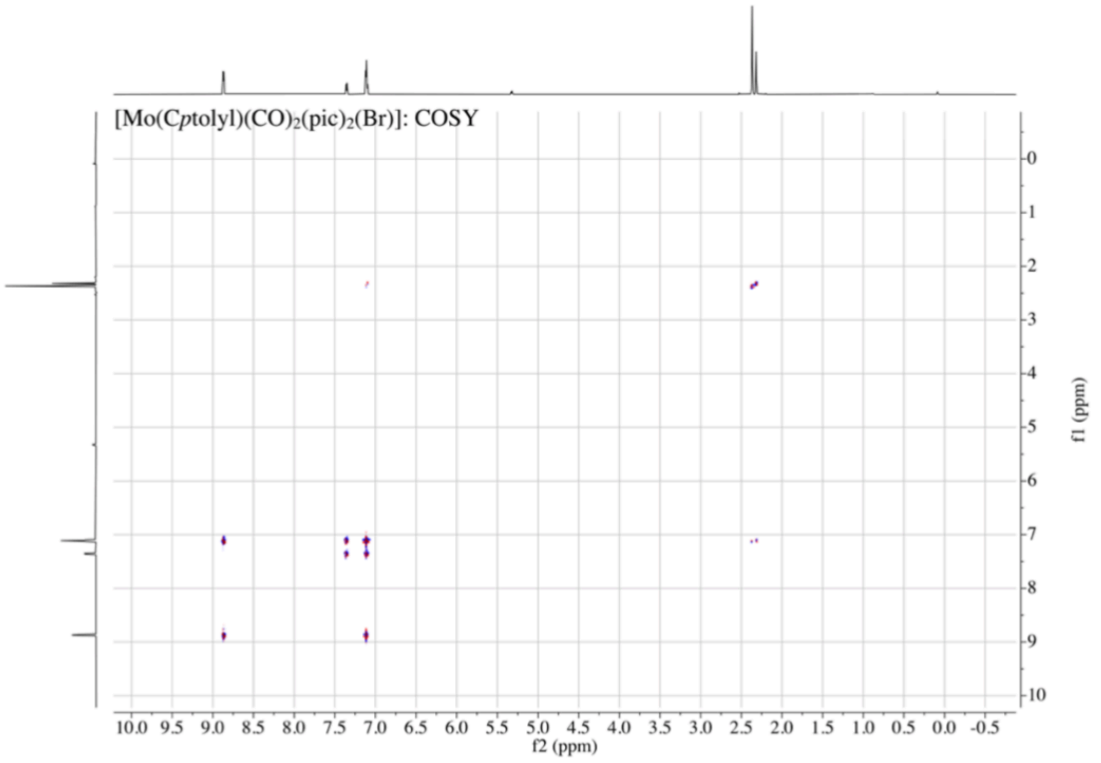

HSQC NMR spectrum of [Mo(≡CC$_6$H$_4$Me-4)(CO)$_2$(pic)$_2$Br] (2b) (600/151 MHz, CD$_2$Cl$_2$, 298 K)

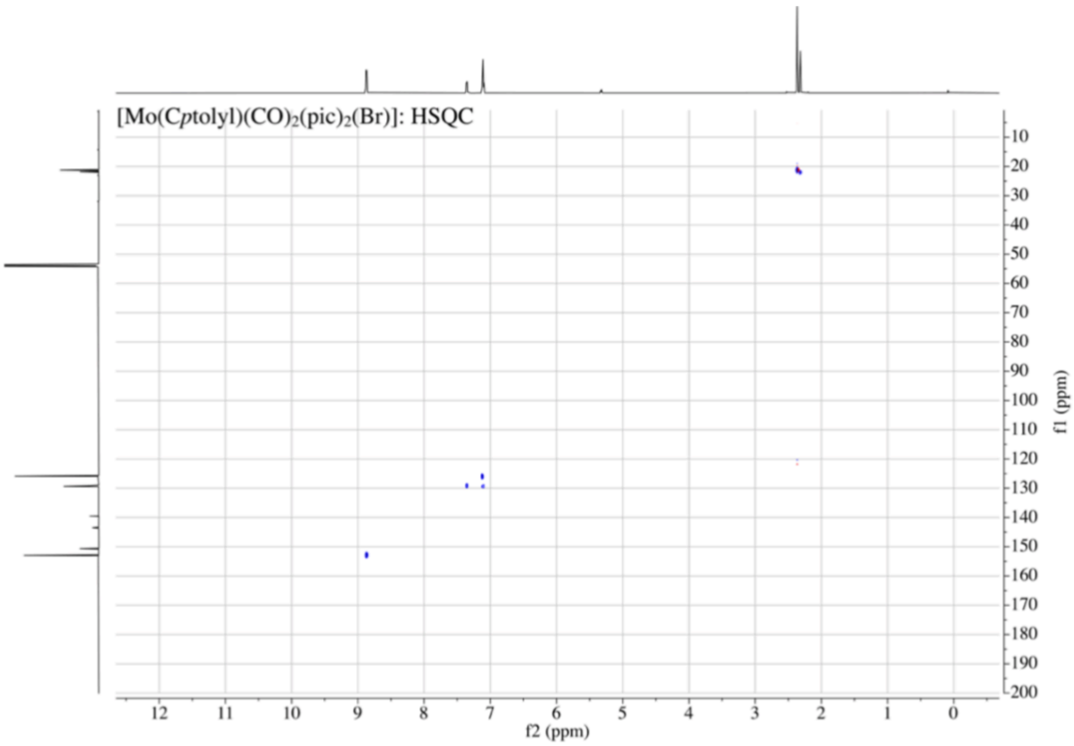

HMBC NMR spectrum of [Mo(≡CC$_6$H$_4$Me-4)(CO)$_2$(pic)$_2$Br] (2b) (600/151 MHz, CD_2Cl_2, 298 K)

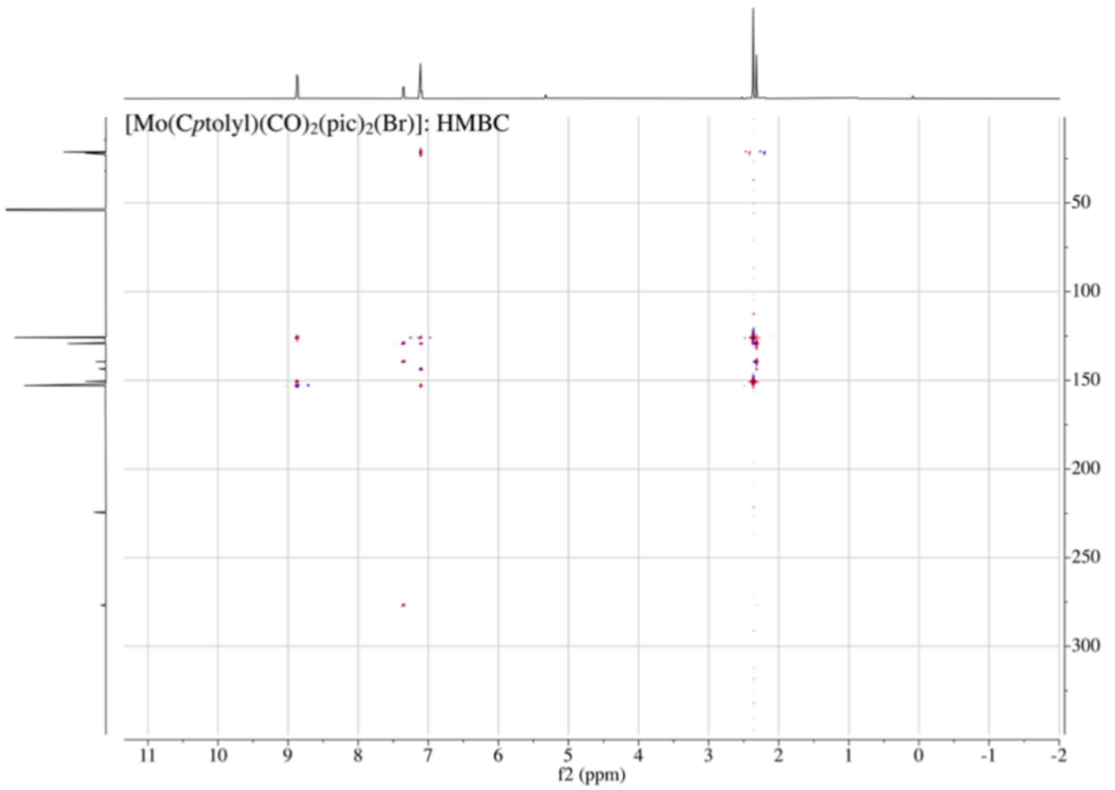

Solution IR spectrum of [Mo(≡CC$_6$H$_4$Me-4)(CO)$_2$(pic)$_2$Br] (2b) (CH_2Cl_2).

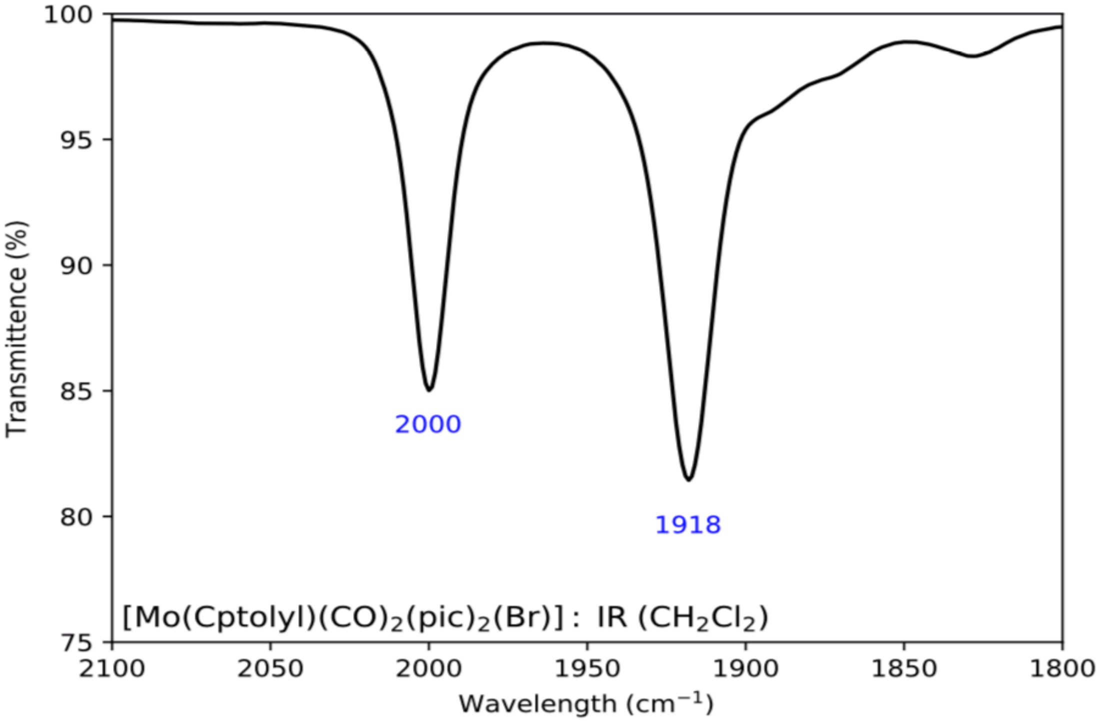

Solid State IR spectrum of [Mo(≡CC_6H_4Me-4)$(CO)_2(pic)_2Br$] (2b) (ATR).

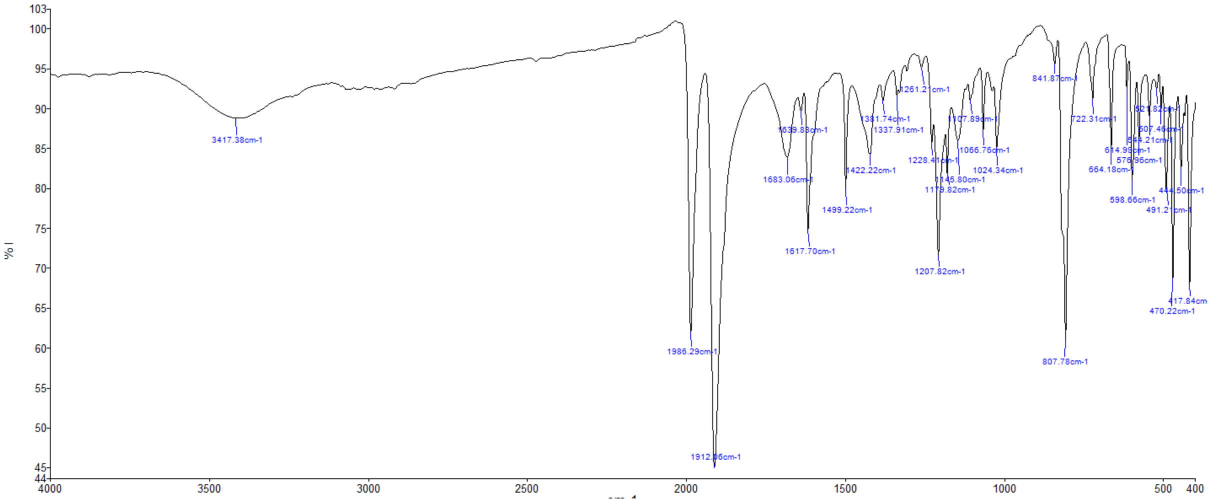

^{1}H NMR Spectrum of [W(≡CC_6H_4Me-4)$(CO)_2${HB(ImMe)$_3$}] (4) (800 MHz, CD_2Cl_2, 25 °)

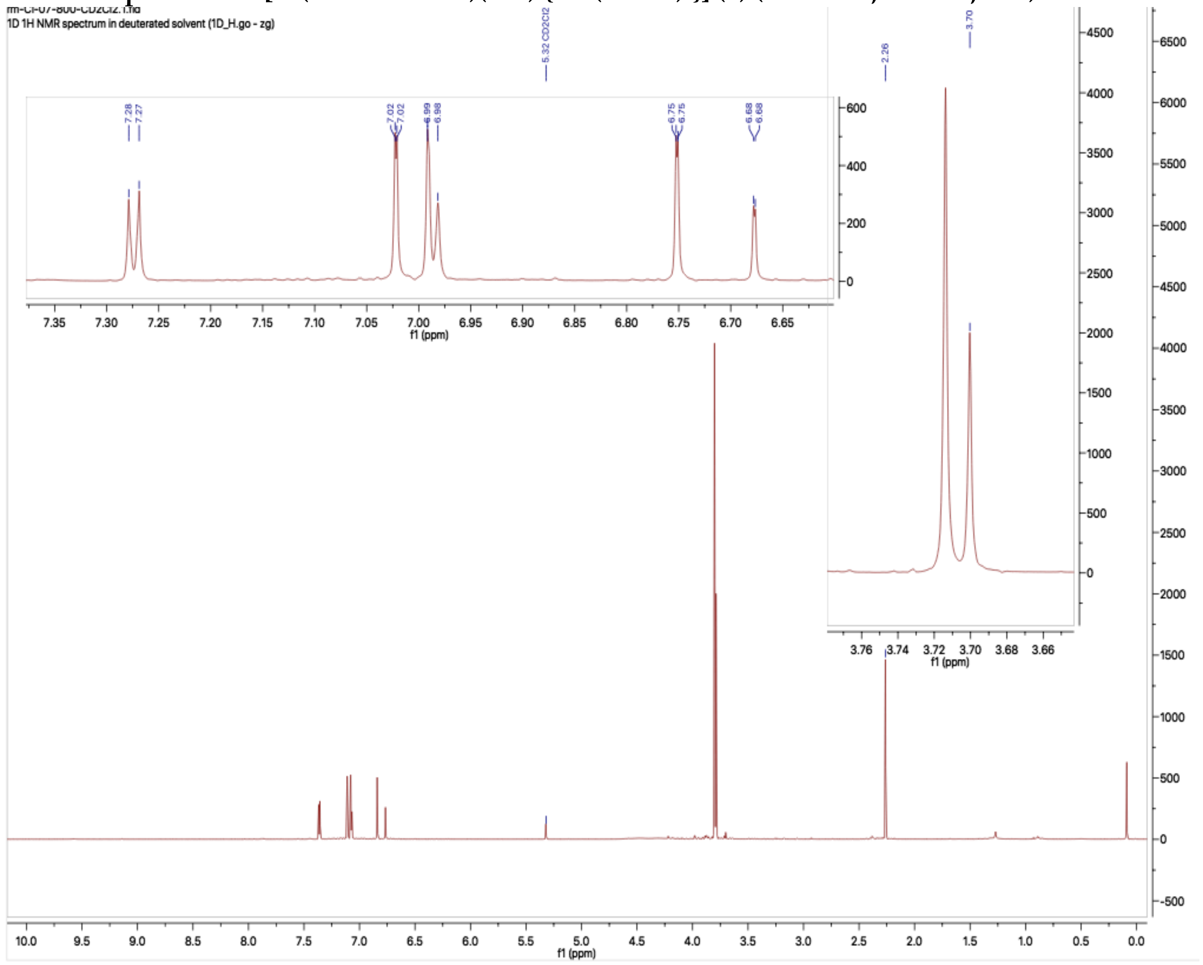

$^{13}C\{^1H\}$ NMR Spectrum of [W(≡CC$_6$H$_4$Me-4)(CO)$_2${HB(ImMe)$_3$}] (4) (201 MHz, CD$_2$Cl$_2$, 25 °)

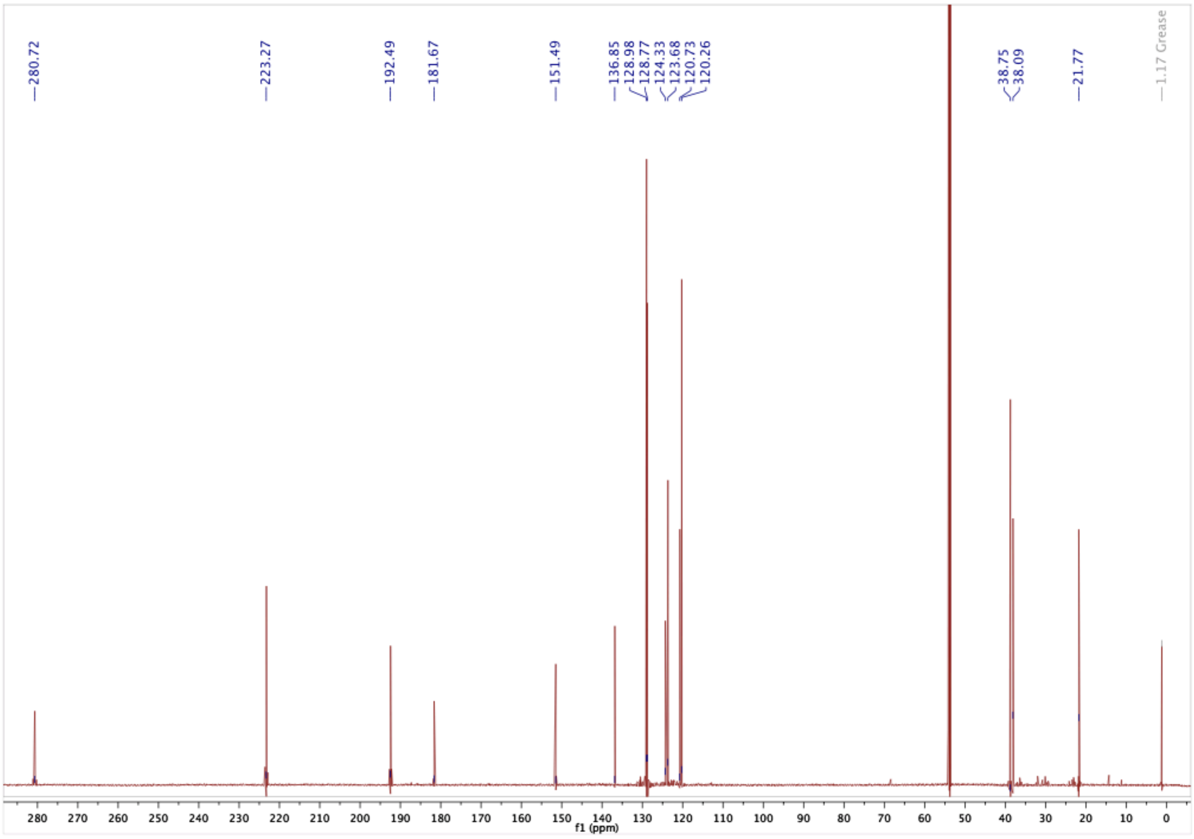

$^{13}C\{^1H\}$ NMR Spectrum of [W(≡CC$_6$H$_4$Me-4)(CO)$_2${HB(ImMe)$_3$}] (4) (201 MHz, CD$_2$Cl$_2$, 25 °) – Low field extracts

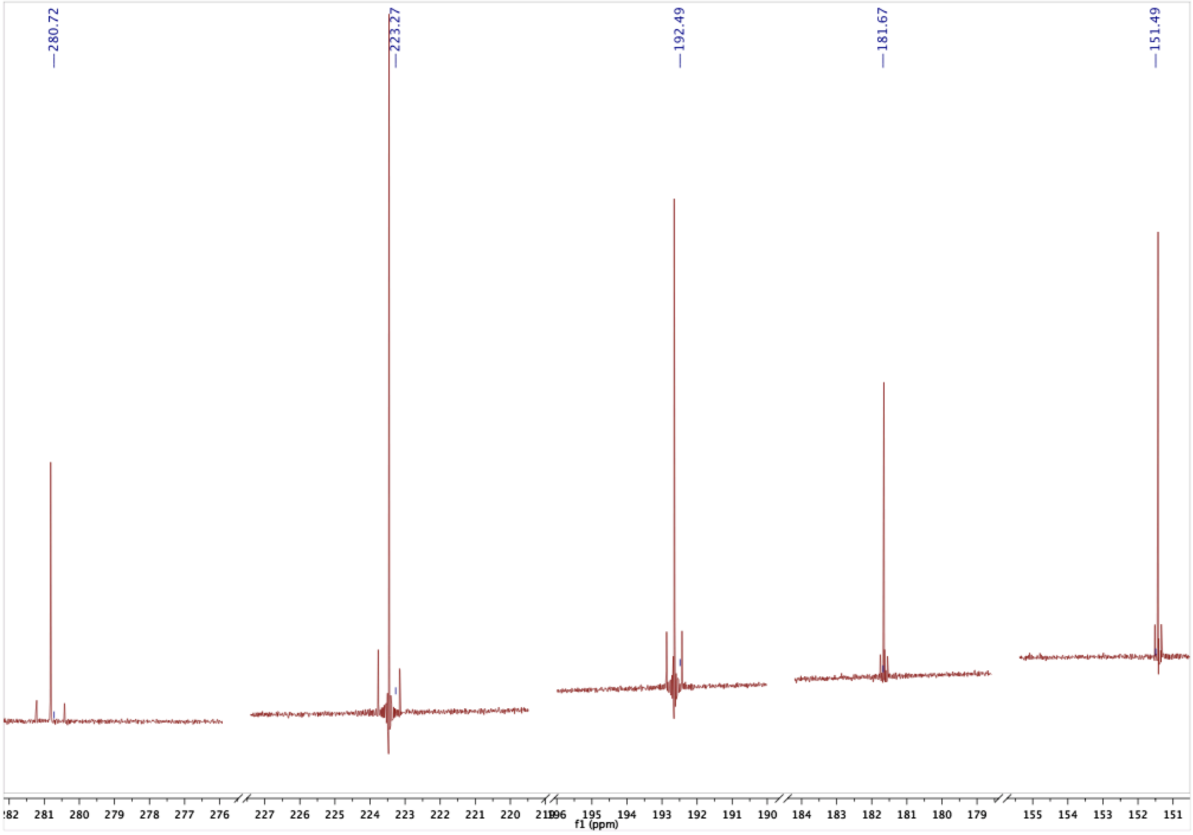

Solid State IR Spectrum of [W(≡CC$_6$H$_4$Me-4)(CO)$_2${HB(ImMe)$_3$}] (4) (ATR)

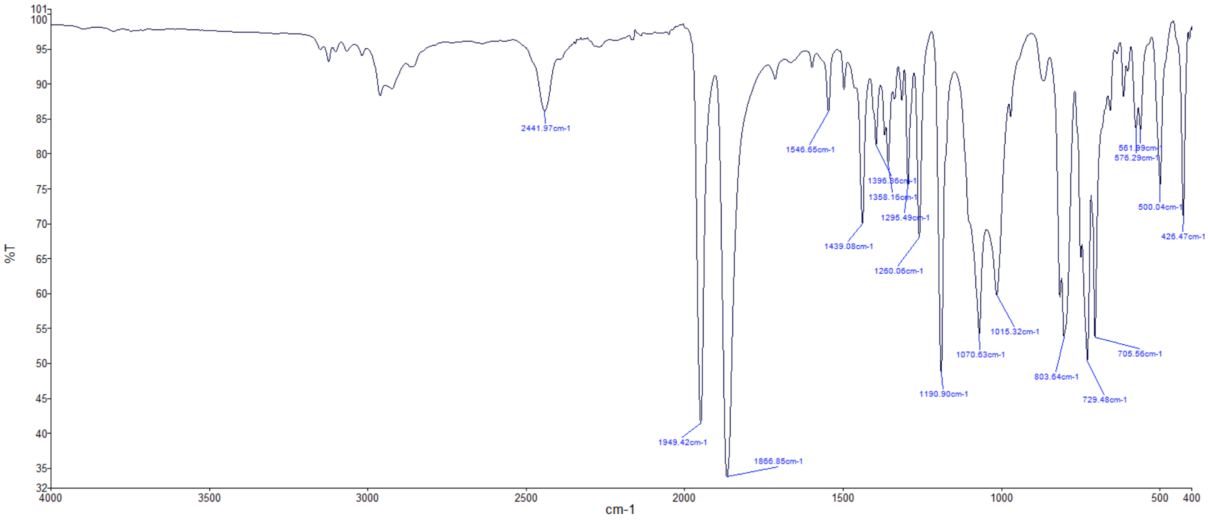

Solution IR Spectrum of [W(≡CC$_6$H$_4$Me-4)(CO)$_2${HB(ImMe)$_3$}] (4) (CH$_2$Cl$_2$)

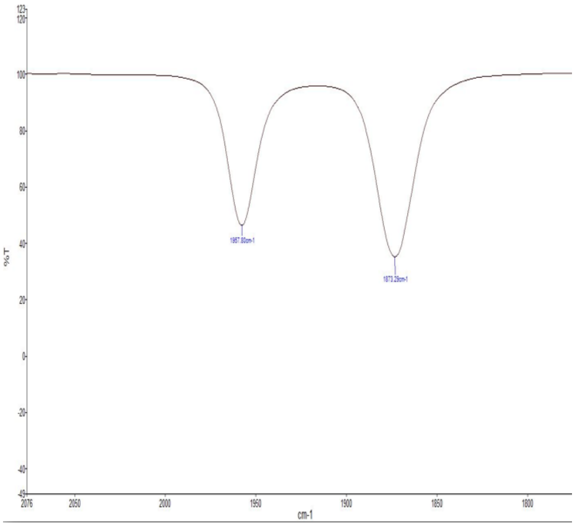

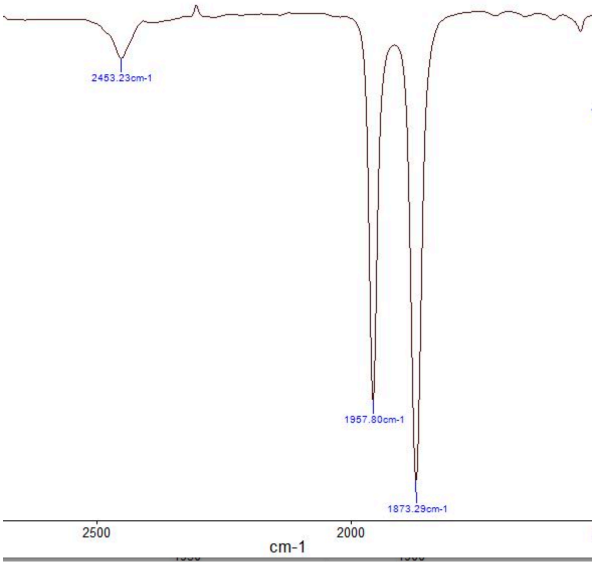

High Resolution ESI-MS of $[W(\equiv CC_6H_4Me\text{-}4)(CO)_2\{HB(ImMe)_3\}]$ (4) (MeOH)

Elemental Composition Report

Single Mass Analysis

Tolerance = 3.0 PPM / DBE: min = -1.5, max = 18.0
Element prediction: Off
Number of isotope peaks used for i-FIT = 3

Monoisotopic Mass, Odd and Even Electron Ions
364 formula(e) evaluated with 1 results within limits (up to 50 best isotopic matches for each mass)
Elements Used:
C: 0-50 H: 0-50 11B: 0-1 N: 0-6 O: 0-2 184W: 0-1

CI-7-WCTol/AJ SYNAPTG2-Si#NotSet 23-Sep-2022
67213 15:10:05
3547 87 (0.192) Cm (80:121)
1: TOF MS ES+
1.87e+007

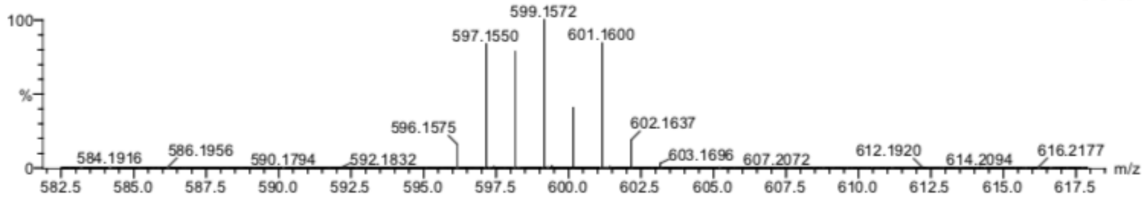

Minimum:				-1.5		
Maximum:		5.0	3.0	18.0		
Mass	Calc. Mass	mDa	PPM	DBE	i-FIT	Formula
599.1572	599.1563	0.9	1.5	16.0	2745.8	C22 H24 11B N6 O2 184W

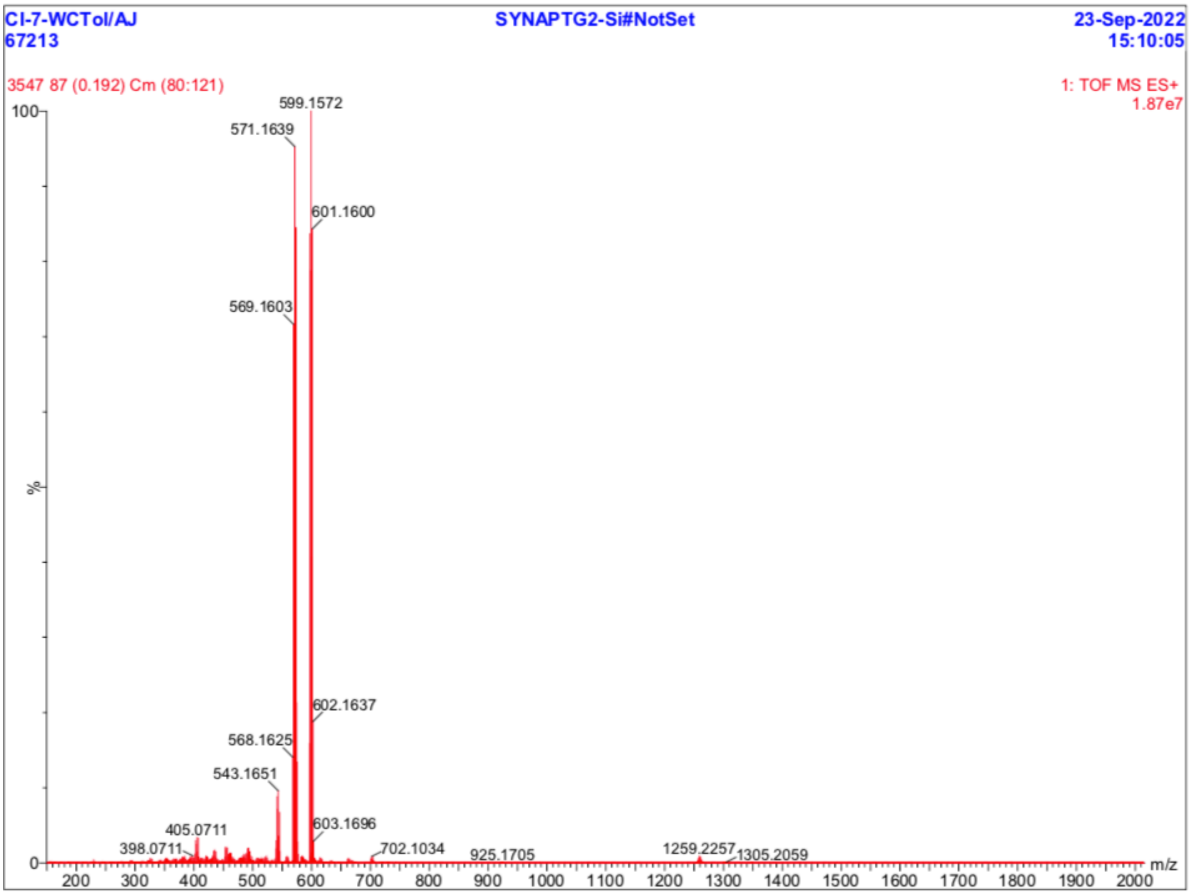

^{1}H NMR Spectrum of [W(≡CC$_6$H$_4$Me-4)(CO)$_2${HB(pzMe$_2$)$_3$}] (5) (400 MHz, CDCl$_3$, 25 °)

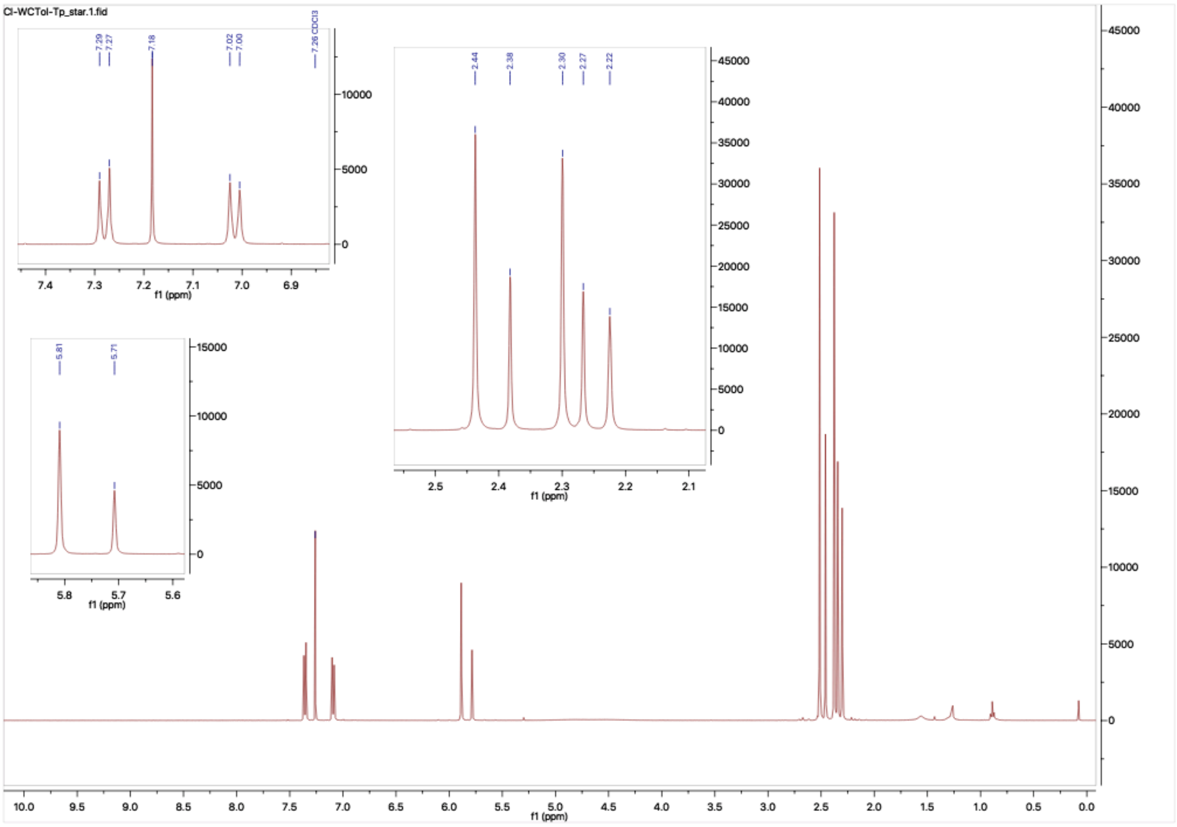

^{13}C{^{1}H} NMR Spectrum of [W(≡CC$_6$H$_4$Me-4)(CO)$_2${HB(pzMe$_2$)$_3$}] (5) (201 MHz, CDCl$_3$, 25 °)

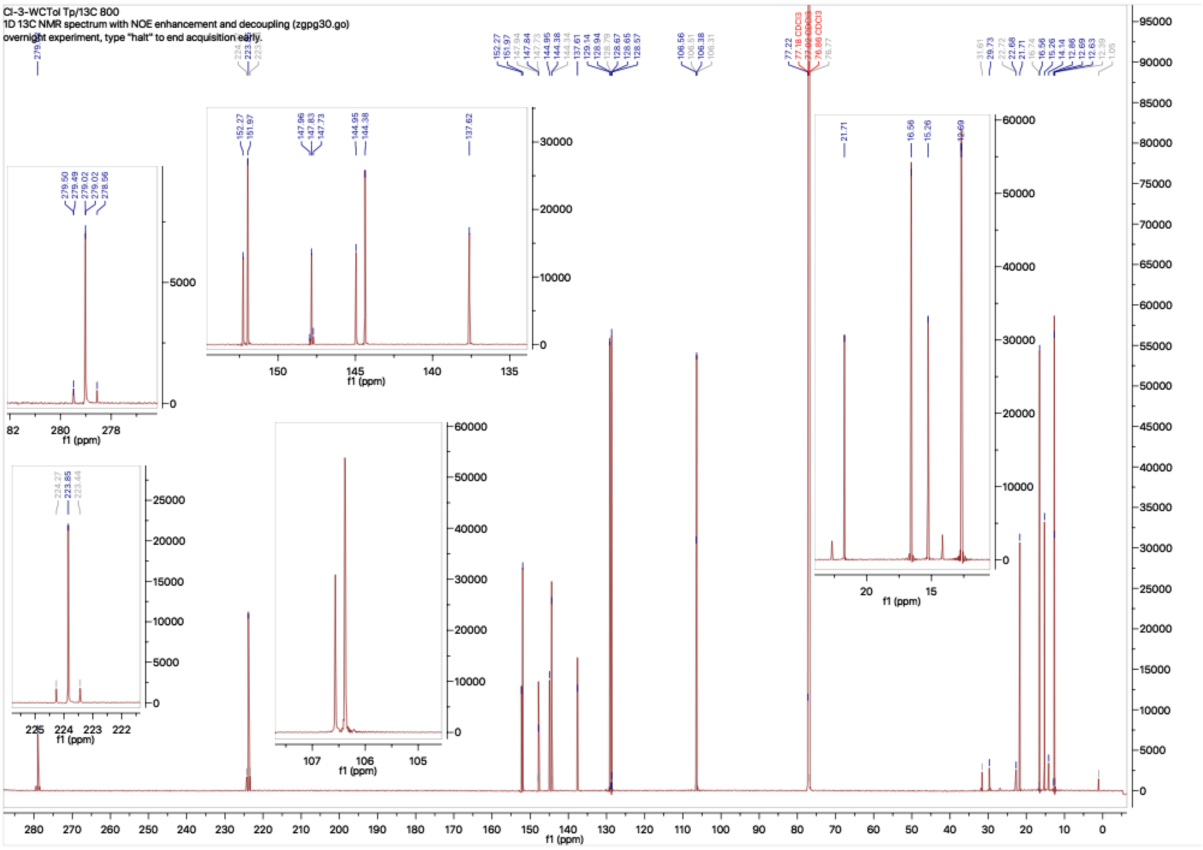

$^{11}B\{^1H\}$ NMR Spectrum of $[W(\equiv CC_6H_4Me\text{-}4)(CO)_2\{HB(pzMe_2)_3\}]$ (5) (128 MHz, $CDCl_3$, 25 °)

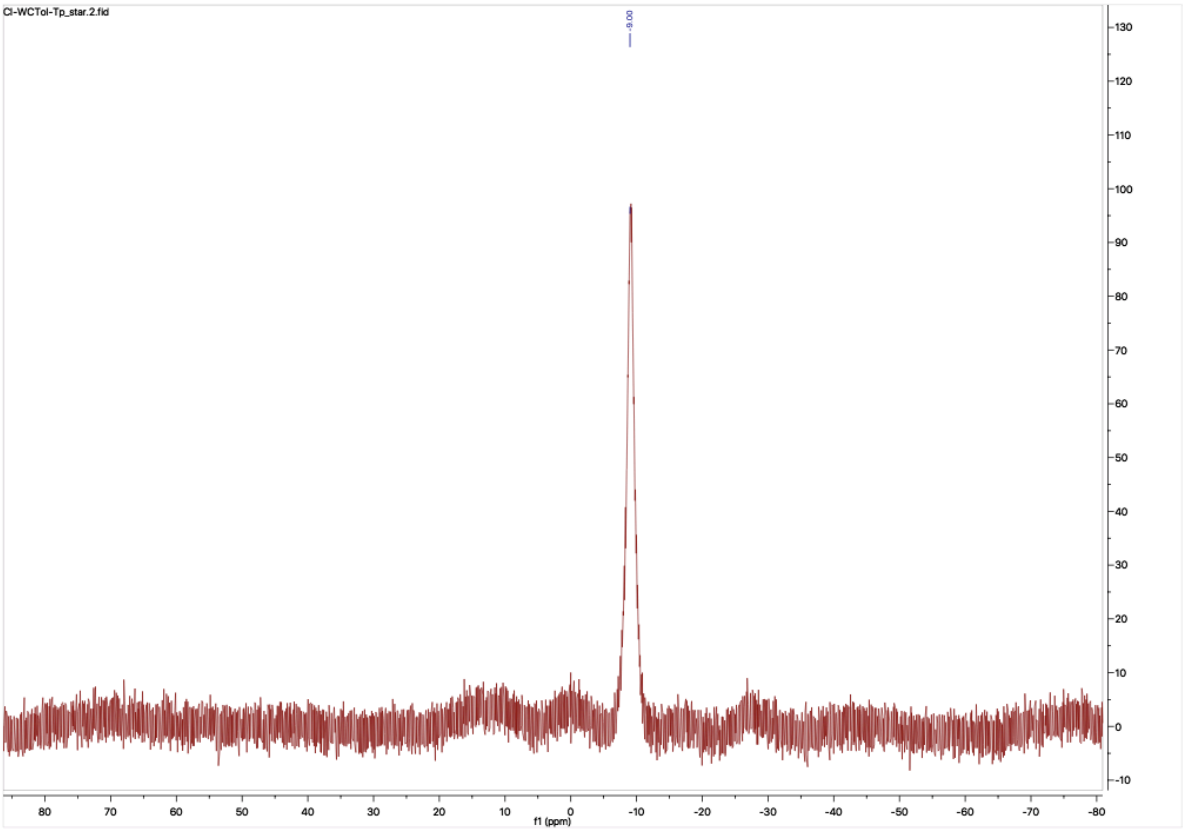

High Resolution ESI-MS of $[W(\equiv CC_6H_4Me\text{-}4)(CO)_2\{HB(pzMe_2)_3\}]$ (5) (MeOH)

Elemental Composition Report **Page 1**

Single Mass Analysis
Tolerance = 3.0 PPM / DBE: min = -1.5, max = 18.0
Element prediction: Off
Number of isotope peaks used for i-FIT = 3

Monoisotopic Mass, Odd and Even Electron Ions
351 formula(e) evaluated with 1 results within limits (up to 50 best isotopic matches for each mass)
Elements Used:
C: 0-50 H: 0-50 11B: 0-1 N: 0-6 O: 0-2 184W: 0-1

LJW-CI/AJ
67290
3782 87 (0.192) Cm (87:94)
1: TOF MS ES+

SYNAPTG2-Si#NotSet

14-Oct-2022
09:53:47

1.89e+005

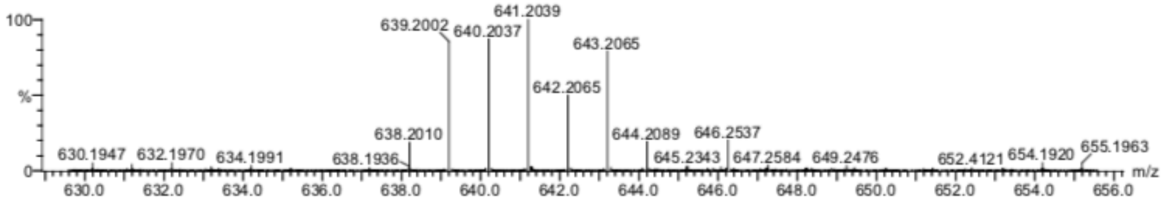

Minimum:				-1.5		
Maximum:		5.0	3.0	18.0		

Mass	Calc. Mass	mDa	PPM	DBE	i-FIT	Formula
641.2039	641.2033	0.6	0.9	16.0	1750.0	C25 H30 11B N6 O2 184W

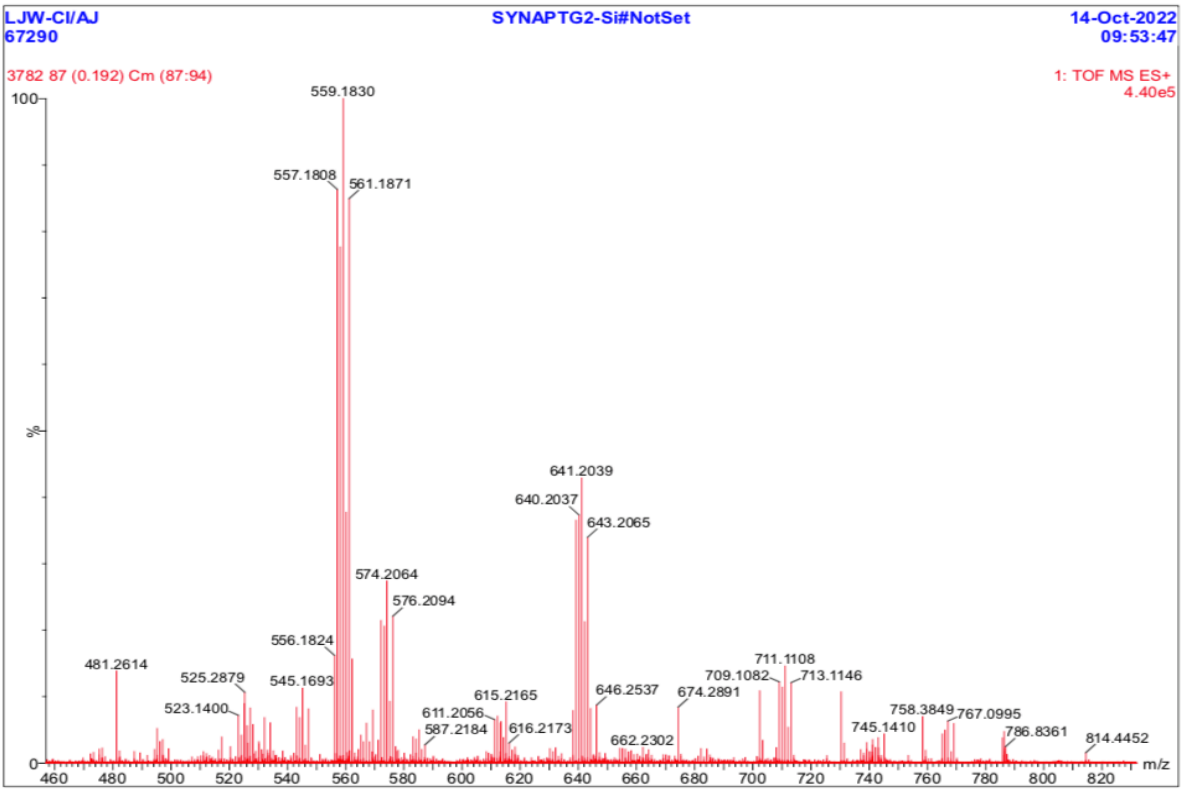

LJW-Cl/AJ
67290
SYNAPTG2-Si#NotSet
14-Oct-2022
09:53:47
3782 87 (0.192) Cm (87:94)
1: TOF MS ES+
4.40e5
100
%
0
559.1830
557.1808
561.1871
641.2039
640.2037
643.2065
574.2064
576.2094
556.1824
481.2614
525.2879
545.1693
523.1400
711.1108
709.1082
713.1146
646.2537
674.2891
615.2165
611.2056
587.2184
616.2173
662.2302
758.3849
767.0995
745.1410
786.8361
814.4452
m/z
460
480
500
520
540
560
580
600
620
640
660
680
700
720
740
760
780
800
820

Solid State IR Spectrum of [W(≡CC$_6$H$_4$Me-4)(CO)$_2${HB(ImMe)$_3$}] (4) (ATR)

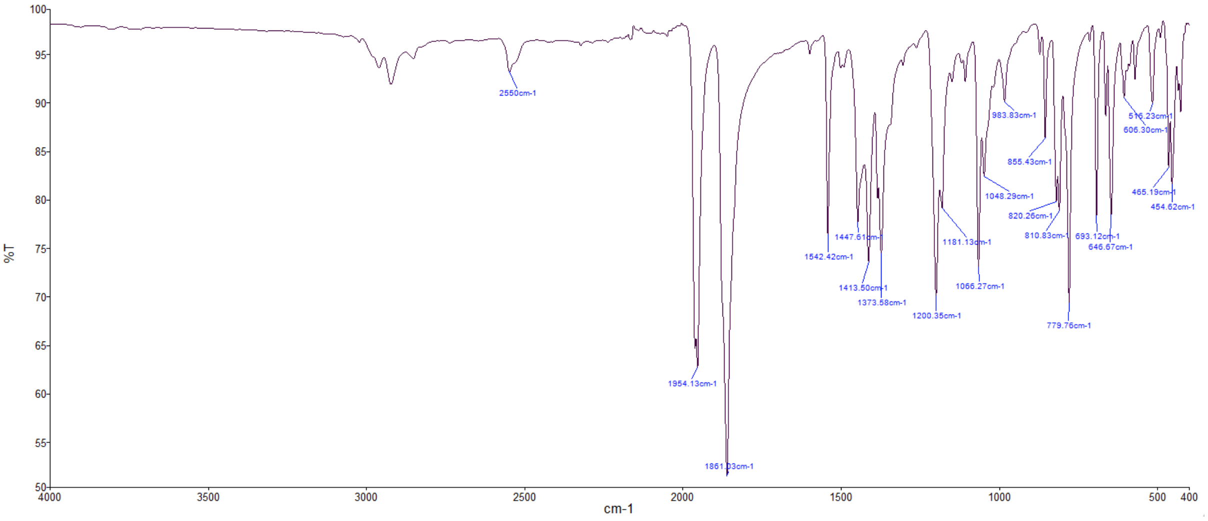

Solution IR Spectrum of [W(≡CC$_6$H$_4$Me-4)(CO)$_2${HB(ImMe)$_3$}] (4) (CH$_2$Cl$_2$)

PerkinElmer Spectrum Version 10.4.3
Wednesday, 28 September 2022 5:33 PM

Analyst Administrator
Date Wednesday, 28 September 2022 5:33 PM

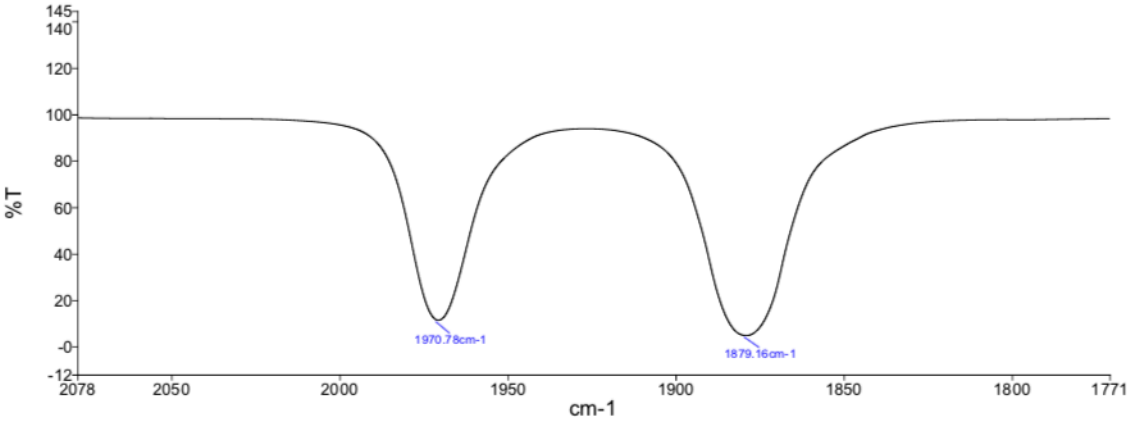

Sample Name	Description	Quality Checks
Cl-WCTol-Tp_star	Sample 3669 By Administrator Date Wednesday, September 28 2022	The Quality Checks give rise to multiple warnings for the sample.

^{1}H NMR Spectrum of [WAu(μ–CC$_6$H$_4$Me-4)Cl(CO)$_2${HB(ImMe)$_3$}] (6) (600 MHz, CD$_2$Cl$_2$, 25 °)

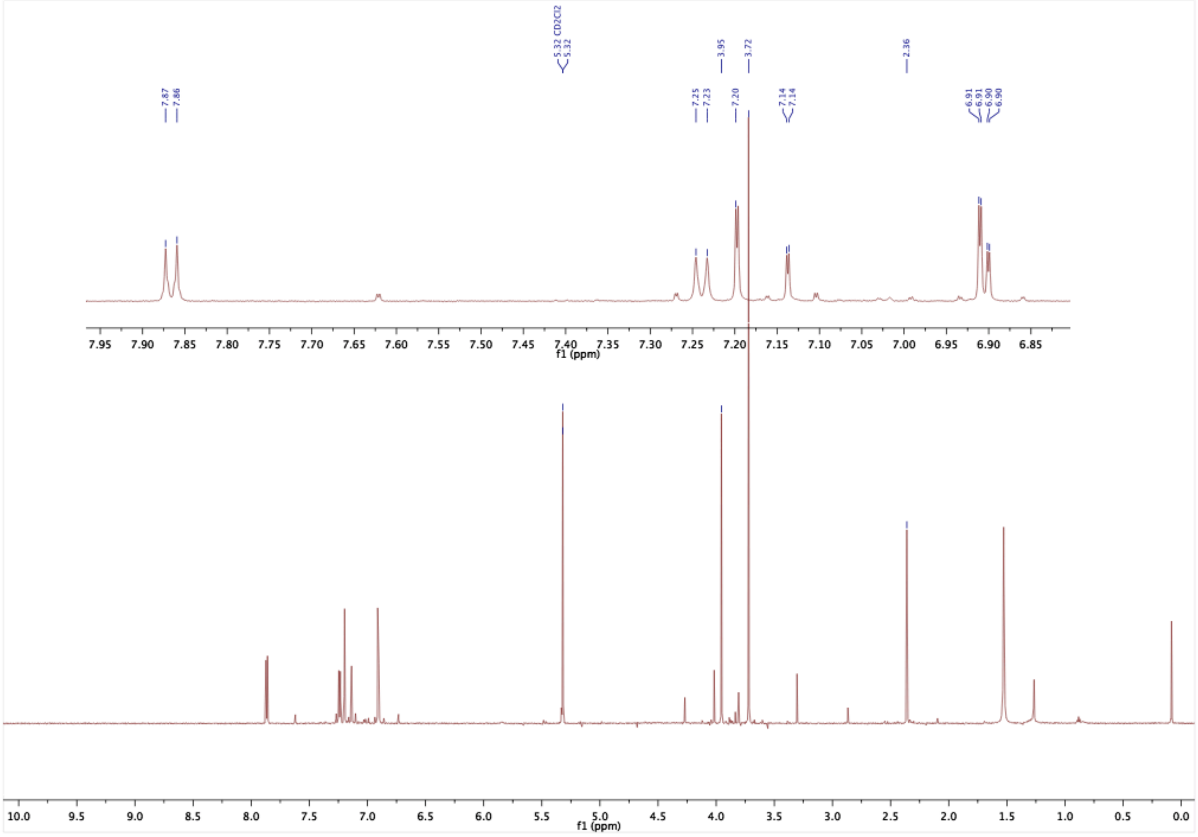

^{13}C{^{1}H} NMR Spectrum of [WAu(μ–CC$_6$H$_4$Me-4)Cl(CO)$_2${HB(ImMe)$_3$}] (6) (151 MHz, CD$_2$Cl$_2$, 25 °)

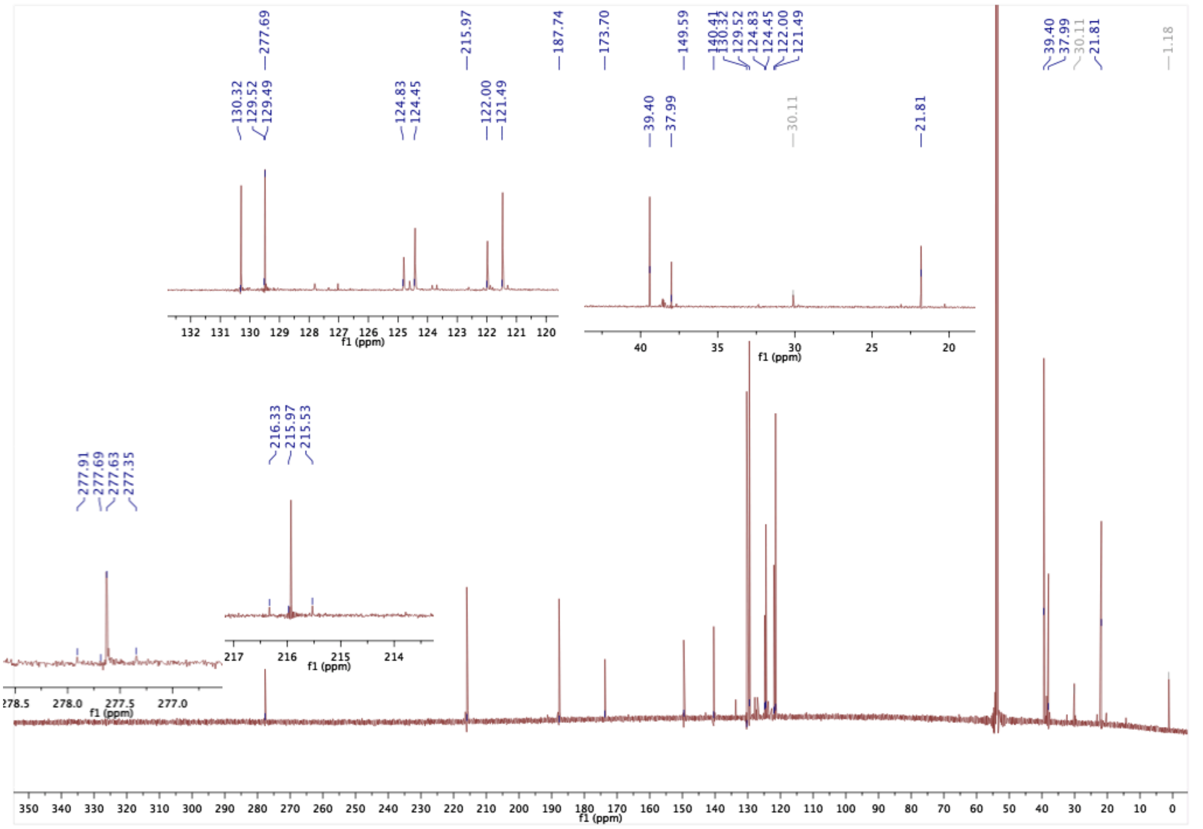

Solid State IR Spectrum of [WAu(μ–CC$_6$H$_4$Me-4)Cl(CO)$_2${HB(ImMe)$_3$}] (6) (ATR)

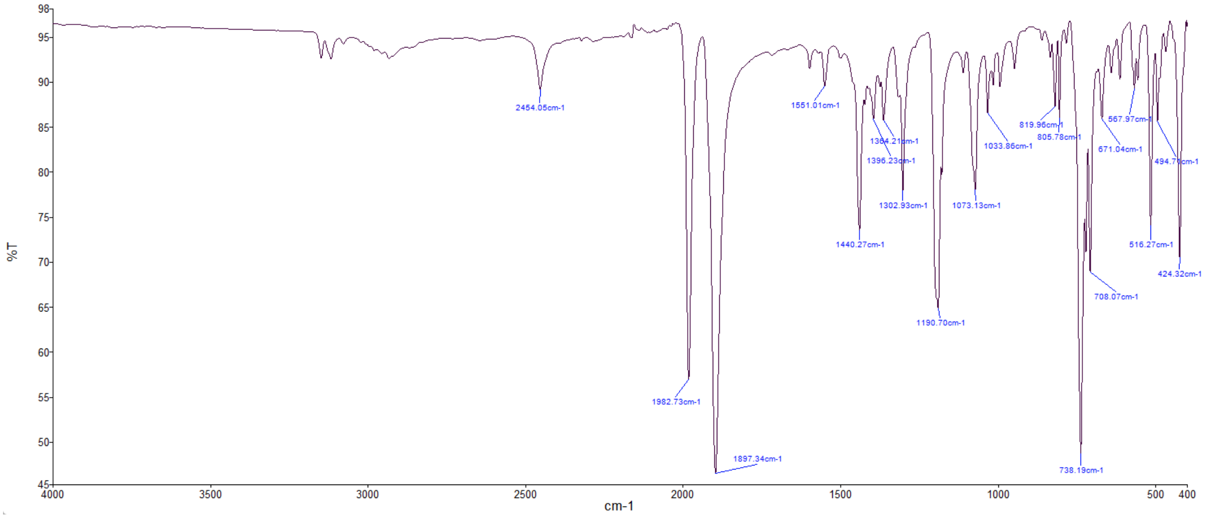

Solution IR Spectrum of [WAu(μ–CC$_6$H$_4$Me-4)Cl(CO)$_2${HB(ImMe)$_3$}] (6) (CH$_2$Cl$_2$)

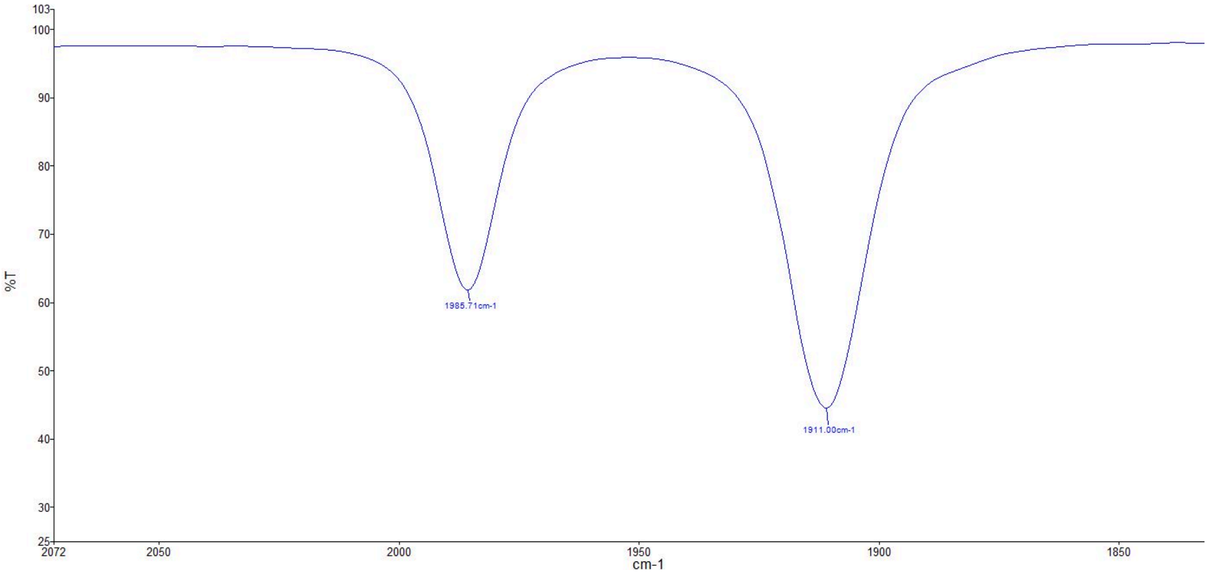

^{11}B NMR Spectrum of [WAu(μ–CC_6H_4Me-4)Cl$(CO)_2${HB$(ImMe)_3$}] (6) (128 MHz, CD_2Cl_2, 25 °)

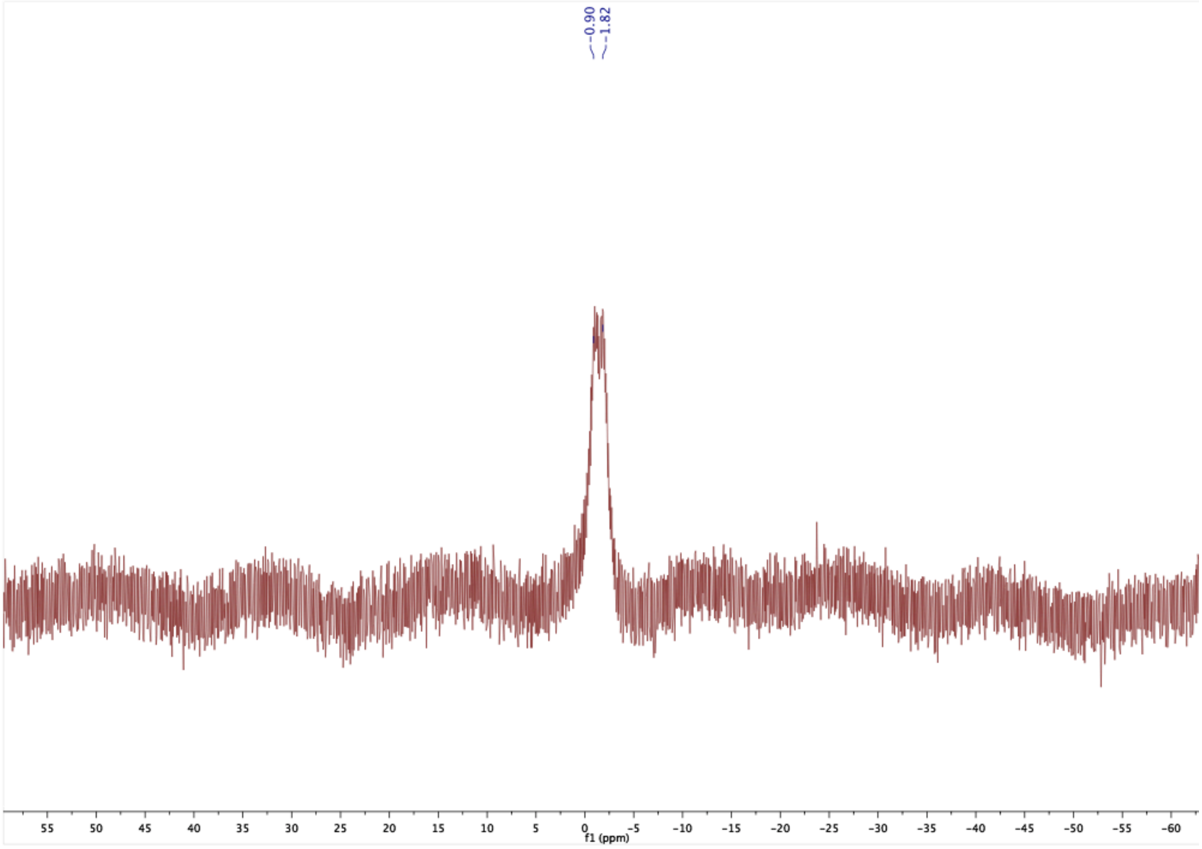

High Resolution ESI-MS of [WAu(μ–CC_6H_4Me-4)Cl$(CO)_2${HB$(ImMe)_3$}] (6) (MeOH)

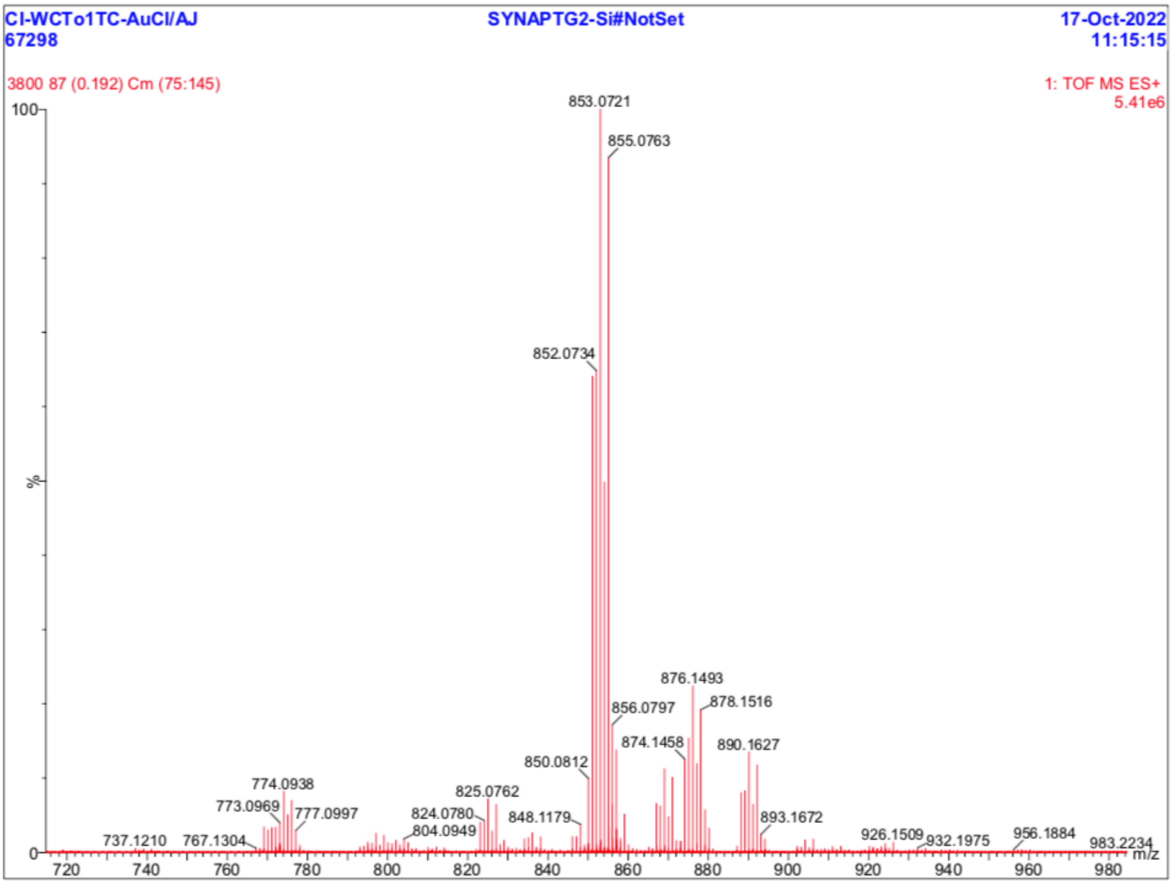

Supplement: Supplementary file 1 [file molecules-28-07761-s001.zip › molecules-2738534-supplementary.pdf]
